# Effectiveness and cost-effectiveness of text messages with or without endowment incentives for weight management in men with obesity (Game of Stones): study protocol for a randomised controlled trial

**DOI:** 10.1186/s13063-022-06504-5

**Published:** 2022-07-22

**Authors:** Lisa Macaulay, Catriona O’Dolan, Alison Avenell, Paula Carroll, Seonaidh Cotton, Stephan Dombrowski, Andrew Elders, Beatriz Goulao, Cindy Gray, Fiona M. Harris, Kate Hunt, Frank Kee, Graeme MacLennan, Matthew David McDonald, Michelle McKinley, Rebecca Skinner, Claire Torrens, Martin Tod, Katrina Turner, Marjon van der Pol, Pat Hoddinott

**Affiliations:** 1 NMAHP Research Unit, Stirling University, Pathfoot Building, Stirling, FK9 4LA UK; 2 Health Services Research Unit, 3Rd Floor Health Sciences Building, Foresterhill, Aberdeen, AB25 2ZD UK; 3 Department Sport & Exercise Science, Waterford Institute of Technology, Main Campus Cork RoadCo. Waterford, Waterford City, Ireland; 4 CHaRT, HRSU, 3Rd Floor Health Sciences Building, Foresterhill, Aberdeen, AB25 2ZD UK; 5 Faculty of Kinesiology, University of New Brunswick, 3 Bailey Drive, P.O. Box 4400, Fredericton, NB E3B 5A3 Canada; 6 NMAHP Research Unit, Glasgow Caledonian University, Govan Mbeki Building, Cowcaddens Road, G4 0BA Glasgow, UK; 7 School of Social and Political Sciences, University of Glasgow, 25-29 Bute Gardens, Glasgow, G12 8RS UK; 8 School of Health & Life Sciences, University of the West of Scotland, High Street, Paisley, Renfrewshire, PA1 2BE UK; 9 Institute for Social Marketing and Health, Pathfoot Building, University of Stirling, Stirling, FK9 4LA UK; 10 Centre for Public Health, UKCRC Centre of Excellence for Public Health Research (NI), Institute Clinical Sciences A, Grosvenor Road, Belfast, BT12 6BJ Northern Ireland; 11 Curtin School of Population Health, Curtin University, Perth, Australia; 12 Queen’s University Belfast, University Road, Belfast, BT7 1NN Northern Ireland; 13 Dundee University, Dundee, UK; 14 Men’s Health Forum, 49-51 East Rd, Hoxton, London, N1 6AH UK; 15 Bristol Medical School, University of Bristol, Canynge Hall, 39 Whatley Road, Bristol, BS8 2PS UK; 16 Health Economics Research Unit, University of Aberdeen, Polwarth Building, Foresterhill, Aberdeen, AB25 2ZD UK

**Keywords:** Randomised controlled trial, Men with obesity, Text messages, Financial incentives, Weight management, Health inequalities, Process evaluation, Cost-effectiveness

## Abstract

**Background:**

Obesity increases the risk of type 2 diabetes, heart disease, stroke, mobility problems and some cancers, and its prevalence is rising. Men engage less than women in existing weight loss interventions. Game of Stones builds on a successful feasibility study and aims to find out if automated text messages with or without endowment incentives are effective and cost-effective for weight loss at 12 months compared to a waiting list comparator arm in men with obesity.

**Methods:**

A 3-arm, parallel group, assessor-blind superiority randomised controlled trial with process evaluation will recruit 585 adult men with body mass index of 30 kg/m^2^ or more living in and around three UK centres (Belfast, Bristol, Glasgow), purposively targeting disadvantaged areas. Intervention groups: (i) automated, theory-informed text messages daily for 12 months plus endowment incentives linked to verified weight loss targets at 3, 6 and 12 months; (ii) the same text messages and weight loss assessment protocol; (iii) comparator group: 12 month waiting list, then text messages for 3 months. The primary outcome is percentage weight change at 12 months from baseline. Secondary outcomes at 12 months are as follows: quality of life, wellbeing, mental health, weight stigma, behaviours, satisfaction and confidence. Follow-up includes weight at 24 months. A health economic evaluation will measure cost-effectiveness over the trial and over modelled lifetime: including health service resource-use and quality-adjusted life years. The cost-utility analysis will report incremental cost per quality-adjusted life years gained. Participant and service provider perspectives will be explored via telephone interviews, and exploratory mixed methods process evaluation analyses will focus on mental health, multiple long-term conditions, health inequalities and implementation strategies.

**Discussion:**

The trial will report whether text messages (with and without cash incentives) can help men to lose weight over 1 year and maintain this for another year compared to a comparator group; the costs and benefits to the health service; and men’s experiences of the interventions. Process analyses with public involvement and service commissioner input will ensure that this open-source digital self-care intervention could be sustainable and scalable by a range of NHS or public services.

**Trial registration:**

ISRCTN 91974895. Registered on 14/04/2021.

**Supplementary Information:**

The online version contains supplementary material available at 10.1186/s13063-022-06504-5.

## Administrative information

Note: the numbers in curly brackets in this protocol refer to SPIRIT checklist item numbers. The order of the items has been modified to group similar items (see http://www.equator-network.org/reporting-guidelines/spirit-2013-statement-defining-standard-protocol-items-for-clinical-trials/).

**Table:** 

Title {1}	Effectiveness and cost-effectiveness of text messages with or without endowment incentives for weight management in men with obesity: The Game of Stones randomised controlled trial
Trial registration {2a and 2b}.	ISRCTN: 91974895 https://doi.org/10.1186/ISRCTN91974895 (registered 14/04/2021)
Protocol version {3}	Version 4.0 dated 27.04.2022
Funding {4}	The National Institute for Health and Care Research (UK) (Ref: NIHR 129,703)
Author details {5a}	Dr Lisa Macaulay Game of Stones Trial Manager NMAHP Research Unit, Pathfoot Building, Stirling University, Stirling, FK9 4LA Tel: + 44 (0)1786 467,491 Email: lisa.macaulay@stir.ac.uk Dr Catriona O’Dolan Game of Stones Trial Manager NMAHP Research Unit, Pathfoot Building, Stirling University, Stirling, FK9 4LA Tel: + 44 (0)1786 467,491 Email: catriona.odolan@stir.ac.uk Prof Alison Avenell Clinical Chair in Health Services Research, Health Services Research Unit, 3rd Floor Health Sciences Building, Foresterhill, Aberdeen AB25 2ZD Tel: + 44 (0)1224 438,164 Email: a.avenell@abdn.ac.uk Dr Paula Carroll Men's Health Researcher with MFHI Department Sport & Exercise Science, Waterford Institute of Technology, Main Campus Cork Road, Waterford City, Co. Waterford, Ireland Tel: + 353 51 83 4141 Email: PCARROLL@wit.ie Dr Seonaidh Cotton Senior Trials Manager, CHaRT, HRSU, 3rd Floor Health Sciences Building, Foresterhill, Aberdeen AB25 2ZD Tel: + 44 (0)1224 438,178 Email: s.c.cotton@abdn.ac.uk Assoc Prof Stephan Dombrowski Health Psychologist, Faculty of Kinesiology at the University of New Brunswick, 3 Bailey Drive, P.O. Box 4400, Fredericton, New Brunswick, Canada, E3B 5A3 Email: stephan.dombrowski@unb.ca Mr Andrew Elders Statistician, NMAHP Research Unit Govan Mbeki Building, Glasgow Caledonian University, Cowcaddens Road, Glasgow, G4 0BA Tel: + 44 (0)141 2,731,307 Email: Andrew.elders@gcu.ac.uk Dr Beatriz Goulao Trial Statistician, CHaRT, HRSU, 3rd Floor Health Sciences Building, Foresterhill, Aberdeen AB25 2ZD Tel: + 44 (0)1224 438,198 Email: Beatriz.Goulao@abdn.ac.uk Prof Cindy Gray Interdisciplinary Professor of Health and Behaviour (School of Social and Political Sciences), University of Glasgow, 25–29 Bute Gardens, Glasgow, G12 8RS Tel: + 44 (0)141 3,306,274 Email: Cindy.Gray@glasgow.ac.uk Prof Fiona M. Harris School of Health and Life Sciences University of the West of Scotland, High Street, Paisley, Renfrewshire, PA1 2BE Tel: + 44 (0)141 848 3000 Email: fiona.harris@uws.ac.uk Prof Kate Hunt Institute for Social Marketing and Health, Pathfoot Building, University of Stirling, Stirling, FK9 4LA Tel: + 44 (0)1786 466,354 Email: kate.hunt@stir.ac.uk Prof Frank Kee Director for UKCRC Centre of Excellence for Public Health Research (NI) and Deputy Director for the Centre for Public Health, Queen's University Belfast, Institute Clinical Sciences A, Grosvenor Road, Belfast, BT12 6BJ, Northern Ireland Tel: + 44 (0)28 9097 8943, + 44 (0)28 906 32,596 Email: F.Kee@qub.ac.uk Prof Graeme MacLennan Director CHaRT, 3rd Floor Health Sciences Building, Foresterhill, Aberdeen AB25 2ZD Tel: + 44 (0)1224 438,147 Email: g.maclennan@abdn.ac.uk Mr Matthew David McDonald Feasibility study fieldworker consultant Curtin School of Population Health, Curtin University, Perth, Australia Email: m.mcdonald1@postgrad.curtin.edu.au Prof Michelle McKinley (PI GoS Belfast) Professor of Nutrition Queen's University Belfast, University Road, Belfast, BT7 1NN, Northern Ireland Tel: + 44 (0)28 9097 8936, + 44 (0)28 906 32,685 Email: m.mckinley@qub.ac.uk Ms Rebecca Skinner Feasibility study fieldworker consultant Dundee University Email: RSkinner001@dundee.ac.uk Ms Claire Torrens NMAHP Research Unit, Pathfoot Building, Stirling University, Stirling, FK9 4LA Tel: + 44 (0)1786 466,341 Email: Claire.Torrens@stir.ac.uk Mr Martin Tod CEO Men’s Health Forum (GB) Men's Health Forum, 49–51 East Rd, Hoxton, London N1 6AH Tel: + 44 (0)20 79,227,908 Email: martin.tod@menshealthforum.org.uk Prof Katrina Turner (PI GoS Bristol) Professor in Primary Care Research Bristol Medical School, University of Bristol, Canynge Hall, 39 Whatley Road, Bristol, BS8 2PS Tel: + 44 (0)117 3,314,559 Email: Katrina.Turner@bristol.ac.uk Prof Marjon van der Pol Chair in Health Economics, Health Economics Research Unit, University of Aberdeen, Polwarth Building, Foresterhill, Aberdeen, AB25 2ZD Tel: + 44 (0)1224 437,172 Email: m.vanderpol@abdn.ac.uk Prof Pat Hoddinott BSc, MB BS, FRCGP, MPhil, PhD. Chair in Primary Care, NMAHP Research Unit, Pathfoot Building, Stirling University, Stirling, FK9 4LA Tel: + 44 (0)754 0,674,504 Email: p.m.hoddinott@stir.ac.uk
Name and contact information for the trial sponsor {5b}	Rachel Beaton Research Integrity & Governance Manager Research and Enterprise Office 3B1 Cottrell Building, University of Stirling FK9 4LA Tel: + 44 (0)1786 466,196 Email: rachel.beaton@stir.ac.uk
Role of sponsor {5c}	The Sponsor is not directly involved in the trial design; collection, management, analysis, and interpretation of data; or writing of the report. However, the Sponsor oversees all aspects of the trial, including reporting for publication.

## Introduction

### Background and rationale {6a}

This randomised controlled trial (RCT) will address the increasing prevalence of obesity, consequent morbidity and engaging men in interventions to achieve and maintain clinically significant weight loss, particularly in areas where there are health inequalities. Men die sooner than women and year-on-year mortality improvements have slowed, particularly in disadvantaged areas [1, 2]. Men engage less often than women in existing weight loss interventions [3], and sustainable interventions with broad reach are needed for men who do not like, or cannot access, weight management groups. Obesity increases the risk of type 2 diabetes, heart disease, stroke, mobility problems and some cancers, leading to multi-morbidity and mental health consequences. Weight loss can reduce premature all-cause mortality and reverse the early stages of type 2 diabetes; hence, reducing obesity is a UK Government priority. Recent evidence shows that obesity and health inequalities are risk factors for worse health outcomes for people with Covid-19 [4–6].

This trial will therefore be important to the public, the National Health Service (NHS) and society. In 2019, 28.0% of adults in England were living with obesity and a further 36.2% were overweight [7]. In 2019/20, 27% of adults in Northern Ireland were obese, with a further 38% overweight [8]. The Scottish Health Survey (self-reported weight over telephone) reported that 27.5% of adults were obese and a further 34.7% were overweight, with 77% of Scottish men aged 65–74 overweight or obese in 2020 [9]. In 2020/21, 26% of women and 22% of men in Wales reported being obese, with more men than women being obese or overweight [10]. Testing innovative, scalable, digital interventions for reducing obesity is recommended to increase reach to men and promote self-management [11]. These can then be offered within current National Institute for Health and Care Excellence (NICE) recommended tiered weight management services [12] to span primary, secondary and tertiary disease prevention.

Game of Stones (GoS) is a parallel, 3-arm RCT that delivers automated Short Messaging System (SMS) texts over a 1-year period, with or without financial incentives, and compares weight change at 12 months (M) from baseline with a waiting list comparator group, for men living with obesity. The GoS feasibility study was over-subscribed and demonstrated broad acceptability, and 60% of the 105 men participating lived in the two most disadvantaged Scottish Index of Multiple Deprivation (SIMD) postcode quintiles, a higher proportion than in other UK obesity trials [13–15]. Fewer men living in disadvantaged areas dropped out compared to men living in more advantaged areas. An independent Trial Steering Committee (TSC) judged that progression criteria for a full RCT had been met and this full RCT follows a similar trial protocol with some modifications to take account of the advent of Covid-19.

The research team were invited to apply for additional funding from NIHR to investigate UK policy priority areas: mental health and multiple long-term conditions within the Game of Stones Trial. A new secondary outcome, Patient Health Questionnaire – 4 (PHQ-4), was added in September 2021 and funding for this element was contracted in March 2022.

## Rationale

The research question is as follows: Are automated SMS texts, delivered to support behaviour change, with or without endowment incentives, effective and cost-effective for weight loss at 12 M compared to a waiting list comparator arm in men with obesity? Four systematic (ROMEO) reviews were conducted of the quantitative, qualitative and economic evidence for the weight management of men living with obesity, and the rationale for the SMS texts and incentive interventions in GoS was supported by this evidence [16]. This led to a promising feasibility study of GoS [14] and a systematic review of text messages for weight management [17].

The systematic review with meta-analysis of 15 RCTs examining SMS-delivered interventions for weight loss (*n* = 12) and weight loss maintenance (*n* = 3) was conducted [17]. The weight loss trials had an effect at intervention end (median duration 6 months) with a mean difference − 2.28 kg (95% confidence interval [CI] − 3.18 to − 1.36 kg). Men accounted for 41% of trial participants, higher than for other interventions [17]. There were no men-only trials and data relevant to health inequalities were not reported. The ROMEO reviews [16] suggest that interventions need to be designed together with men in the target population. A more recent systematic review concluded that socioeconomic factors were rarely considered during intervention design, conduct and reporting of men’s weight management trials [18]. In GoS, Men’s Health Forum (MHF) charities and men in the target population were involved in writing the text messages to ensure the language, content, frequency and timing were acceptable [14]. GoS SMS texts can be delivered to any mobile phone at any time or place and are scalable [19]. In 2019, 93% of men and 89% of adults in financially vulnerable households had access to a mobile phone [20]. Commercial group-based weight management interventions may be particularly unappealing to men from disadvantaged areas [21], and individual, remotely delivered interventions like GoS are potentially able to engage these men. GoS SMS texts appealed to the men participating in the feasibility study as they encouraged autonomy and were non-stigmatising and low burden [14]. The infrequent assessment visits (4 over 1 year) and minimal time commitment were also attractive to men living in disadvantaged areas [14].

Systematic review evidence of financial incentives shows their potential to change habitual behaviours and help reduce health inequalities [22]. Moreover, the evidence for financial incentives for weight loss is growing [23] and deposit contracts can be effective [24]. But deposit contracts, where people put their own money into an account, and lose it if weight loss targets are not met, may increase health inequalities for the cash poor. In GoS, incentives were designed drawing on evidence, behavioural economics, PPI input and a discrete choice experiment (DCE) with 1045 men with obesity [14]. The incentives “endow” participants with a (hypothetical) deposit thereby mimicking a deposit contract. The feasibility trial demonstrated the acceptability and promise of the incentive intervention [14]. Offering an incentive was valued by men on low incomes for a variety of reasons, such as weight loss resulting in them needing to buy new clothes. This trial is running concurrently with a UK cost of living crisis with inflation in the costs of food and fuel. A Food Foundation report in 2020 highlights that if people follow Public Health England’s healthy eating advice, those in the bottom 10% of income would need to spend 74% of their household income on food [25], further highlighting the importance of health inequalities.

This trial will provide evidence on effectiveness, cost-effectiveness and impact on health inequalities relevant for policy makers and service commissioners. Participants living with obesity and mental health conditions and/or multiple long-term conditions (MLTC) are a particular focus, as there is a gap in the evidence for weight loss interventions in populations of men with MLTCs and poor mental health. Addressing health inequalities, mental health and MLTC are key government priorities, together with understanding the mediators and moderators for weight management. These are all inter-related, and casual pathways are complex and multi-directional.

In summary, GoS will target a clear evidence gap in weight management services and health inequalities for men. Obesity, health inequalities and prevention are all UK policy priorities in the NHS Long Term Plan [26] and are likely to remain so for the foreseeable future, particularly with new evidence emerging about risk factors for Covid-19 [4–6]. The implementation focus will involve public service technology experts so that SMS texts and incentives can, in future, be delivered centrally or locally, together or separately. This self-care intervention can accommodate future technological advances in digital scales linked to databases to minimise staff resource requirements. The GoS trial will produce evidence for NHS service commissioners to show whether financial incentives with or without SMS texts can improve outcomes for men living with obesity.

### Objectives {7}

#### Primary objective

To conduct a 3-arm RCT to estimate between-group difference in % weight change at 12 M from baseline for men with obesity who receive (i) SMS plus financial Incentive (SMS + I), (ii) SMS-only and (iii) 12 M waiting list for SMS texts.

### Secondary objectives

- To assess differences between groups in secondary outcomes
- To assess the cost-effectiveness of SMS + I and SMS-only compared to a waiting list comparator
- To understand men’s and service providers’ experiences of the intervention
- To follow up men at 24 M (12 M after texts/incentives cease for the intervention groups; 9 months after the SMS texts cease for the waiting list group). Request consent for linkage to long-term health outcome data
- To refine the digital programming for future scalability and implementation
- To compare PHQ-4 [27], Warwick and Edinburgh Mental Well-being Scale (WEMWBS) [28], Quality of Life Anxiety and Depression dimension (EQ-5D-5L-AD) [29] and Weight Self-Stigma Questionnaire (WSSQ) [30] measures for the 3 trial groups at baseline and 12 M
- To undertake exploratory moderator analyses examining interactions between mental health/wellbeing status at baseline and 12 M weight change
- To undertake exploratory mixed methods analyses examining mental health/wellbeing status, weight change trajectories, health inequalities, lived experiences and views to inform implementation, tailoring and future research
- To undertake exploratory moderator analysis for weight change at 12 M by the presence/absence of MLTC (with and without diabetes) at baseline
- To compare secondary Quality of Life, mental health/wellbeing outcomes for men with or without MLTC at baseline
- To undertake exploratory mixed method analyses to understand the lived experiences of men with MLTC and what would make a difference for recruitment, implementation, tailoring and future weight management interventions
- To undertake a mixed method process evaluation to inform future implementation and research

### Trial design {8}

A pragmatic, multi-centre, parallel, 3-arm, assessor-blind, 1:1:1 superiority RCT comparing weight change at 12 M for (i) SMS + I, (ii) SMS-only and (iii) 12 M waiting list for SMS texts, with 24 M follow-up (i.e. 1 year post intervention), mixed methods process evaluation, cost-effectiveness modelling and consent for future data linkage to longer-term health outcomes.

## Methods: participants, interventions and outcomes

### Study setting {9}

Participants living in and around Belfast, Bristol and Glasgow will be recruited. These three centres cover diverse geographical areas enabling purposive selection of community and GP assessment venues to target areas of socioeconomic disadvantage, ethnic and geographic diversity (urban, suburban, town, rural).

### Eligibility criteria {10}

#### Socioeconomic position and inequalities

The aim of the trial is to have an inclusive approach with broad eligibility across all sectors of society and to address health inequalities. Recruitment will include but is not limited to men with low income, poverty, anti-social or un-predictable working hours, relationship problems, loneliness, poor mental health, mobility or access difficulties. The aim, by targeting disadvantaged areas for recruitment, is to recruit a similar proportion of men living in disadvantaged areas (60%) as in the feasibility study [14] for subgroup analysis. To date, only one trial in men has conducted an a priori subgroup analysis of weight outcomes comparing high and low socioeconomic status participants [18].

### Inclusion criteria

- Men with body mass index (BMI) equal to or greater than 30 kg/m.^2^
- Aged 18 or over, understand trial information and able to give informed consent
- Resident in and around Belfast, Bristol and Glasgow

### Exclusion criteria

- Inability to understand the trial information or the English language SMS texts
- No mobile phone access
- Planning to move out of the area within 12 M
- Current or recent (in last 6 months) participation in a research weight loss intervention study (participants from the feasibility study are welcome to participate in this RCT)
- Plan to have bariatric surgery within 12 M
  Additional exclusion criteria for GP screening prior to sending invitation letters:
- Known terminal illness or severe psychiatric illness
- Known impaired cognitive or visual function that would limit understanding of trial information and SMS texts

### Who will take informed consent? {26a}

University employed trial fieldworkers will obtain written informed consent at the enrolment visit. A trial website link to a downloadable version and an audio recording of a researcher reading out (verbatim) both the Summary and the Full Participant Information Sheets (PILs) for those with low literacy or poor vision will be provided prior to this visit to allow as much time as men require to consider participation. Confidentiality of data will be explained, and participants will be requested to not discuss which group they are allocated to with other participants in the trial or to talk about the trial on social media. This is to reduce any contamination or disappointment bias arising from participants who are not allocated to the incentive group. Participants will be informed they can choose whether to participate or not, and they can withdraw from the trial at any point if they wish, without giving a reason and without any personal consequences. Following informed consent, if a participant loses capacity, the consent given when capable will remain legally valid. In such circumstances, a decision will be made, in conjunction with the participant and any family or carers, in relation to ongoing participation in the trial.

### Additional consent provisions for collection and use of participant data and biological specimens {26b}

Consent will be sought and obtained for potential future linkage to routine NHS data and for sharing of anonymised participant-level data. No biological specimens will be collected in this trial.

## Interventions

### Explanation for the choice of comparators {6b}

The comparator arm of a waiting list for 12 M followed by 3 M of SMS texts commencing the Monday after the 12 M assessment (or closest preferred Monday) was chosen in this trial based on feasibility study findings [14]. At baseline, all participants including the comparator group will receive information (trial website) about weight loss and will be given a pedometer (iGank China). Comparator arm participants will be assessed for the same outcome measures as the other groups at 12 M and 24 M only. There will be no interim measurements (at 3 M and 6 M) in the comparator arm. Participants can choose whether to try to lose weight or not during the 12 months. No weight loss targets will be set, and comparator participants do not have access to a self-monitoring page. The comparator arm will be as close as possible to “doing nothing”, whilst remaining ethical by providing information, free choice to lose weight and later assistance (after 12 M follow-up) via SMS texts to reduce disappointment bias. This comparator group design was decided through PPI consultation and qualitative research in the feasibility study and was acceptable to most men (only one man withdrew due to allocation disappointment) [14].

### Intervention description {11a}

The interventions are described following the Template for Intervention Description and Replication (TIDieR) guidance [31], applying the principles of self-management and designed to be efficient whilst minimising burden on NHS weight management service staffing resources. The logic model and sample SMS texts are available in Supplementary Information [see Additional Files 1 and 2].

#### SMS

##### Why-theory

The SMS texts were designed in collaboration with nine men from the University of Stirling Public Engagement Group and MHF and informed by the following: evidence-based behaviour change techniques (BCTs); systematic review of effective SMS text studies [17] and analysis of qualitative data on acceptability and men’s preferences in the feasibility study [14]. The SMS texts are written in a conversational tone and clustered around weekly weight management themes. They include reflections, encouragement and tips from other men together with web links to relevant information sources. The source of texts from the perspective of participants was the GoS feasibility study [14]. They are not based on a cast of characters or a story. Further refinement of the texts was undertaken with PPI at the three trial centres during trial set-up and the editorial and creative consultant of MHF GB, who has provided award-winning health guides and online material for men on weight and other health issues [32, 33]. SMS texts delivered over a 12 M period are embedded with evidence and theory-based BCTs based on the Health Action Process Approach [34], Self-Determination Theory [35] and the Behaviour Change Maintenance Model [36]. Daily SMS texts target automatic processes by acting as a consistent prompt and cue reminding participants of their intention to lose weight, thus aiming to reduce impulsive behaviours [37]. The following feedback from men in the target population was incorporated: include concrete tips, facts and links to information websites; include a range of perspectives and approaches as no one approach will suit everyone all the time; introduce engagement elements such as questions to prompt reflections and humour. Men liked texts to read as if they were based on experiences from other men taking part.

##### Why-intervention components

The SMS texts include BCTs which target motivation, self-regulation and maintenance processes, underpinned by a person centred-approach [38]. Some SMS texts are derived from qualitative interview data from the feasibility study where men reported what they found helpful [14] and supported by a qualitative evidence synthesis of weight management in men with obesity [39]. Engagement is encouraged by asking direct and rhetorical questions on weight management and employing general communication techniques such as agenda setting or use of humour. Some texts embed links to relevant websites for more information and self-monitoring sites (e.g. weight, step count). Participants will be invited to reflect on and implement changes to their normal diet and activity in line with evidence-based recommendations [14]. The sequencing of SMS texts covers weight loss (until 6 M), followed by more weight loss maintenance topics (6 M-12 M). Individual SMS texts are non-consecutive and stand-alone: they do not require paying attention to or engaging with previous content. After each weight assessment at 3 M, 6 M and 12 M, the SMS-only and SMS + I participants receive a personalised SMS text including the participant’s name and weight change.

##### What-materials

In addition to the SMS texts, participants will be provided with a webpage (written information if no internet access) offering a choice of evidence-based weight management and physical activity approaches, plus a pedometer to operationalise the self-monitoring BCTs embedded within the SMS texts.

##### What-procedures

Single SMS texts will be sent at a default rate of one message per day.

##### Who provides

The SMS texts will be delivered by the Health Informatics Centre (HIC) at the University of Dundee via tried and tested technology used in the feasibility study and other RCTs [40–42]. The intervention package was designed so the SMS text library can be readily delivered by other SMS providers to facilitate future roll out by the NHS or public services.

##### How (mode of delivery)

This is done through remote delivery. SMS texts were sent by the HIC automated system.

##### Where

SMS texts can be received anywhere depending on access and read at any time. Participants require a mobile phone (any type, does not have to be a smart phone) to maximise reach.

##### When and how much

Daily texts will arrive at varying times between 9am and 8.30 pm with options to adjust the number of texts sent per week flexibly between zero, three, five, seven (default), based on feasibility study findings [14]. This will enable men to tailor the text frequency according to preferences for example, ill health and bereavement. Preference for am or pm can be accommodated, e.g. for men who work night shifts.

#### Intervention 2: endowment incentive adjunct to SMS texts

##### Why-theory

The novel endowment incentive for verified weight loss in addition to the SMS texts was based on theory [43, 44] and evidence [22–24, 45–47]. In the feasibility study, the incentive structure was decided by considering evidence from multiple sources: a DCE completed by 1045 men in the target population; extensive Patient and Public Involvement (PPI); systematic review evidence; psychological and economic theory and analysis of qualitative interview data [14]. The incentive draws on behavioural economics [43, 44] which shows that people ascribe more value to something because it belongs to them (endowment effect) and are more motivated to avoid losses than they are to achieve similarly sized gains (loss aversion).

##### Why-intervention components

The incentives will be offered alongside the SMS texts because systematic reviews consistently report that financial incentives alone are unlikely to be effective for sustained complex behaviour change, without additional BCTs [23, 45, 48].

##### What-materials

All participants in this arm will be “endowed” with the financial incentive at the start of the trial. Money can only be accessed at 12 M (no withdrawals) if weight loss targets are met. It will be placed into a hypothetical personal account (held by the incentive provider), and a mock-up cheque, printed on high-quality paper, will be given to participants at the start to encourage them to feel ownership of the money.

##### What-procedures

Participants will receive the full incentive at 12 M if they achieve all three weight loss targets, but they will “lose” money if targets are not met. Detail is described elsewhere [14]. The top-level weight loss target suggested by NICE is 10% [12] and this was endorsed by PPI. The full incentive can be secured by meeting all verified weight loss targets: 5% of body weight lost (from baseline) at 3 M, 10% lost at 6 M and 10% lost at 12 M. At 6 M and 12 M, some money for each % weight loss not attained between 5 and 10% will be lost. Weight must be objectively measured by a fieldworker on research calibrated scales within 3 weeks of the target date. The incentive will be calculated automatically when verified weight is entered into the centralised database. Participants then receive a personalised SMS text with the amount of incentive secured/lost and will be encouraged to keep trying as they can still secure money. Weight and incentive status will also be presented on their personal private webpage. All participants will be given a wallet sized GoS appointment card with their personalised target weights for each appointment date and fieldworkers will fill in objectively measured weight at each assessment.

##### Who provides

Incentives will be provided as part of this NIHR research grant and are triggered by achieving target weight loss, verified by the fieldworker.

##### How (mode of delivery)

Participants will receive an automatic bank transfer of money to their bank account (BACS) once the automated database confirms the amount at the 12 M assessment. Secure postal payment methods will be available if required.

##### Where

Weight goal verification will be undertaken by fieldworkers at venues convenient for the participant, usually a health or community centre. Entering participant weight onto the database will trigger an automatic SMS text notification.

##### When

At 12 M, participants will receive the money depending on weight loss achievements [14]. If weight at 12 M exceeds baseline weight, no payment will be given, regardless of whether interim weight loss targets were met.

### Criteria for discontinuing or modifying allocated interventions {11b}

Participants can reduce the SMS text frequency from one per day to three or five per week or increase it again to seven per week (maximum) at any time during the 12 M intervention. SMS texts can be stopped and restarted by a simple instruction, e.g. “STOP TEXTS” and “RESTART”. Participants who stop the SMS texts by default will remain in the trial, unless they also request to withdraw from attending follow-up appointments, being weighed, or both. The offer of an endowment incentive may be declined by participants: they will remain in the trial unless they also request to withdraw from attending follow-up appointments, being weighed, or both.

### Strategies to improve adherence to interventions {11c}

Both interventions will be automated and centrally delivered linked to the HIC recruitment tracker, so fidelity of delivery is unlikely to be an issue. Engagement with the interventions (SMS texts received from participants; GoS website access data, self-report items in questionnaire at 12 M), retention and any reasons offered for withdrawing from any aspects of the trial will be documented.

### Relevant concomitant care permitted or prohibited during the trial {11d}

Men planning to have bariatric surgery will be ineligible for the trial. Otherwise, men are free to choose whether and how to access usual NHS, Local Authority, voluntary sector or private paid for weight-loss services. Concomitant engagement with services relating to weight loss will be recorded at baseline and 12 M assessment visits.

### Provisions for post-trial care {30}

This is a low-risk public health trial, so no post-trial care was incorporated.

### Outcomes {12}

- Primary outcome at 12 M: within-participant change from baseline weight expressed as a percentage of baseline weight at 12 M from baseline. The trial is powered on a 3% weight loss, which NICE [49] states is clinically significant and consistent with STAR-LITE Core Outcome set for obesity trials [50].
- Secondary outcomes at 12 M: absolute weight change from baseline (kg); % of participants achieving 0 < 5% weight loss; ≥ 5 < 10% weight loss; ≥ 10% weight loss, % of participants losing any weight, % of participants gaining weight; EQ-5D-5L; EQ-5D-5L-AD; WEMWBS; PHQ-4; WSSQ.
- Health economic outcomes: NHS costs, quality-adjusted life years (QALYs), incremental cost per QALY gained and incremental cost per % weight loss over trial follow-up and modelled lifetime.
- Exploratory outcomes at 12 M: weight management strategies used; self-monitoring weight; self-monitoring steps; self-reported physical activity, alcohol, smoking; satisfaction with GoS; satisfaction with weight loss progress; confidence in ability to lose weight; confidence in ability to maintain weight loss long term.
- Exploratory outcomes at 24 M: weight change (absolute kg, %) from baseline and from 12 M. Depending on the advice of the TSC, additional outcomes may be reported (Table 1).

- Process outcomes: incentives gained; weight change (absolute, %) at 3 M and 6 M; number and chosen frequency of SMS texts delivered; any responses to SMS texts; web page use; recruitment and retention by recruitment strategy (i.e. community v GP), health inequalities: perceived wealth [51]; financial strain [52, 53] to explore the UK cost of living crisis unfolding during this trial.
- Qualitative sub-study: telephone interview data with a diverse and purposive sample of participants at 12 M and/or 24 M will generate findings which will help to understand experiences and behaviours of men during and after the interventions and barriers/facilitators to longer-term sustained weight loss and scalability of the intervention.

The outcome assessment schedule is shown in Table 1.

**Table 1:** Table of endpoints/outcomes

*Data collection*	^a^0 M	12 M	^b^24 M
Socio-demographic: IMD, co-morbidities (physical and mental health), self-report disability, ethnicity, age, education, employment, household size; relationship status	✓		
Perceived wealth, financial strain	✓	✓	✓
Anthropometry—height (for BMI)	✓		
Anthropometry—weight	✓	✓	✓
Participant satisfaction		✓	✓
Health behaviours—physical activity, smoking status, alcohol frequency	✓	✓	
Weight management strategies used over last 12 months	✓	✓	✓
Confidence in ability to lose weight and maintain weight loss	✓	✓	✓
PHQ-4	✓	✓	
EQ-5D-5L – Anxiety and Depression Dimension (AD)	✓	✓	
Warwick-Edinburgh Mental Well-being Scale (WEMWBS)	✓	✓	
Weight Self-Stigma Questionnaire (WSSQ)	✓	✓	
Health Economic Outcomes: EQ-5D-5L, NHS health care use	✓	✓	✓
Qualitative interview data (experiences, behaviours)		✓	✓
Process outcomes: observed weight meets target and incentives secured/lost at 3 M and 6 M; number of SMS texts delivered; SMS texts responses, web page use over 12 M	✓	✓	
Unintended consequences or adverse events		✓	✓

^a^0 M = Baseline, i.e. pre-randomisation

^b^guided by Trial Steering Committee

### Participant timeline {13}

**Fig. 1:**
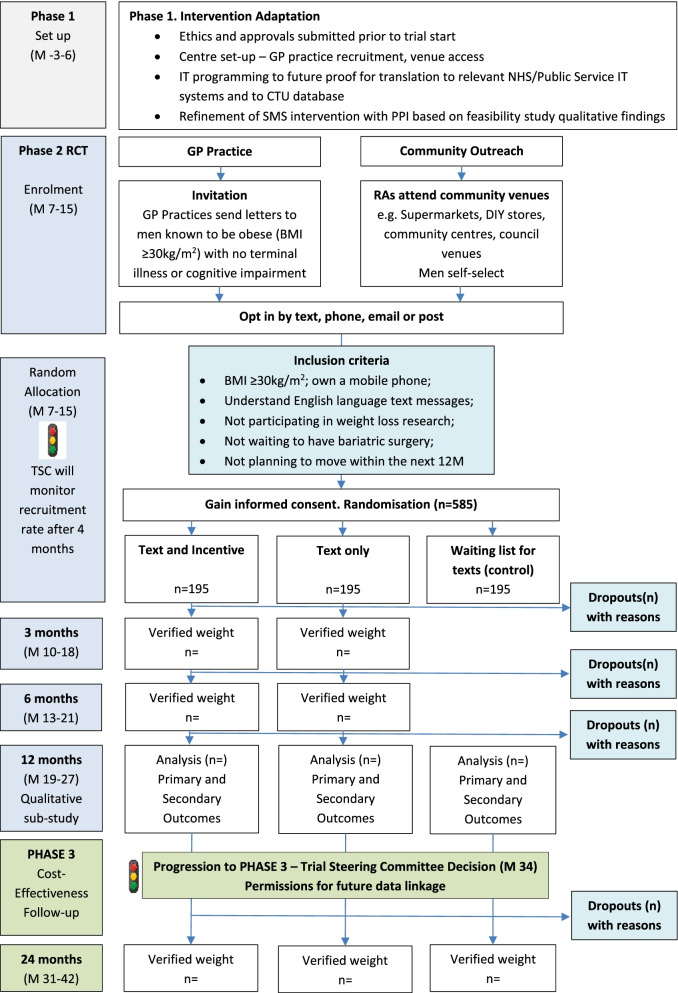
Trial flowchart

Figure 1 shows the trial flowchart, including the participant timeline.

### Sample size {14}

In the trial, 585 men with obesity will be recruited through GP obesity registers and community outreach across the three geographical areas linked to the trial centres.

#### Sample size calculation

In total, 146 men will be required in each group to detect differences in weight loss between groups of at least 3% at 12 M, with 90% power and two-sided alpha equal to 2.5% (to maintain a nominal significance level of 5% with two tests being used). With an expected 25% loss to follow-up as observed in the feasibility study at 1 year, a total of 585 men will need to be randomised: 195 per trial group and approximately 195 (65 per trial group) at each of the three centres [14]. The sample size calculation was based on detecting a mean difference in weight between intervention groups and comparator of at least 3.3 kg, assuming a pooled standard deviation of 8 kg. A minimum clinically important weight loss of 3% is recommended by NICE [49], and the mean difference of 3.3 kg is derived from 3% of 109 kg (the mean baseline weight in the feasibility study). Several trials of SMS-delivered weight management interventions [17] reported an effect size > 3.3 kg, including the largest study (*n* = 710) [54], which was the only trial to include predominantly men (82%), suggesting 3.3 kg is an achievable mean weight loss. The standard deviation for absolute weight loss ranged from 4.9 to 6.3 kg in the feasibility study (at 12 M) [14] and from 2.5 to 7.3 kg in the systematic review [17]. Therefore, a standard deviation of 8 kg was conservatively assumed for the GoS trial.

### Recruitment {15}

**Fig. 2:**
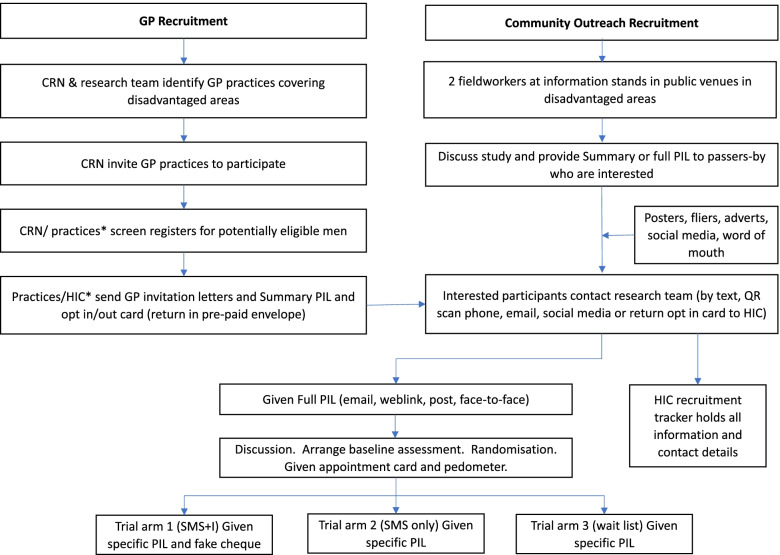
Recruitment flowchart

Initial approach and distribution of trial information will be via a range of community (e.g. supermarkets, leisure centres, DIY stores) and GP practice venues which are purposively selected for socioeconomic disadvantage and geographic diversity (urban, suburban, town, rural). GP practice staff may systematically send trial information to potentially eligible men identified through computer searches of medical records and/or signpost men to the trial website. Enrolment will be conducted in person by fieldworkers at the participant’s choice of venue. Recruitment will be flexible, including weekends and evenings, enabling men who work or have other daytime commitments to join the trial. A recruitment flowchart is shown in Fig. 2 and was based on the successful feasibility processes [15].

## Assignment of interventions: allocation

### Sequence generation {16a}

Randomisation will be stratified by trial centre using random permuted blocks, generated by computer. Randomly allocated blocked sizes of 3, 6 and 9 were used.

### Concealment mechanism {16b}

After receiving written consent, fieldworkers will randomise men using a secure remote web-based system provided by the Centre for Healthcare Randomised Trials (CHaRT). The CHaRT randomisation service will implement the allocation sequence and will be independent of the data management and statistical team who will be undertaking the outcome data analysis. Participants and the fieldworkers will be informed automatically by SMS text about group allocation.

### Implementation {16c}

CHaRT will generate the allocation sequence for intervention assignment. The randomisation service will be embedded within the trial website. Telephone randomisation will be available as a back-up in case of internet problems. The fieldworker at the recruitment site will enrol participants.

## Assignment of interventions: blinding

### Who will be blinded {17a}

Primary outcome assessment will be blinded to group allocation and undertaken by an assistant fieldworker who has not previously met the man and/or is not aware of their trial group allocation. This was tested in the feasibility study and found to be acceptable, understandable to participants and logistically feasible for implementation [14]. The GP practices or other stakeholders who facilitate recruitment (e.g. community centre staff) will not be informed of group allocation. The statistical team analysing data will be blinded to group allocation and will not have access to data collected at 3 M and 6 M outcome assessments in the intervention groups until the primary and secondary outcome analysis is complete.

### Procedure for unblinding if needed {17b}

Lead fieldworkers, Trial Managers and participants will not be blinded to group allocation. There may be circumstances when assistant fieldworkers taking weight measurements will be unblinded and this will be recorded on the CRF.

## Data collection and management

### Plans for assessment and collection of outcomes {18a}

The schedule of assessments is summarised in Table 1. Participants will be sent a reminder of their appointment by text (or preferred contact method) the day before. Non-attenders will receive two reminders to contact the team and given the opportunity to arrange another appointment. Reminders will be sent using different methods, e.g. SMS text, email, phone, post, because mobile phone numbers may change, men may run out of phone credit and some men may unpredictably work away from home. Flexibility of approach is both valued by participants and required in any study aiming to address health inequalities. Relevant information about preferred methods of contact will be documented by fieldworkers on the HIC recruitment tracker. Fieldworkers will measure height and weight using standardised procedures developed and written for the study and following device instruction manuals within a 3-week window of their personal weight target date (in order for the SMS + I group participants to qualify for the linked incentive at 3 M, 6 M and 12 M). Height will be measured using a portable standing stadiometer (Marsden HM-250P, Rotherham, UK) to the nearest 0.1 cm. Weight will be measured on mobile flat scales with double display (Seca 878, Birmingham, UK) to the nearest 0.01 kg. If the surface is uneven, a board will be placed under the scales. Weight will be entered manually onto the Case Report Form (CRF) which participants will be asked to sign to confirm the accuracy of the entered weight.

In the event of a face-to-face verified assessment not being possible for the primary outcome assessment at 12 M (e.g. participant out of the country), every effort will be made to gain a satisfactory verified weight via video assessment, or via an independently confirmed weight by a third party, e.g. a pharmacist, community or health worker. Methods will be documented, to enable a sensitivity analysis to be undertaken.

Self-report questionnaires (participant choice, online via a secure password-protected research tablet, paper, telephone if visual impairment or literacy issues), completed when attending weight assessments, will be uploaded to CHaRT databases. The questionnaires will be completed by the participants with fieldworkers present in case they require help, e.g. visual or literacy issues. Assessments use validated tools where available and follow quality standards for measurement procedures.

### Plans to promote participant retention and complete follow-up {18b}

To maximise completeness of data, participants who do not attend assessments within the 3-week time window permitted, but who have not withdrawn from the trial, may be contacted (by their preferred method) and requested to complete a questionnaire (paper copy or via email link) and provide a self-reported weight. This will not qualify for an incentive payment. A letter will be sent to those who do not attend the 12 M assessment reminding them that they will be invited to attend an appointment at 24 M. All participants will receive a £20 retail voucher as reimbursement of expenses for attending the 12 M and 24 M assessments, consistent with evidence that financial reimbursement can improve retention [55, 56]. This trial will also host a nested study within a trial (SWAT) RCT [57]. This will investigate the effect of participant-trial staff relationships on retention up to the time of the primary outcome assessment. Retention is the primary outcome for the SWAT study. Participants randomised to the intervention groups (SMS + I and SMS-only) of the main trial will immediately be randomised again (using same randomisation as described above) to receive one of two protocolised weight assessment approaches (task-oriented group or relational group) using random permuted blocks.

### Data management {19}

#### Data collection tools and source document identification

The hard copy of weight measurement documented on the CRF will be the source data and weight documentation will be agreed by participant signature. If questionnaires are completed electronically, the electronic record will be considered to be source data. If a hard copy of the CRF or questionnaire is completed, these will be considered the source document. For all other data collected, if these are completed electronically, the electronic record will be considered the source data.

#### Data handling and record keeping

The electronic data capture system (eCRF) is validated, maintains a full audit trail of data changes, is secure (requiring unique usernames and passwords) and has regular back-up. The system safeguards the blinding of trial data. Participants will have a unique participant identification number that allows identification of all data reported for each participant. Data will be entered into the database by fieldworkers working at each centre. Questionnaires may be completed by participants directly into the trial database. If they are completed as a hard copy, data will be entered into the database by the designated team members working at each centre. Staff in the trial office will work closely with fieldworkers to ensure that the data are as complete and accurate as possible. Extensive range and consistency checks will be performed to further enhance the quality of the data.

#### Access to data

Direct access will be granted to authorised representatives from the Sponsor, host institution and the regulatory authorities to permit trial-related monitoring, audits and inspections, in line with participant consent. Any hard copy data will be stored at University of Stirling and requests to access data will be administered through the University’s data archive, DataSTORRE. The investigator site files will be archived at each centre. Following publication of the results, requests for an anonymised participant-level dataset and statistical code for generating the results should be made to chart@abdn.ac.uk.

#### Archiving

Archiving will be authorised by the Sponsor following submission of the end of trial report. All trial documentation will be kept for at least 10 years after publication of trial data.

#### Monitoring, audit and inspection

The trial will be monitored by the Trial Managers and Senior Trial Manager to ensure it is being conducted as per protocol, adhering to the UK Policy Framework for Health and Social Care Research, the principles of GCP, and all other appropriate regulations. The approach to, and extent of, monitoring (specifying both central and on-site monitoring) is specified in the trial monitoring plan.

### Confidentiality {27}

Patient confidentiality will be maintained for all collected data. All investigators and fieldworkers will be trained in Good Clinical Practice (GCP) and comply with the requirements of the General Data Protection Regulation (GDPR) (or subsequent legislation), with regard to the collection, storage, processing and disclosure of personal information and will uphold the GDPR core principles. Computers are used to collate the data, and the trial website will have limited access measures via usernames and passwords. Staff at trial centres will only have access to participant data for participants at their centre. Within the trial website, identifiable data will be stored with a strong encryption algorithm. Participants will be identified using a unique participant ID number. Participant data, contact information and responses to text messages will be managed electronically in the Participant Tracker Software at HIC, which is an approved NHS safe haven for data. Data handling and storage will comply with GDPR legislation. Researchers employed at the three trial centres (Universities of Stirling and Bristol, and Queen’s University Belfast) will have secure passwords to access the HIC Tracker. Data from qualitative interviews will be anonymised before analysis by removing any information which could potentially identify the participant. Only interview transcribers who are approved by the University of Stirling and meet confidential data handling requirements will be used. The interview recordings, transcriptions and NVivo database will be password protected and encrypted and stored securely at the University of Stirling. The recorder will be wiped clean as soon as the recording has been stored. Personal data and audio recordings will be destroyed as soon as it is certain that they are no longer required (i.e. at the end of the trial/follow-up period).

### Plans for collection, laboratory evaluation and storage of biological specimens for genetic or molecular analysis in this trial/future use {33}

This trial will not involve biological specimens.

## Statistical methods

### Statistical methods for primary and secondary outcomes {20a}

#### Outcome measures

Outcomes will be measured at 3 M and 6 M (two active intervention groups), 12 M and 24 M (all three groups), unless otherwise indicated. Details on the 24 M outcome measures and analysis will be available in a separate analysis plan. Outcomes will also be measured at baseline, where applicable.

#### Framework

Primary and secondary outcomes will be compared using a superiority framework for the following intervention groups:

- SMS + endowment incentive (SMS + I) vs 12 M waiting list for SMS texts
- SMS-only vs 12 M waiting list for SMS texts

#### Primary outcome

The primary outcome will be analysed using a linear regression model adjusted for centre using a fixed effect. The trial is powered on a 3% weight loss, which NICE PH53 considers the minimum required for health benefits for lifestyle weight management programmes. This is consistent with STAR-LITE Core Outcome set for obesity trials [50] and with effectiveness of other SMS text trials for weight loss [17].

#### Secondary outcomes

Secondary outcomes will be analysed using appropriate regression models adjusted for centre and baseline variables where applicable. The statistical analysis plan (SAP) will be uploaded in Supplementary Information and will provide more detailed information about the analysis approach for each outcome. A separate health economic analysis plan and process evaluation analysis plan will be available on request. The primary economic outcome will be incremental cost per QALY using an NHS perspective. Cost-effectiveness will be assessed over the trial period and a decision model will be used to assess economic value over an extrapolated lifetime horizon. A cost-effectiveness analysis (incremental cost per % weight loss) will also be performed. Firstly, these analyses will be performed using the 12 M follow-up data, then updated if the trial steering committee recommend that the study progresses to full analysis of 24 M follow-up data (Table 1).

### Interim analyses {21b}

There will be no interim analyses. The trial recruitment progress will be monitored monthly by the Funder.

### Methods for additional analyses (e.g. subgroup analyses) {20b}

Subgroup analyses will be pre-specified and divided into confirmatory and exploratory. The confirmatory subgroup analyses will be based on hypothesised directions of effect modification of the interventions informed by the weight-loss literature [58]. Weight loss and/or weight loss maintenance are part of disease management for many obesity-related co-morbidities, e.g. diabetes, cardio-vascular disease. Confirmatory subgroup analyses include presence of obesity-related co-morbidities (at least one versus none), and self-reported diabetes at baseline versus no diabetes at baseline. The exploratory subgroup analyses will be based on potential moderators, for which there are gaps or conflicting evidence in the literature. More details on pre-specified subgroups are provided in the SAP which will be uploaded in Supplementary Information.

### Methods in analysis to handle protocol non-adherence and any statistical methods to handle missing data {20c}

#### Adherence

Analysis to account for non-compliance is not necessary for this self-care intervention, as automated interventions can only be accessed via randomisation, therefore cross-over cannot occur and contamination was minimal in the feasibility study. However, non-adherence to the weight measurement established protocol is possible. If weight data are collected that do not adhere to protocol, they will be included in a sensitivity analysis.

#### Missing data

The primary analysis will use multiple imputation as the strategy to handle missing data applying predictive mean matching [59]. Sensitivity analyses of the primary outcome will examine data under various assumptions over missingness, including:

- An analysis of observed cases only,
- Missing weight data being treated as Baseline Observation Carried Forward (BOCF) and Last Observation Carried Forward (LOCF) as recommended in the STAR-LITE core outcome set [50], for comparability with previous weight loss studies [60].

For models assessing the effectiveness of the interventions, when missing baseline outcome data is presented, mean imputation (for continuous variables) or the creation of a missing category (for categorical variables), will be implemented following best practice [61].

Data quality assurance and source data verification will be carried out in accordance with the CHaRT Clinical Trial Unit’s (CTU) standard operating procedures to minimise spurious data. Further data quality checks will be carried out by the trial statistician prior to the analysis and potentially implausible data will be queried with trial office and/or site staff. If a data item includes the option “Prefer not to say”, any such responses will be treated as a separate category and not classed as missing data.

### Plans to give access to the full protocol, participant-level data and statistical code {31c}

The full protocol is available as Supplementary Information. Following publication of the results, requests for an anonymised participant-level dataset and statistical code for generating the results should be made to chart@abdn.ac.uk.

## Oversight and monitoring

### Composition of the coordinating centre and trial steering committee {5d}

#### Trial Steering Committee (TSC)

The independent TSC will meet at least annually face to face or online, as required, to oversee all aspects of the trial, with accountability to the Funder and the Sponsor. As this trial involves a low-risk self-management intervention, the TSC will also fulfil the role of monitoring safety and make recommendations as to any modifications that are required to be made to the protocol or the termination of all or part of the trial.

#### Project Management Group

The Project Management Group (PMG) will consist of the grant holders and key trial staff (Trial Managers, research fellows, statisticians, programmers), meeting every 2–4 months remotely as required. The PMG will ensure all practical details of the trial are progressing well.

#### Trial Coordinating Team

The Trial Coordinating Team will be made up of the CI, Trial Managers and Qualitative Research Fellow and will meet regularly to oversee the day-to-day management of trial activities at all three centres. Any issues with trial management will be discussed at PMG meetings if further input is required.

### Composition of the data monitoring committee, its role and reporting structure {21a}

This is an automated self-care intervention, and there are unlikely to be any safety concerns (there were none in the feasibility study [14]. Given the low-risk nature of the intervention, it was agreed with the Sponsor and the Funder that an independent data monitoring committee would not be required.

### Adverse event reporting and harms {22}

Adverse events (AEs) and serious adverse events (SAEs) will be recorded by fieldworkers from the time a participant consents to join the trial until the end of the 24 M follow-up period. In addition, the research team may be alerted to a possible AE/SAE via screening of text message responses. The CI (or delegate) will review the AEs and make an evaluation of seriousness, causality and expectedness. If the event is confirmed by the CI as being serious, related and unexpected, the Sponsor will be notified and asked to provide an assessment of the SAE, and the Research Ethics Committee (REC) notified. All related SAEs will be summarised and reported to the REC, the Funder and the TSC in their regular progress reports.

### Frequency and plans for auditing trial conduct {23}

Investigators and their host institutions will be required to permit direct trial-related audits to take place by the Sponsor and/or regulatory representatives providing direct access to source data and documents as requested.

### Plans for communicating important protocol amendments to relevant parties (e.g. trial participants, ethical committees) {25}

Substantial and non-substantial amendments will be discussed with the PMG, and when appropriate with the independent TSC. Amendments submitted to the approving REC will be communicated to the participating centres (R&D office and local research team) to assess whether the amendment affects the NHS permission for that centre and to the Funder. The CI will be responsible for the decision to amend the protocol and ensure that substantive changes are communicated to relevant stakeholders (e.g. REC, trial registries, R&D, regulatory agencies, Funder). The amendment history will be documented in the protocol to enable the most recent protocol version to be identified. A current, up to date version of the protocol will be provided to all centres, relevant members of the trial team and members of the PMG and TSC.

### Dissemination plans {31a}

On completion of the trial, data will be analysed and tabulated in a full trial report, which will be made publicly available via the NIHR Journals Library. The results will be submitted to peer-reviewed journals and presented at national and international conferences, e.g. UK, European and International Congresses on Obesity, NHS Confederation, Royal College of General Practitioners (RCGP), Public Health, UK Society for Behavioural Medicine.

The findings will be available on the trial website, and participants who express a wish to be informed of the outcome of the trial will be notified by their chosen method (SMS text, email, post). The dissemination and engagement strategy will be guided by MHF, Men’s Sheds Association and NIHR dissemination guidance. Patients, the NHS and wider public will be engaged via a lay summary, YouTube video and social media (Twitter; Facebook).

GoS will be a new open-source digital intervention freely available to the NHS (primary or secondary care; local, regional or national); local authorities and not for profit public services, e.g. charities. The trial dataset, with the potential addition of long-term data linkage to health outcomes, could inform government policy. Outputs will enter the health care system by writing briefing papers for Government (e.g. Department of Health and Social Care; NHS Health Scotland) and Public Health Umbrella Organisations for the devolved countries (e.g. Public Health England). NICE and the Scottish Intercollegiate Guidelines Network (SIGN) will receive the findings to inform relevant obesity guidance.

## Discussion

A number of issues have arisen due to the ongoing Covid-19 pandemic:

- The burden of the pandemic on stakeholders and regulatory bodies caused delays in obtaining approvals; data processing agreements and commencing GP recruitment. GP practices and corresponding Clinical Research Networks (CRNs) prioritised Covid-19 related work and research, with the CRN in Northern Ireland suspending all non-Covid related research for the duration of recruitment. This led to the Belfast centre being reliant on community recruitment only
- Government restrictions and guidelines relating to the pandemic caused issues with recruitment such as cancelled appointments due to isolation, booking venues, use of public transport to assessment visits—with anxieties about safety expressed in PPI consultation, and requirement for Personal Protective Equipment (PPE). Government pandemic guidance relevant to the recruiting centres is followed at all times to minimise risk for participants and researchers
- The trial team have held trial management meetings online

Following invited applications for add-on funding from NIHR to investigate men living with mental health and/or multiple long-term conditions as well as with obesity, a new secondary outcome—PHQ-4—was added to the baseline and 12 M questionnaires and ethically approved in September 2021. The add-on funding was awarded in January 2022 with variation to contracts being completed in March 2022.

## Trial status

The current protocol is version 4.0 (27/04/2022). Amendments to the protocol were required in order to incorporate changes to recruitment procedures due to Covid-19 and more recently, to include changes resulting from the NIHR add-on funding application. Recruitment commenced in July 2021 and was completed in May 2022, with participant follow-up continuing until July 2023.

## Supplementary Information

**Additional file 1.** Logic model of the Game of Stones intervention(pdf): The logic model for Game of Stones SMS text intervention.

**Additional file 2.** GoS Sample SMS texts (pdf): A sample of the SMStexts used in Game of Stones.

**Additional file 3.** GoS Protocol v4.0_27.04.22R0 (pdf): Effectiveness and cost-effectiveness of text message and endowment incentives for weight management in men with obesity: The Game of Stones randomised controlled trial.

**Additional file 4.** GoS Full Participant Information Leafletv2.0_29.09.21 (pdf): The full participant information leaflet for Game ofStones.

## Data Availability

Access to the datasets will be limited to the CI and appropriate members of the trial team to permit analysis.

## Abbreviations

AE: Adverse event
BCT: Behavioural change technique
BMI: Body mass index
CHaRT: Centre for Healthcare Randomised Trials (Aberdeen)
CI: Chief Investigator
CRF: Case Report Form
CRN: Clinical Research Network
CTU: Clinical trials unit
DCE: Discrete choice experiment
EQ5D-5L: EuroQol Group questionnaire for health-related quality of life states in adults
EQ5D-5L-AD: EQ5D-5L Anxiety and Depression dimension
FFIT: Football Fans in Training
GCP: Good Clinical Practice
HIC: Health Informatics Centre (Dundee)
I: Incentive
IMD: Index of Multiple Deprivation
ISRCTN: International Standard Randomised Controlled Trials Number
M: Months
MHF: Men’s Health Forum for the devolved nations
MHFI: Men’s Health Forum Ireland
MLTC: Multiple long-term conditions
NICE: National Institute for Health and Care Excellence
NIHR: National Institute for Health Research
NMAHP: Nursing, Midwifery & Allied Health Professions
PHQ-4: Patient Health Questionnaire-4 questions relating to anxiety and depression
PIL: Participant Information Leaflet
PMG: Project Management Group
PPE: Personal and Protective Equipment
PPI: Patient & Public Involvement
QALY: Quality-adjusted life year
RCT: Randomised control trial
REC: Research Ethics Committee
SAE: Serious adverse event
SAP: Statistical analysis plan
SIGN: Scottish Intercollegiate Guidelines Network
SIMD: Scottish Index of Multiple Deprivation
SMS: Short Messaging System
SSI: Site Specific Information
SWAT: Study within a trial
TIDieR: Template for Intervention Description and Replication
TSC: Trial Steering Committee
WEMWBS: Warwick-Edinburgh Mental Well-being Scale
WSSQ: Weight Self-Stigma Questionnaire
