# Supplementary material for: Effectiveness and cost-effectiveness of text messages with or without endowment incentives for weight management in men with obesity (Game of Stones): study protocol for a randomised controlled trial
Source: Trials. 2022 Jul 22;23:582. doi: 10.1186/s13063-022-06504-5 (PMC9306253; doi:10.1186/s13063-022-06504-5)

Logic model of the Game of Stones intervention

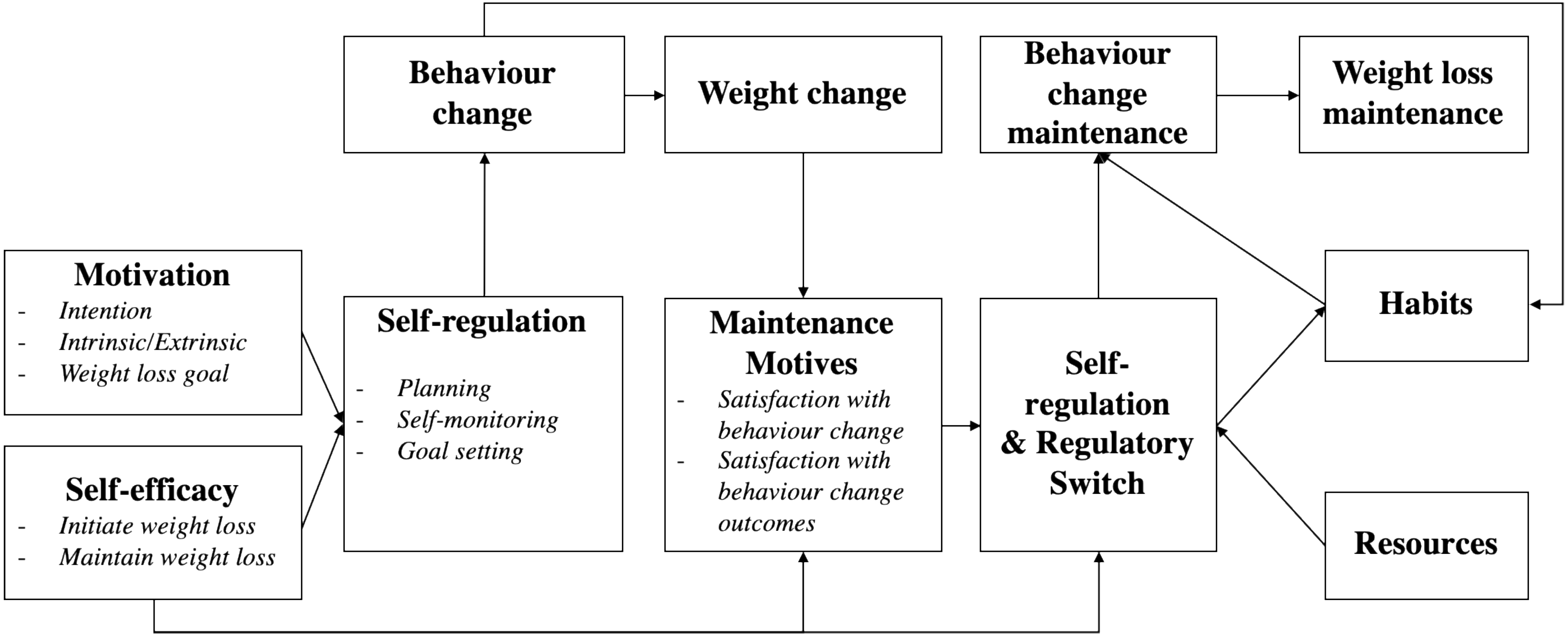

Supplement: Supplementary file 1 — Additional file 1. Logic model of the Game of Stones intervention(pdf): The logic model for Game of Stones SMS text intervention. [file 13063_2022_6504_MOESM1_ESM.pdf]
