# Supplementary material for: Effectiveness and cost-effectiveness of text messages with or without endowment incentives for weight management in men with obesity (Game of Stones): study protocol for a randomised controlled trial
Source: Trials. 2022 Jul 22;23:582. doi: 10.1186/s13063-022-06504-5 (PMC9306253; doi:10.1186/s13063-022-06504-5)
Supplement: Supplementary file 2 — Additional file 2. GoS Sample SMS texts (pdf): A sample of the SMStexts used in Game of Stones. [file 13063_2022_6504_MOESM2_ESM.pdf]

Game of Stones Trial

Sample SMS texts for Baseline, 3 months, 6 months and 9 months:

Table 1. Sample Baseline SMS texts

#	SMS	BCT	Function	Logic model
1	Welcome to Game of Stones <name>! Over 12 months we'll send you some texts. The bad news: reading texts won't change your weight. The good news: they're low calorie. We've put these texts together with the help of men who successfully lost weight. They are based on facts and real life experience.		General communication technique	
2	The goal is to lose 5% in 12 weeks. John said the goal gave him confidence to try changing his eating knowing that he's got something to work towards. Losing more than 3% of your body weight has health benefits and helps to cut your chances of cancer, heart disease and diabetes.	1.3 Goal setting (outcome)<br>5.1. Information about health consequences	Motivation	Motivation (WL goal)
3	Ok, here's the deal. Some texts will be useful and others maybe won't. Just pick whatever works for you and ignore the rest. If you want to reply to any of the texts please do. We read every text but usually we won't be able to write back. Sorry.		General communication technique	
4	How to lose weight? Here's a simple fact: sensible eating works… but only if you stick to it. Go to our Game of Stones webpage with links to webpages full of information that our men find useful. www.gameofstonesresearch.com	15.1. Verbal persuasion about capability	Motivation,<br>Engagement	Self-efficacy (initiate WL)

5	Most of our men make up their own sensible eating rules. Carl said that it's straightforward, eat less rubbish. Everybody knows it, but hard to do. Cutting down and reducing portion sizes is the main strategy that works for our men. Losing weight is about finding what works for you and your life.	4.1. Instruction on how to perform the behaviour	Self-regulation	Self-regulation (planning)
6	You're changing what you're eating and drinking, you're not dieting. Think about it as improving your lifestyle, not being on a diet. Check out this helpful wee booklet full of tips from the Men's Health Forum. www.issuu.com/menshealthforum/docs/eat_well_final_hrnon-border	4.3. Re-attribution	Motivation, Engagement	Motivation (intention)
7	Have you made any changes this week <name>? Cut down on something or made some sensible eating changes? Let us know. Have a nice rest of the weekend.	2.3. Self-monitoring of behaviour	Self-regulation, Engagement	Self-regulation (self-monitoring)

Table 2. 3 months sample SMS texts

#	SMS	BCT	Function	Logic model
1	How's it going <name>? Let's talk special occasions: birthdays, weddings, and holidays. Some men tell us that they do well with their weight loss, then a special occasion comes along and things fall down.		General communication technique	
2	Carl was out for his birthday party, eating and drinking too much, so it caused him to fall back again and he's not got back into it yet. Rob says his holidays probably set him back a lot, he went a bit over the top. Do you have any words of wisdom for the Carls and Robs in this world?	13.1. Identification of self as role model	Engagement	Self-regulation (planning)
3	You should continue to have a good time on birthdays, weddings and holidays. But without feeling guilty. How can you do that?	1.2 Problem solving	Self-regulation	Self-regulation (planning)
4	Rick says that when he's got a special occasion coming up he prepares for it and makes sure he has a period where you are doing well before making loads of sensible eating choices – this way you will have earned it even more.	1.2 Problem solving<br>6.1 Demonstration of the behaviour	Self-regulation	Self-regulation (planning)
5	Garry's golden rule is to make the first drink a soft one. He says it's easy to get carried away with the drinks and they are quite calorific. He also has regular water breaks – you can enjoy an occasion without going completely overboard.	1.2 Problem solving<br>6.1 Demonstration of the behaviour	Self-regulation	Self-regulation (planning)

6	Another way to handle your special occasion after they happened is to make up for it. Have a period after your birthday or holiday when you put effort into a sensible eating and drinking routine.	4.1. Instruction on how to perform the behaviour	Self-regulation	Self-regulation (planning)
7	It’s all about balance. If your new lifestyle ruins the fun then why stick to it? If your old routines ruin your hard work, then why bother? Hope the weekend is going well…		General communication technique	

Table 3. 6 months sample SMS texts

#	SMS	BCT	Function	Logic model
1	That's the 6 months mark! Two things are important now: Keep off any weight lost, and have your 1 year goal in mind. How confident are you that you can manage this?		General communication technique,<br>Motivation,<br>Maintenance	Self-efficacy (Maintenance),<br>Maintenance mindset
2	To keeping weight off Karl says he just walks loads now, cause he can do, anytime, anywhere. And he actually likes it and looks forward to get moving every day.	6.1 Demonstration of the behaviour	Maintenance	Maintenance mindset
3	Dave says that he's no youngster, he's heard this kinda stuff before. But he still finds it useful to remember what you know. Make sure you actually do it, not just know it.		General communication technique	
4	What are your eating plans for tomorrow? Keep staying ahead of the game.	1.4 Action planning	Engagement,<br>Self-regulation	Self-regulation (planning)
5	Gavin says his weight stays roughly the same, but he still tries new stuff. Just because you want your weight to stay the same, doesn't mean that you have to do the same things all the time.	13.2. Framing/reframing	Maintenance	Maintenance mindset

6	Jim likes to remember IF and THEN. What is your main obstacle to sensible eating? IF you run into it, THEN what will you do?	1.2 Problem solving<br>1.4 Action planning	Engagement<br>Self-regulation	Self-regulation (planning)
7	What's been your success this week? We take anything, the smaller and more unusual the better!	2.1. Monitoring of behaviour by others without feedback	Engagement<br>Self-regulation	

Table 4. 9 month sample SMS texts

#	SMS	BCT	Function	Logic model
1	Are your habits and routines working for you? Does your auto pilot work for or against you at the moment?		Engagement, Maintenance	Habits
2	Dave says that some folk need the arm round the shoulder, some folk need the boot up the backside. Do you have enough arms and boots around you when needed?	3.1. Social support	Engagement, Motivation, Self-regulation, Maintenance	Motivation, Self-efficacy, Self-regulation
3	We covered a lot of things that help with weight loss and keeping it off. They work best when used… ok, you're right, they only work when used. Which are the ones you will keep using and when?	1.4 Action planning	Engagement, General communication technique, Maintenance	Self-regulation, Maintenance mindset
4	Once every couple of weeks Carl sits down and scrolls through the messages, good reminder and some things you miss when you just read them quickly on the go.	6.1 Demonstration of the behaviour	Adjuvant behaviours, Motivation, Self-regulation, Maintenance	Self-regulation
5	Weekend coming up, finally! Remember Gavin's tip: the power of meal prep. Helps you stay ahead. Time for another batch prep session?	1.2 Problem solving	Adjuvant behaviours, Self-regulation	Self-regulation (planning)

6	Think back to 9 months ago <name>? How happy are you with the things you have achieved?	1.7 Review outcome goal(s), 15.3. Focus on past success	Engagement,<br><br>Maintenance	Maintenance Motives
7	Tim says he feels like a different person now after 9 months. He cares about food, loves his walking. A year ago, he was a totally different Tim.	13.4. Valued self-identify	Maintenance	Maintenance Motives, Maintenance mindset
