# Supplementary material for: Effectiveness and cost-effectiveness of text messages with or without endowment incentives for weight management in men with obesity (Game of Stones): study protocol for a randomised controlled trial
Source: Trials. 2022 Jul 22;23:582. doi: 10.1186/s13063-022-06504-5 (PMC9306253; doi:10.1186/s13063-022-06504-5)
Supplement: Supplementary file 3 — Additional file 3. GoS Protocol v4.0_27.04.22R0 (pdf): Effectiveness and cost-effectiveness of text message and endowment incentives for weight management in men with obesity: The Game of Stones randomised controlled trial. [file 13063_2022_6504_MOESM3_ESM.pdf]

NIHR 129703

IRAS: 290955

Effectiveness and cost-effectiveness of text message and endowment incentives for weight management in men with obesity: The Game of Stones randomised controlled trial

'The Game of Stones Trial'

PROTOCOL

This study/project is funded by the National Institute for Health Research (NIHR) Public Health Research Programme (NIHR 129703). The views expressed are those of the author(s) and not necessarily those of the NIHR or the Department of Health and Social Care.

GoS Protocol v4.0 27.04.22

**Chief Investigator (CI)**	Prof. Pat Hoddinott BSc, MB BS, FRCGP, MPhil, PhD.<br>Chair in Primary Care, NMAHP Research Unit, Pathfoot Building, Stirling University, Stirling, FK9 4LA Tel: 07540674504<br>Email: p.m.hoddinott@stir.ac.uk
**Trial Co-ordinator**	Dr Catriona O'Dolan/ Dr Lisa Macaulay<br>NMAHP Research Unit, Pathfoot Building, Stirling University, Stirling, FK9 4LA<br>Email: catriona.odolan@stir.ac.uk / lisa.macaulay@stir.ac.uk/ gameofstones@stir.ac.uk
**Sponsor**	Rachel Beaton<br>Research Integrity & Governance Manager<br>Research and Enterprise Office<br>3B1 Cottrell Building, University of Stirling FK9 4LA<br>Tel: 01786 466196<br>Email: rachel.beaton@stir.ac.uk
**Funder**	National Institute for Health Research, Public Health Research (NIHR PHR) Ref: NIHR 129703
**Clinical Trials Unit**	Prof. Graeme MacLennan<br>Director, Centre for Healthcare Randomised Trials (CHaRT), Health Services Research Unit<br>(HSRU), 3rd Floor Health Sciences Building, Foresterhill<br>Aberdeen AB25 2ZD<br>Tel: 01224 438147<br>Email: g.maclennan@abdn.ac.uk<br><br>Dr Seonaidh Cotton<br>Senior Trials Manager, CHaRT, HRSU, 3rd Floor Health Sciences Building, Foresterhill, Aberdeen AB25 2ZD<br>Email: s.c.cotton@abdn.ac.uk<br>Tel: 01224 438178<br><br>Mark Forrest<br>Senior IT Development Manager, CHaRT, Health Services Research Unit 3rd Floor Health Sciences Building, Foresterhill<br>Aberdeen AB25 2ZD<br>Tel: +44 (0)1224 438198<br>Email: mmforrest@abdn.ac.uk
**Reference Numbers**	IRAS Number: 290955<br>ISRCTN Number: 91974895<br>Clinical trials.gov Number: Not Applicable<br>SPONSORS Number: Not applicable<br>FUNDERS Number: NIHR 129703

i. SIGNATURE PAGE

The undersigned confirm that the following protocol has been agreed and accepted and that the Chief Investigator agrees to conduct the trial in compliance with the approved

For and on behalf of the Trial Sponsor:

Signature: .... Date: .17/05/2022

Name (please print): Rachel Beaton

....

Position: Research Integrity and Governance Manager...

Chief Investigator:

Signature: .... Date: 10./05 /22..

Name: (please print):.Professor Pat Hoddinott..

Statistician:

Signature: .... Date: 10./05 /22..

Name: (please print): Andrew Elders

....

Position: Statistican, NMAHP-RU, Glasgow Caledonian University... Date: 10./05 /22

CTU Director

Signature:

....

Name: (please print): Professor Graeme Maclennan

....

Position: Director, CHaRT, University of Aberdeen...

KEY PROTOCOL CONTRIBUTERS

Key Protocol Contributors<br><br>(alphabetical)	Prof Alison Avenell<br>Clinical Chair in Health Services Research, Health Services Research Unit, 3rd Floor Health Sciences Building, Foresterhill, Aberdeen AB25 2ZD<br>Tel: +44 (0)1224 438164<br>Email: a.avenell@abdn.ac.uk<br><br>Dr Paula Carroll<br>Men's Health Researcher with MFHI<br>Department Sport & Exercise Science, Waterford Institute of Technology, Main Campus Cork Road, Waterford City, Co. Waterford, Ireland<br>Tel: +353 51 83 4141<br>Email: PCARROLL@wit.ie<br><br>Asst Prof Stephan Dombrowski<br>Health Psychologist, Faculty of Kinesiology at the University of New Brunswick, 3 Bailey Drive, P.O. Box 4400, Fredericton, New Brunswick, Canada, E3B 5A3<br>Tel: 1 506 453 4803<br>Email: stephan.dombrowski@unb.ca<br><br>Mr Andrew Elders<br>Statistician, NMAHP Research Unit<br>Govan Mbeki Building, Glasgow Caledonian University, Cowcaddens Road, Glasgow, G4 0BA<br>Tel: 0141 2731307<br>Email: Andrew.elders@gcu.ac.uk<br><br>Prof Cindy Gray<br>Interdisciplinary Professor of Health and Behaviour (School of Social and Political Sciences), University of Glasgow, Rm 230, 25-29 Bute Gardens, Glasgow, G12 8RS<br>Tel: 01413306274<br>Email: Cindy.Gray@glasgow.ac.uk<br><br>Prof Fiona Harris<br>School of Health and Life Sciences<br>University of the West of Scotland, High Street, Paisley, Renfrewshire, PA1 2BE<br>Tel:<br>Email: fiona.harris@uws.ac.uk<br><br>Prof Kate Hunt<br>Institute for Social Marketing and Health, University of Stirling, Stirling, FK9 4LA<br>Tel: +44 (0)1786 466354<br>Email: kate.hunt@stir.ac.uk<br><br>Prof Frank Kee<br>Director for UKCRC Centre of Excellence for Public Health Research (NI) and Deputy Director for the Centre for Public Health, Queen's University Belfast, University Road, Belfast, BT7 1NN, Northern Ireland<br>Tel: +44 (0)28 9097 8943, +44 (0)28 906 32596<br>Email: F.Kee@qub.ac.uk

	Prof Graeme MacLennan<br>Director CHaRT, 3rd Floor Health Sciences Building, Foresterhill, Aberdeen AB25 2ZD<br>Tel: +44(0)1224 438147<br>Email: g.maclennan@abdn.ac.uk<br><br>Mr Matt McDonald<br>Feasibility study fieldworker consultant<br>Curtin University, Perth, Australia<br>Email: m.mcdonald1@postgrad.curtin.edu.au<br><br>Prof Michelle McKinley (PI GoS Belfast)<br>Professor of Public Health Nutrition<br>Queen's University Belfast, University Road, Belfast, BT7 1NN, Northern Ireland<br>Tel: +44 (0)28 9097 8936, +44 (0)28 906 32685<br>Email: m.mckinley@qub.ac.uk<br><br>Ms Rebecca Skinner<br>Feasibility study fieldworker consultant<br>Dundee University<br>Email: RSkinner001@dundee.ac.uk<br><br>Ms Claire Torrens<br>NMAHP Research Unit, Pathfoot Building, Stirling University, Stirling, FK9 4LA<br>Tel: 01786 466341<br>Email: Claire.Torrens@stir.ac.uk<br><br>Mr Martin Tod<br>CEO Men's Health Forum (GB)<br>Men's Health Forum, 49-51 East Rd, Hoxton, London N1 6AH<br>Tel: 020 7922 7908<br>Email: martin.tod@menshealthforum.org.uk<br><br>Prof Katrina Turner (PI GoS Bristol)<br>Professor in Primary Care Research / Head of Section of Applied Health Research, Joint-Head of the Centre for Academic Primary Care (CAPC), Population Health Sciences, Bristol Medical School, University of Bristol, Canynge Hall, 39 Whatley Road, Bristol, BS8 2PS<br>Tel: 0117 331 4559<br>Email: Katrina.Turner@bristol.ac.uk<br><br>Prof Marjon van der Pol<br>Health Economist, Deputy Director of Health Economics Research Unit, University of Aberdeen, Polwarth Building, Foresterhill, Aberdeen, AB25 2ZD<br>Telephone: +44 (0)1224 437172<br>Email: m.vanderpol@abdn.ac.uk
Statistician	Beatriz Goulao<br>Trial Statistician, CHaRT, HRSU, 3rd Floor Health Sciences Building, Foresterhill, Aberdeen AB25 2ZD<br>Tel: +44 (0)1224 438198<br>Email: beatriz.goulao@abdn.ac.uk

ii. VERSION HISTORY

Amendment number	Protocol version number.	Date	Author(s) of changes	Details of changes made
	1.0	23.11.20	N/A	New protocol
	2.0	17.02.21	Pat Hoddinott, Andrew Elders, Claire Torrens	Changes relate to the statistical analysis plan for secondary outcomes and Study Within A Trial (SWAT) consistent with the final versions of questionnaires (incorporate PPI and expert stakeholder feedback). Removal of minimisation by recruitment strategy due to COVID uncertainties. Minor clarifications: additional COVID vaccine question; and removal of the use of Bluetooth scales for electronic weight measurement
	3.0	28/09/21	Pat Hoddinott	Addition of PHQ-4 and changes to sub-group analyses and process evaluation to meet requirements of NIHR add-on funding application. Access to SHARE.
	4.0	27.04.22	Pat Hoddinott	Contract signed with funder for additional exploratory mixed methods analyses on mental health, multiple long-term conditions and health inequalities to inform future research:

				sections 1,3,4,6,9,12 and 13 updated accordingly.<br>Update of SWAT registration website link to published protocol<br>ISRCTN number added. Trial registered 01/04/2021<br>Updated contact details for Prof Fiona Harris<br>List of abbreviations updated

iii. LIST of CONTENTS

i. SIGNATURE PAGE 3

ii. VERSION HISTORY 6

iii. LIST of CONTENTS 8

iv. LIST OF ABBREVIATIONS 12

v. KEY WORDS 13

1. TRIAL SUMMARY 14

2. ROLES & RESPONSIBILITIES OF TRIAL MANAGEMENT COMMITEES/ GROUPS & INDIVIDUALS 17

2.1. Trial Management Committees 18

2.2. Protocol contributors 19

3. BACKGROUND 22

3.1. Rationale 22

3.2. Assessment and management of risk 24

4. OBJECTIVES AND OUTCOME MEASURES/ENDPOINTS 26

4.1. Research question 26

4.2. Primary objective 26

4.3. Secondary objectives 26

4.4. Outcome measures/endpoints 27

4.4.1 Primary endpoint/outcome 27

4.4.2 Secondary endpoints/outcomes 27

4.4.3 Exploratory endpoints/outcomes 27

4.4.4 Justification of additional baseline measures 29

5. TRIAL DESIGN 30

5.1. Trial setting 30

5.2. Participant eligibility criteria 30

5.2.1 Inclusion criteria 31

5.2.2 Exclusion criteria 31

6. TRIAL PROCEDURES 32

6.1. Recruitment and participant identification when there are no Covid-19 restrictions (standard procedures for normal times as tested in the Feasibility Study) 32

6.2.	*Strategies for addressing recruitment / assessments if Covid-19 lockdown restrictions are in place:*	*38*
6.3.	*The randomisation scheme*	*40*
6.4.	*Blinding*	*40*
6.5.	*Baseline data*	*41*
6.6.	*Trial assessments*	*41*
6.7.	*Long-term follow-up assessments*	*42*
6.8.	*Withdrawal criteria*	*42*
6.9.	*End of trial*	*43*
**7.**	**TRIAL TREATMENTS**	**44**
7.1.	*All groups - information resource and pedometers*	*44*
7.2.	*Control group*	*44*
7.3.	*Interventions*	*44*
7.3.1	*SMS*	*44*
7.3.2	*Intervention 2: endowment incentive adjunct to SMS*	*46*
7.4.	*Compliance with interventions*	*47*
**8.**	**ADVERSE EVENTS**	**48**
8.1.	*Standard definitions*	*48*
8.2.	*Operational definitions for this study*	*48*
8.3.	*Detecting AEs and SAEs*	*49*
8.4.	*Recording and assessing AEs and SAEs*	*49*
8.5.	*Review of AEs and SAEs*	*50*
8.6.	*Reporting AEs and SAEs to REC*	*50*
8.7.	*Responsibilities*	*51*
8.8.	*Safeguarding*	*51*
**9.**	**STATISTICS AND DATA ANALYSIS**	**53**
9.1.	*Sample size calculation*	*53*
9.2.	*Planned recruitment rate*	*53*
9.3.	*Statistical analysis plan*	*53*
9.3.1	*Summary of baseline data and flow of patients*	*54*
9.3.2	*Primary outcome analysis*	*54*
9.3.3	*Secondary outcome analysis*	*55*

9.4. Subgroup analyses 55
9.5. Interim analysis and criteria for the premature termination of the trial 55
9.6. Participant population 56
9.7. Procedure(s) to account for missing or spurious data 56
9.8. Other statistical considerations. 56
9.9. Economic evaluation 56
9.10. Mixed method process evaluation: 58
9.11. Nested Study Within A Trial (SWAT) 60
Qualitative Analysis 61
10. DATA MANAGEMENT 62
10.1. Data collection tools and source document identification 62
10.2. Data handling and record keeping 63
10.3. Access to Data 63
10.4. Archiving 63
10.5. Monitoring, Audit & Inspection 63
11. ETHICAL AND REGULATORY CONSIDERATIONS 65
11.1. Research Ethics Committee (REC) review & reports 65
11.2. Summary of ethical issues 65
11.3. Peer review 66
11.4. Public and Patient Involvement (PPI) 66
11.4.1 PPI contributing to the development of this protocol 66
11.4.2 How PPI will be actively involved in this trial 67
11.5. Protocol compliance and GCP 68
11.6. Data protection and patient confidentiality 68
11.7. Financial and other competing interests 69
11.8. Indemnity 69
11.9. Amendments 69
11.10. Access to the final trial dataset 69
12. DISSEMINATION POLICY 70
12.1. Dissemination policy 70
12.2. Authorship eligibility guidelines and any intended use of professional writers 71

13. REFERENCES **74**

Figures

Figure 1. Trial Flow Chart 21
Figure 2. Recruitment flowchart....................33

Tables

Table 1. Table of endpoints/outcomes 28
Table 2: Definitions of adverse events 48
Table 3. Responsibilities for collecting data on adverse events 51

iv. LIST OF ABBREVIATIONS

AE	Adverse Event
AR	Adverse Reaction
BCT	Behavioural Change Technique
BMI	Body Mass Index
CHaRT	Centre for Healthcare Randomised Trials (Aberdeen)
CI	Chief Investigator
CRF	Case Report Form
CTU	Clinical Trials Unit
DMC	Data Monitoring Committee
EQ-5D-5L	EuroQol Group questionnaire for health-related quality of life states in adults, consisting of five dimensions (Mobility, Self-care, Usual activities, Pain & discomfort, Anxiety & depression), each of which has five severity levels that are described by statements appropriate to that dimension.
EQ-5D-5L-AD	Anxiety and depression dimension of the EQ-5D-5L questionnaire
FFIT	Football Fans in Training
GCP	Good Clinical Practice
HIC	Health Informatics Centre (Dundee)
HRA	Health Research Authority
ICF	Informed Consent Form
IMD	Index of Multiple Deprivation
ISF	Investigator Site File (This forms part of the TMF)
ISRCTN	International Standard Randomised Controlled Trials Number
M	Months
MHF	Men’s Health Forum for the devolved nations
MHFI	Men’s Health Forum Ireland
MLTC	Multiple Long-Term Conditions
NICE	National Institute for Health and Care Excellence
NICRN	Northern Ireland Clinical Research Network
NIHR	National Institute for Health Research
NIHR PHR	National Institute for Health Research Public Health Research Programme
NHS R&D	National Health Service Research & Development
NMAHP	Nursing, Midwifery & Allied Health Professions

PenARC	National Institute for Health Research (NIHR) Applied Research Collaboration (ARC) South West Peninsula
PHQ-4	Patient Health Questionnaire – 4 questions relating to anxiety and depression
PI	Principal Investigator
PIL	Participant Information Leaflet
PMG	Project Management Group
PPI	Patient & Public Involvement
QA	Quality Assurance
WEMWBS	Warwick-Edinburgh Mental Well-Being Scale
RA	Research Assistant
RCT	Randomised Control Trial
REC	Research Ethics Committee
SAE	Serious Adverse Event
SAP	Statistical Analysis Plan
SDV	Source Data Verification
SHARE	Scottish Health Research Register
SIGN	Scottish Intercollegiate Guidelines Network
SIMD	Scottish Index of Multiple Deprivation
SMS	Short Messaging System
SOP	Standard Operating Procedure
SSI	Site Specific Information
SWAT	Study Within A Trial
TIDieR	Template for Intervention Description and Replication
TMF	Trial Master File
TMG	Trial Management Group
TSC	Trial Steering Committee
WSSQ	Weight Self-Stigma Questionnaire

v. KEY WORDS

Randomised Controlled Trial; Men with obesity; Text messages; Financial Incentives; Weight management; Health Inequalities, Process Evaluation.

1. TRIAL SUMMARY

**Trial Title**	Effectiveness and cost-effectiveness of text message and endowment incentives for weight management in men with obesity: The Game of Stones Randomised Controlled Trial	
**Short Title**	The Game of Stones Trial	
**Trial Design**	Multicentre randomised controlled trial with three arms: text messages with incentive (SMS+I) text messages only (SMS); and waiting list for text messages (Control)	
**Trial Participants**	Any man aged 18 or over with a Body Mass Index of 30kg/m2 or more resident in and around Glasgow, Belfast and Bristol. Men planning to move out of the area within 12 months or who are planning bariatric surgery in the next 12 months will not be eligible.	
**Planned Sample Size**	585	
**Intervention duration**	12 months	
**Follow up duration**	Up to 24 months	
**Planned Trial Period**	42 months	
	**Objectives**	**Outcome Measures**
**Primary (12 months)**	To conduct a 3-arm RCT to estimate between group % difference in weight-loss at 12 Months (M) from baseline for men with obesity who receive i) SMS+I; ii) SMS only; iii) 12M wait list for SMS	Within-participant change from baseline weight expressed as a percentage of baseline weight at 12 months.
**Secondary (12 months)**	• Assess differences between groups in secondary outcomes	• Absolute weight change at 12m from baseline (Kg); 0<5%; ≥5-<10% and ≥10% weight loss; % losing any weight; Satisfaction; Warwick-Edinburgh Mental Well-Being Scale; EQ-5D-5L; Confidence in ability to lose weight and maintain weight loss long term; behaviours:; physical activity, alcohol frequency; smoking status; and weight management strategies used; adverse events<br>• PROCESS OUTCOMES: incentives gained/targets met; SMS delivered; SMS

		and web page engagement; GP/Community recruitment strategy<br><br>• NHS costs: QALYs, incremental cost per QALY and incremental cost per % weight loss
	• Assess the cost-effectiveness of SMS+I and SMS only compared to a wait list control over trial period and modelled lifetime<br>• Understand men's and service providers' experiences of the intervention. Explore men's perspectives on mental health and multiple long-term conditions<br>• Exploratory sub-group and mixed methods analyses: recruitment strategy; mental health; multiple long-term conditions; co-morbidity and health inequalities to inform future research and implementation<br>• Follow up men at 24M (12M after intervention ceases) and request consent for linkage to long-term health outcomes<br>• Refine the digital programming for future scalability and implementation	• Telephone/remote interviews with 30-54 participants about their experiences at 12M and 24M<br>• Mean between-group % differences in weight-change at 24M from baseline; and from 12M<br><br>• Stakeholder involvement and qualitative interviews (n=12-16)
	**PhD Student – nested SWAT RCT:**	Primary:<br>• Retention rate at 12 months .

	• To evaluate the effect on retention of two different weight assessment approaches undertaken with Game of Stones SMS+I and SMS participants during 3 and 6M assessments<br>• To explore men's and researchers' experience of two different weight assessment protocols delivered at 3 and 6M	Secondary:<br>• Relationship: Participants at 12M<br>• Weight Self-Stigma Questionnaire (WSSQ) (Participants) baseline and 12M<br><br>Other/ Exploratory:<br>• Weight Bias (Fieldworkers) baseline and 12M<br>• Empathy (Fieldworkers) baseline and 12M<br>• Relationship Fieldworkers after 3 and/ or 6M weight assessments<br>• Qualitative telephone/remote interviews with Fieldworkers and participants to explore their experiences at 12M<br>• Fidelity check of protocol adherence by researchers using audio-recordings of participant-researcher interactions at 3 and 6M assessments.

2. ROLES & RESPONSIBILITIES OF TRIAL MANAGEMENT COMMITEES/ GROUPS & INDIVIDUALS

Pat Hoddinott will lead the trial and take overall responsibility, supported at NMAHP-RU by Catriona O'Dolan & Lisa Macaulay as experienced Trial Managers (job-share) and grant-holders: Kate Hunt (deputy for PH), Fiona Harris (qualitative oversight); Stephan Dombrowski (SMS and health psychology lead who holds an honorary researcher position at Stirling) and Andrew Elders (NMAHP-RU trial statistician/methodologist). Pat Hoddinott, Michelle McKinley and Katrina Turner will be Centre leads. CHaRT registered CTU (Director Graeme MacLennan), University of Aberdeen, will provide Senior Trial Manager input (Seonaidh Cotton) and will lead randomisation, data management, analysis and database programming working closely with Claire Jones (HIC, University of Dundee) who will continue to deliver the SMS+I interventions and participant tracker software which worked well in the Feasibility Study. Marjon Van der Pol (Aberdeen, senior health economist) will lead the health economic analysis. Alison Avenell, Frank Kee (Public Health Methodologist) and Cindy Gray will contribute obesity, public health and incentive trial expertise, with Martin Tod from Men's Health Forum GB, Colin Fowler and Paula Carroll from Men's Health Forum All Ireland contributing Public and Patient Involvement.

Centre Management Meetings: fortnightly/monthly (depending on stage of trial) with the Centre Leads, Fieldworkers and the trial managers, with others invited when required.

Co-Investigator Project Management Team Teleconferences: as required every 2-4 months, with email updates between. Grant holders are involved in overseeing all aspects of study conduct.

The Programming, Statistics, Health Economics and Data Team will consist of Mark Forrest, Claire Jones, Graeme MacLennan, Andrew Elders, Beatriz Goulao, Trial Managers, Marjon van der Pol and Pat Hoddinott. Meetings will take place monthly in Phase 1, and every 1-3 months thereafter, with email and teleconferences as required. Andrew Elders will lead the statistical analysis plan, oversee the statistician undertaking the statistical analysis at CHaRT and the statistical tables for the report and papers. The CHaRT statistician will conduct the final analysis blind to group allocation, produce data for reports and publications. The CHaRT team includes a senior trial manager, senior IT support, a programmer and quality assurance staff.

The Qualitative Team will be overseen by Fiona Harris supervising Claire Torrens, qualitative Research Fellow (RF), who will be the lead at Stirling, with Kate Hunt, Cindy Gray, Katrina Turner, Stephan Dombrowski, Michelle McKinley, Centre RAs, the Trial Managers, PPI from Men's Health Forum and Pat Hoddinott. Meetings will take place: early in the trial to plan data collection; in stage 2 to finalise the thematic framework and coding index; and to interpret the findings for report writing. Teleconferences will be held as required.

2.1. Trial Management Committees

The committees involved in trial coordination and conduct are documented in the Trial Steering Committee Charter. The Co-Investigators are listed on https://fundingawards.nihr.ac.uk/award/NIHR129713 and both Co-investigators and trial staff will be listed on www.gameofstonesresearch.com

Trial Steering Committee (TSC) (includes data monitoring role as agreed with NIHR)

The Independent TSC will meet at least annually face to face or online as required to oversee all aspects of the trial, with accountability to the funder and the sponsor. As this trial involves a low-risk self-management intervention, the TSC will also fulfil the role of monitoring safety and make recommendations as to any modifications that are required to be made to the protocol or the termination of all or part of the trial. Six independent members have agreed to join. A Trial Statistician and methodologist (continuing as chair from the previous Feasibility Study); a Public Health expert in obesity trials; a Public Health Local Authority Commissioner; a Health Economist and two lay members with expertise in Men's Health. A Charter based on HRA guidance will be signed by members and updated as required.

TSC roles will be to review the protocol and assess two stage traffic light monitoring as recommended in guidance.

i) progression criteria after 4 months of recruitment, for % of total target sample size achieved:

- Green: >50% of target sample size after 4 months. Proceed.
- Amber: 25-49% modify recruitment strategies:
- Red: <25% consider stopping in discussion with the funder and the sponsor

ii) progression criteria in ~study month 34 will be for the TSC to decide if a clinically significant difference in the primary outcome has been met for at least one comparison. The TSC in discussion with NIHR may consider other core outcome criteria e.g. absolute weight change (kg); health inequalities; % of men achieving ≥5% or ≥10% weight loss. If NIHR agreed criteria are met, we will progress to Phase 3 and complete: 24M data collection and analysis; stakeholder scalability interviews; full effectiveness and cost-effectiveness modelling; and plan for future data linkage. If criteria are not met, the study could end before 42 months. If the study does end before all 24M follow-up data has been collected, existing data will be analysed.

Project Management Group

The Project Management Group (PMG) consists of the NIHR Grant holders https://fundingawards.nihr.ac.uk/award/NIHR129713 with key trial staff (trial managers, research fellows, statistician; programmers) and will meet every 2-4 months remotely as required. They will ensure all practical details of the trial are progressing and working well and everyone within the trial understands them. Observers will be invited to attend at the discretion of the PMG. The PMG will meet/teleconference every three months on average, but monthly in the initial set-up stage.

Trial Coordinating Team

The trial will be coordinated by the CI and Trial Managers based at NMAHP RU at Stirling University. The Trial Managers will be responsible for the day to day transaction of trial activities (supported by Seonaidh Cotton, senior trial manager at CHaRT) and coordinating work conducted by the Fieldworkers at each of the 3 centres via their Centre Leads. The Fieldworkers are responsible for day to day recruitment and follow up of participants; data collection and management; liaising with PPI and Stakeholders and assisting the process evaluation team. Two Fieldworkers at each centre will be supported by a pool of postgraduate students, so that recruitment can occur flexibly at weekends and in the evenings, enabling men who work to join the study. This was effective and efficient in the Feasibility Study.

A trial-specific delegation log will be prepared for each centre, detailing the responsibilities of each member of staff working on the trial.

The CI, Trial Managers and Qualitative Research Fellow will meet weekly, with others invited when required. The CI, Trial Managers and Centre Leads will meet fortnightly/monthly (depending on stage of trial), with others invited when required.

2.2. Protocol contributors

- Pat Hoddinott compiled the first draft of the protocol for this full trial, by updating V 2.2 of the successful Feasibility Study protocol to incorporate the changes advised by study participants, PMG, TSC, Funders and for the Covid-19 context. Few changes to the feasibility protocol are required as the study was feasible, acceptable and recruited to target [1]. The V 2.2 Feasibility Study protocol was co-written by Stephan Dombrowski and Pat Hoddinott as Joint Chief Investigators.
- Andrew Elders drafted the sample size and statistical analysis sections of the protocol, with contributions from Beatriz Goulao and Graeme Maclennan.
- Marjon Van der Pol drafted the health economic sections
- Claire Torrens drafted sections describing the Nested RCT Study within A Trial (SWAT)

- Pat Hoddinott, Claire Torrens and Fiona Harris drafted the qualitative sub-study
- Catriona O'Dolan, Lisa Macaulay, and Seonaidh Cotton (Senior trial manager at CHaRT) helped to draft and edit this protocol. Catriona O'Dolan and Lisa Macaulay led on the ethics application and the adaptation of the Feasibility Study public facing materials which were approved by the North of Scotland Research Ethics Committee (Ref 16/NS/0120**)**.
- Matthew McDonald and Rebecca Skinner commented on trial recruitment and assessment processes, informed by their experiences as Research Assistants in the Feasibility Study.

Men in the target population, Men's Health Forum and other members of the public have been involved in all aspects of the Game of Stones Feasibility Study, as described in the Final Report to NIHR for the Feasibility Study [2] and in Section 11.4.

Figure 1. Trial Flow Chart

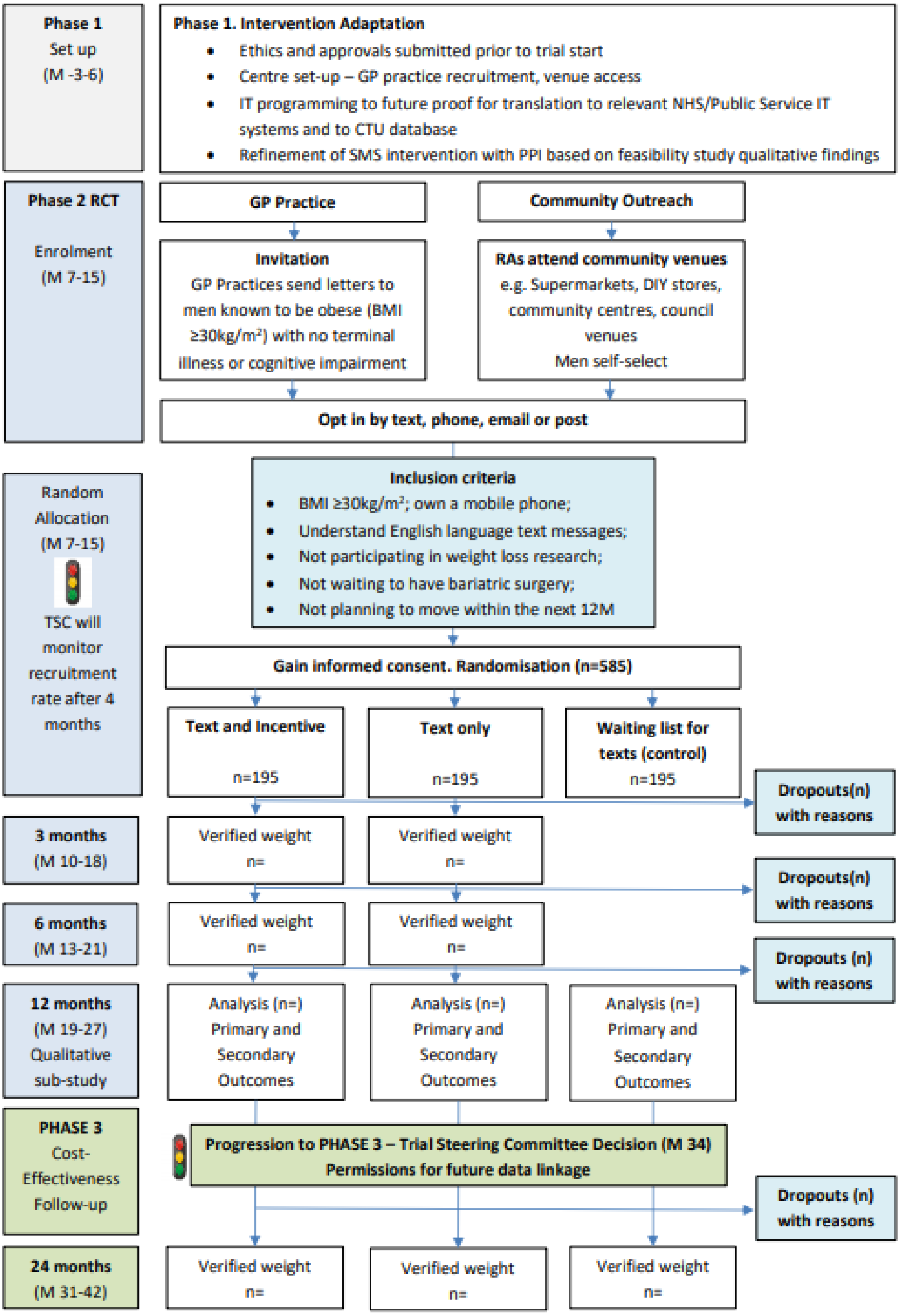

3. BACKGROUND

In this randomised controlled trial we are investigating effective and cost-effective interventions that may engage men with obesity to achieve clinically significant weight loss and maintain that weight loss, particularly in areas where there are health inequalities. Men engage less often than women in existing weight loss interventions [3] and sustainable interventions with broad reach are needed for men who do not like or cannot access groups. Yet men die sooner than women and year-on-year mortality improvements have slowed, particularly in disadvantaged areas [4, 5]. Obesity increases the risk of type 2 diabetes, heart disease, stroke, mobility problems and some cancers, leading to multi-morbidity. Weight loss can reduce premature all-cause mortality and reverse the early stages of type 2 diabetes, and therefore reducing obesity is a UK Government priority. Recent Office for National Statistics data demonstrate that obesity and health inequalities are risk factors for worse health outcomes for people with Covid-19.

This proposed trial is therefore important to the public, the NHS and society. In 2017, 27% of men in England were obese; this prevalence is higher than elsewhere in Europe and predicted to increase[6, 7]. In 2017/18, there were 711,000 admissions to English hospitals where obesity was recorded as the primary or secondary diagnosis, a 15% increase from 2016/17 [6, 7].

Recent reports recommend testing innovative, scalable, digital interventions for reducing obesity [8]. Scalable digital interventions like Game of Stones can increase reach to men and promote self-management. They can be offered within current NICE recommended tiered weight management services [9] to span primary, secondary and tertiary disease prevention.

Game of Stones is a parallel, 3-arm RCT which delivers automated SMS (short message system) texts with or without financial incentives and compares weight change at 12 months with a waiting list for SMS, for men with obesity. Our promising Feasibility Study demonstrated broad acceptability. It was over-subscribed and 60% of the 105 men participating lived in the two most disadvantaged Scottish Index of Multiple Deprivation (SIMD) postcode quintiles, which is more than in other UK obesity trials [1, 2, 10]. Fewer men living in disadvantaged areas dropped out compared to men living in more advantaged areas. An independent Trial Steering Committee judged that progression criteria for a full RCT were met and this full RCT will follow a similar trial protocol with some modifications for Covid-19.

3.1. Rationale

The research question is: Are automated ‘Short Message System’ (SMS) texts, delivered to support behaviour change, with or without endowment Incentives (I), effective and cost-effective for weight-loss at 12 months compared to a waiting list control in men with obesity?

We have conducted four systematic reviews of the quantitative, qualitative and economic evidence for the management of obesity in men [11]. The rationale for the SMS and incentive interventions in Game of Stones is supported by systematic review evidence. We have completed a systematic review and meta-analysis of 15 RCTs examining SMS-delivered interventions for weight loss (n=12) and weight loss maintenance (n=3) [12]. The weight loss trials had an effect at intervention end (median duration 6 months) with a mean difference -2.28kg (95% confidence interval [CI] -3.18 to -1.36 kg). Men accounted for 41% of trial participants, higher than for other interventions [11], but there were no men-only trials and health inequalities data were not reported. The ROMEO reviews [11] identified that interventions need to be designed with men in the target population. In Game of Stones, we worked closely with Men's Health Forum Charities and men in the target population to write the text messages informed by qualitative data analysis, to ensure the language, content, frequency and timing are acceptable [2].

INCENTIVES: Systematic review evidence of financial incentives shows their potential to change habitual behaviours and help reduce health inequalities [13]. Moreover, the evidence for financial incentives for weight loss is growing [14] and deposit contracts can be effective [15]. But deposit contracts, where people put their own money into an account, losing it if weight loss targets are not met, may increase health inequalities for the cash poor. In a discrete choice experiment (DCE) we conducted with 1045 men with obesity to design an incentive, with high uptake across all men [2]. In comparison, this cost is similar to attending commercial weight loss groups; or annual prescribing costs for the weight loss drug Orlistat (excluding clinical appointments and blood test monitoring costs) – which men do not find helpful [11].

Pedometers are given to all participating men in Game of Stones to encourage self-monitoring. All men (including wait-list arm) will have access to a Game of Stones website which includes links to evidence-based information about weight loss, and to charities like Men's Health Forum which support men's physical and mental health.

This trial will recruit from diverse areas in three UK countries to provide generalisable evidence on effectiveness, cost-effectiveness and impact on health inequalities relevant for policy makers and service commissioners. Programming for transferability will involve public service technology experts so that SMS and incentives can in future be delivered centrally or locally, together or separately. Measuring weight by health care assistant or equivalent staff in health or community venues was feasible [2] . This self-care intervention can accommodate future technological advances in digital scales linked to databases to minimise staff resource requirements. Commissioners tell us that variations in effect sizes

by socio-economic group and levels of co-morbidity will be important to inform how Game of Stones might contribute to current weight management services.

In summary, Game of Stones targets a clear evidence gap in weight management services and health inequalities for men. Obesity, health inequalities and prevention are all UK policy priorities in the NHS Long Term Plan (www.longtermplan.nhs.uk) and are likely to remain so for the foreseeable future, particularly with new evidence emerging about risk factors for Covid-19. In a female dominated weight management landscape, NHS service commissioners want to know whether financial incentives with text messages can make a difference to improve obesity outcomes for men.

The research team were invited to apply for additional funding from NIHR (submitted September 2021) to investigate UK policy priority areas: mental health conditions and multiple long-term conditions within the Game of Stones Trial. A new secondary outcome Patient Health Questionnaire – 4 (PHQ-4) was approved by the Ethics Committee in September 2021 and baseline data commenced. Funding was approved in January 2022 and contract variation signed on 1st March 2022. Adults with obesity are more likely to be depressed than those without [67] and to experience generalised anxiety disorders. [68] Health inequalities are apparent for both people with mental health problems and with obesity, with Covid-19 highlighting disparities. [69] Mental illnesses are associated with a 1.4 to 2-fold increased risk for obesity, diabetes and cardiovascular diseases compared to the general population. [70] The causal pathways for obesity, mental health and health inequalities are complex and multi-directional, and there is an evidence gap for men living with obesity.

There is an evidence gap in existing systematic reviews for men living with MLTC and obesity who participate in weight management intervention trials. [71, 72] Many trials of weight management/dietary change interventions focus on participants with single non-communicable conditions. However a large UK primary care cohort highlights the high prevalence of co-morbidities in patients with Type 2 diabetes. [73] We have undertaken qualitative interviews with 8 senior UK decision makers during the set-up of this trial and diabetes is a priority condition.

Additional exploratory sub-group analyses and mixed method process evaluation are described in detail in the Statistical Analysis Plan and Process Evaluation Plan and will be reported separately from the primary trial effectiveness and cost-effectiveness outcomes. This effectiveness and cost effectiveness trial of Game of Stones will produce evidence that service commissioners, the NHS and the public require.

3.2. Assessment and management of risk

This is very low risk automated digital intervention to support and motivate self-care in men with obesity who want to lose weight. It is a Public Health Trial and not a clinical trial. Recruitment is equitable and

inclusive, open to any member of the general public who meets the eligibility criteria. Adverse events will be reported and monitored as outlined in Section 8 of this protocol.

Research Fieldworkers' safety will follow appropriate guidance from the University where they are employed, and local risk assessments completed where necessary. The researchers may work in pairs when recruiting or work alone, according to the neighbourhood and as advised by stakeholders with local knowledge. Principle Investigators will ensure that centre staff can undertake fieldwork in a Covid-19 safe way.

4. OBJECTIVES AND OUTCOME MEASURES/ENDPOINTS

4.1. Research question

Are automated 'Short Message System' (SMS) texts, delivered to support behaviour change, with or without endowment Incentives (I), effective and cost-effective for weight change at 12M compared to a waiting list control in men with obesity?

4.2. Primary objective

To conduct a 3-arm RCT to estimate between group difference in % weight change at 12M from baseline for men with obesity who receive i) SMS+I; ii) SMS only; iii) 12M wait list for SMS

4.3. Secondary objectives

- To assess differences between groups in secondary outcomes
- To assess the cost-effectiveness of SMS+I and SMS only compared to a wait list control
- To understand men's and service providers' experiences of the intervention
- To follow up men at 24M (12M after texts/incentives cease for the intervention groups; 9 months after the SMS cease for the waiting list group). Request consent for linkage to long-term health outcome data
- To refine the digital programming for future scalability and implementation
- To compare PHQ-4, Warwick and Edinburgh Mental Wellbeing Scale (WEMWBS), Quality of Life (EQ-5D-5L-AD) Anxiety and Depression (AD) dimension and Weight Self-Stigma Questionnaire (WSSQ) measures for the 3 trial groups at baseline and 12 months
- To undertake exploratory moderator analyses examining interactions between mental health/wellbeing status at baseline and 12-month weight change
- To undertake exploratory mixed methods analyses examining mental health/wellbeing status, weight change trajectories, health inequalities, lived experiences and views to inform implementation, tailoring and future research
- To undertake exploratory moderator analysis for weight change at 12 months by the presence/absence of multiple long-term conditions (with and without diabetes) at baseline
- To compare secondary Quality of Life, mental health/wellbeing outcomes for men with or without MLTC at baseline
- To undertake exploratory mixed method analyses to understand the lived experiences of men with MLTC and what would make a difference for recruitment, implementation, tailoring and future weight management interventions.
- To undertake a mixed method process evaluation to inform future implementation and research

4.4. Outcome measures/endpoints

4.4.1 Primary endpoint/outcome

Demographics and outcome measures (Table 1) are based on the FFIT trial [16], and the recently published STAR-LITE core outcome set for behavioural weight management interventions [17].

The Primary outcome is within-participant change from baseline weight expressed as a percentage of baseline weight at 12 months from baseline. The trial is powered on a 3% weight loss, which NICE states is clinically significant and consistent with STAR-LITE Core Outcome set for obesity trials [17].

4.4.2 Secondary endpoints/outcomes

Secondary outcomes at 12 months: absolute weight change from baseline (kg); % of participants achieving 0<5% weight loss; ≥5<10% weight loss; % of participants achieving ≥10% weight loss, % of participants losing any weight, % of participants gaining weight; EQ-5D-5L; EQ-5D-5L-AD; WEMWBS; PHQ-4; WSSQ.

Health economic outcomes: NHS costs, QALYs, incremental cost-per QALY gained and incremental cost per % weight loss over trial follow-up and modelled lifetime.

4.4.3 Exploratory endpoints/outcomes

Exploratory endpoints/outcomes at 12 months: weight management strategies used; self-monitoring activity weight; self-monitoring steps; physical activity; alcohol; smoking; satisfaction with Game of Stones; satisfaction with weight loss progress; confidence in ability to lose weight; confidence in ability to maintain weight loss long term.

Outcomes at 24 months: weight change (absolute Kg, %) from baseline and from 12 months. Depending on the advice of the TSC for Phase 3, additional outcomes may be reported (Table 1)

In our Feasibility Study the following were found to be acceptable and feasible to collect high quality data: physical activity [18]; alcohol consumption [19]; smoking status [20]; satisfaction with interventions (Feasibility Study tool); Warwick-Edinburgh Mental Well-Being Scale (WEMWBS) [21] and weight management strategies used over last 12 months. This question has been adapted by Co-Investigator Dombrowski from the top 10 effective strategies adapted from Hartmann-Boyce (2016) Oxford Food and Activity Behaviours (OxFAB) Cohort Study [22]. Dombrowski has used this in a recent survey of 1000 men with obesity in Canada (unpublished –in progress).

Process outcomes: incentives gained; weight change (absolute, %) at 3 and 6 months; number and chosen frequency of SMS delivered; any responses to SMS; web page use; recruitment and retention

by recruitment strategy, health inequalities: perceived wealth[23]; financial strain[24, 25] to explore the UK cost of living crisis unfolding during this trial.

Qualitative interview data with men at 12 and/or 24M will generate findings which will help to understand experiences and behaviours of men during and after the interventions and barriers/facilitators to longer term sustained weight loss and scalability of the intervention.

Addressing health inequalities, mental health and multiple long-term conditions (MLTC) are key government priorities, together with understanding the mediators and moderators for weight management. These are all inter-related and casual pathways are complex and multi-directional.

A parallel integrated mixed methods exploratory analysis will be conducted for weight change (%) focusing on the quantitative subgroup analyses described in 9.4 for 1) socio-economic status 2) mental health and wellbeing 3) co-morbidity and multiple long-term conditions (with and without diabetes); other effects (satisfaction, confidence in ability to lose weight and maintain weight loss). We will measure mental health and wellbeing using a range of validated questionnaires to inform a multi-lens approach moderator analysis. This is important to address the complexity of experiences reported by men involved in our feasibility study, PPI feedback, the diverse recruitment strategies and to maximise knowledge around health inequalities. MLTC is defined as the co-existence of two or more chronic conditions where obesity is a recognised risk factor - stroke including mini-stroke, high blood pressure, a heart condition, diabetes, cancer, arthritis, mental health problem or self-report disability (ONS standardised definition). Obesity is a risk factor for MLTC but is not currently classified as a disease and will not be included. More detail is provided in Section 9.

Table 1. Table of endpoints/outcomes

Outcome assessment schedule according to STAR-LITE core outcome set [17], obesity programmes checklist from the Men's Health Forum [26] and Feasibility Study findings [2].

Data Collection	***0M**	**12M**	****24M**
Socio-demographic: IMD, co-morbidities (physical and mental health), self-report disability, ethnicity, age, education, employment, household size; relationship status.	✓		
Perceived wealth, financial strain	✓	✓	✓
Anthropometry - height (for BMI)	✓		
Anthropometry – weight	✓	✓	✓
Participant satisfaction		✓	✓
Health behaviours - physical activity, smoking status, alcohol frequency	✓	✓	
Weight management strategies used over last 12 months	✓	✓	✓

Confidence in ability to lose weight and maintain weight loss	✓	✓	✓
PHQ-4	✓	✓	
EQ-5D-5L – Anxiety and Depression Dimension (AD)	✓	✓	
Warwick-Edinburgh Mental Well-Being Scale (WEMWBS)	✓	✓	
Weight Self-Stigma Questionnaire (WSSQ) (included in SWAT PhD)	✓	✓	
Health Economic Outcomes: EQ-5D, NHS health care use; public sector weight management services	✓	✓	✓
Qualitative interview data (experiences, behaviours)		✓	✓
Process outcomes: observed weight meets target and incentives secured/lost at 3M and 6M; number of SMS delivered; SMS responses, web page use over 12M	✓	✓	
Unintended consequences or adverse events		✓	✓

*OM = Baseline i.e. pre-randomisation; **Phase 3 of trial guided by Trial Steering Committee

4.4.4 Justification of additional baseline measures

Mental Health: NICE recommends that practitioners use PHQ-4 to ascertain whether a mental health assessment is required using validated measures of depression (PHQ-9) and anxiety (GAD-7). [7] PHQ-4 consists of the first two questions of the PHQ-9 and GAD-7 and is an appropriate validated outcome measure for a general adult population [8]. Qualitative research found that separate assessments of anxiety and depression can be unhelpful. [9] Our Public and Patient Involvement (PPI) state mental health as a key issue for weight loss; preferred a wellbeing rather than a “condition” focus and advised us to consider the negative burden of longer depression/anxiety questionnaires. To understand more about men living with obesity and mental health issues the following baseline characteristics will be described: PHQ-4, EQ-5D-5L-AD, self-report mental health condition diagnosed by a doctor, PHQ-4, WSSQ, self-report disability (ONS standardised question), lives alone (ONS standardised question) and alcohol frequency. A new baseline variable will be generated called “possible latent mental health condition” and will be defined in the Statistical Analysis Plan. The Office for National Statistics England 2021 reports that common mental disorders are under-reported in men compared to women in all age categories, with fewer men referred to the NHS Improving Access to Psychological Therapies (IAPT) programme.[74]

Multiple Long-Term Conditions present at baseline are defined as the co-existence of two or more chronic conditions where obesity is a recognised risk factor - stroke including mini-stroke, high blood pressure, a heart condition, diabetes, cancer, arthritis, mental health problem (Feasibility Study question) or self-report disability (ONS standardised definition). Obesity is a risk factor for MLTC but is not currently classified as a disease and will not be included. Baseline characteristics will be described for the presence of MLTC and the presence of MLTC where one of the conditions is diabetes.

5. TRIAL DESIGN

A pragmatic, multi-centre, parallel, 3-arm, assessor blind, RCT comparing weight change at 12M for: i) SMS with endowment incentive; ii) SMS only and iii) 12-month waiting list control for SMS delivered M13-15 inclusive), with 24M follow-up (i.e. 1 year post intervention), mixed methods process evaluation including qualitative interviews (participants and commissioners), cost-effectiveness modelling and consent for future data linkage to longer term health outcomes. There are three phases: 1. Trial set-up and integrating scalability; 2. RCT; 3. Long-term cost effectiveness, scalability and follow-up.

A secure CTU web-based system will be used to randomise men, stratified by recruitment centre.

5.1. Trial setting

There will be three recruiting centres in and around our Glasgow; Belfast and Bristol, with multiple recruiting venues across broad geographical areas linked to each centre. Community and GP practice venues will be purposively selected for socio-economic disadvantage, ethnic and geographic diversity (urban, suburban, town, rural).

5.2. Participant eligibility criteria

Socioeconomic position and inequalities: Our aim is to have an inclusive approach with broad eligibility across all sectors of society. Game of Stones SMS can be delivered to any mobile phone at any time or place and are scalable, so can include men from disadvantaged backgrounds [27]. In 2019, 93% of men and 89% of adults in financially vulnerable households had access to a mobile phone [28]. Some group-based weight management interventions are particularly unappealing to men from disadvantaged areas [29], and individual remotely delivered interventions like Game of Stones are potentially able to engage these men. In this protocol we use the term “harder to reach” as a summary phrase for men who may experience a wide range of disadvantage in their circumstances. This may include but is not limited to: low income; poverty; working anti-social, un-predictable hours; relationship problems; loneliness; poor mental health; mobility or access difficulties.

By targeting disadvantaged areas for recruitment, we aim to recruit a similar proportion of men living in disadvantaged areas as in our Feasibility Study where 60% of participants lived in SIMD 1 and 2 postcode areas and a higher proportion of men from SIMD 1 and 2 postcode areas attended follow-up at 12M than men from higher SIMD. This is more than any other recent UK obesity RCT and the majority of international men-only RCTs [11, 12, 16]. Game of Stones SMS appealed to the men as they encouraged autonomy, were non-stigmatising and low burden. The spaced-out assessment visits (four over 1 year) and minimal time commitment was also attractive to men living in disadvantaged areas. Offering an incentive was valued by men on low incomes in our Feasibility Study for a variety of reasons, such as losing weight resulting in them needing to buy new trousers. A Food Foundation report highlights

that if people follow Public Health England's healthy eating advice, those in the bottom 10% of income would need to spend 74% of their household income on food [30], further highlighting the importance of health inequalities.

5.2.1 Inclusion criteria

- Men with BMI equal to or greater than 30kg/m^2
- Aged 18 or over, understand study information and able to give informed consent.
- Resident in and around Glasgow, Belfast and Bristol.

5.2.2 Exclusion criteria

- Inability to understand the trial or the English language SMS intervention
- No mobile phone access
- Planning to move out of the area within 12 months
- Current or recent (in last 6 months) participation in a research weight loss intervention study (participants from the feasibility study are welcome to participate in this RCT)
- Plan to have bariatric surgery within 12 months.
- For GP screening prior to sending invitation letters
    - known terminal illness or severe psychiatric illness
    - known impaired cognitive or visual function that would limit understanding of study information and SMS.

6. TRIAL PROCEDURES

The Covid-19 situation when this trial commences recruitment in spring 2021 is uncertain. It is possible that different sites may have different restrictions operating at the same time. A log of restrictions operating will be kept by the site staff and the type of contact: face to face; video; other (specified) will be documented for each participant contact and outcome assessment. In Section 6.1 the trial procedures undertaken in the Feasibility Study are described. These will be followed where possible. In Section 6.2, strategies for Covid-19 restrictions are described.

During the 24 hours prior to any face-to-face contact, the participant will be contacted and asked the following questions, which will be documented on CHaRT stand-alone form:

1. Have you recently been diagnosed with Covid-19? (definition of recent to be informed by current guidance)
2. Have you had a Covid 19 vaccine?
3. Do you currently believe that you may have Covid-19?
4. Do you currently have any symptoms to suggest Covid-19 infection? (specific questions about fever, cough according to current NHS guidance, including temperature assessment is recommended in guidance)
5. Have you recently been in contact with Covid-19 and told to self-isolate?
6. Have you returned from another country in the last 14 days, where isolation rules apply?

Additional questions may be asked in line with current national or local guidelines. The Fieldworker will confirm to the participant their answers to the same questions. If either participant or researcher answer yes, then face to face contact should not occur.

Should either the Fieldworker or participant experience Covid-19 symptoms or test positive for the virus after a face to face meeting has taken place, local contact-tracing procedures will be followed.

6.1. Recruitment and participant identification when there are no Covid-19 restrictions (standard procedures for normal times as tested in the Feasibility Study)

The recruitment strategies are those used in the Feasibility Study, approved by the North of Scotland Ethics Committee (Ref: 16/NS/0120) and published [1, 2, 10].

Figure 2 is a flow chart summarising the procedures.

Figure 2. Recruitment flowchart

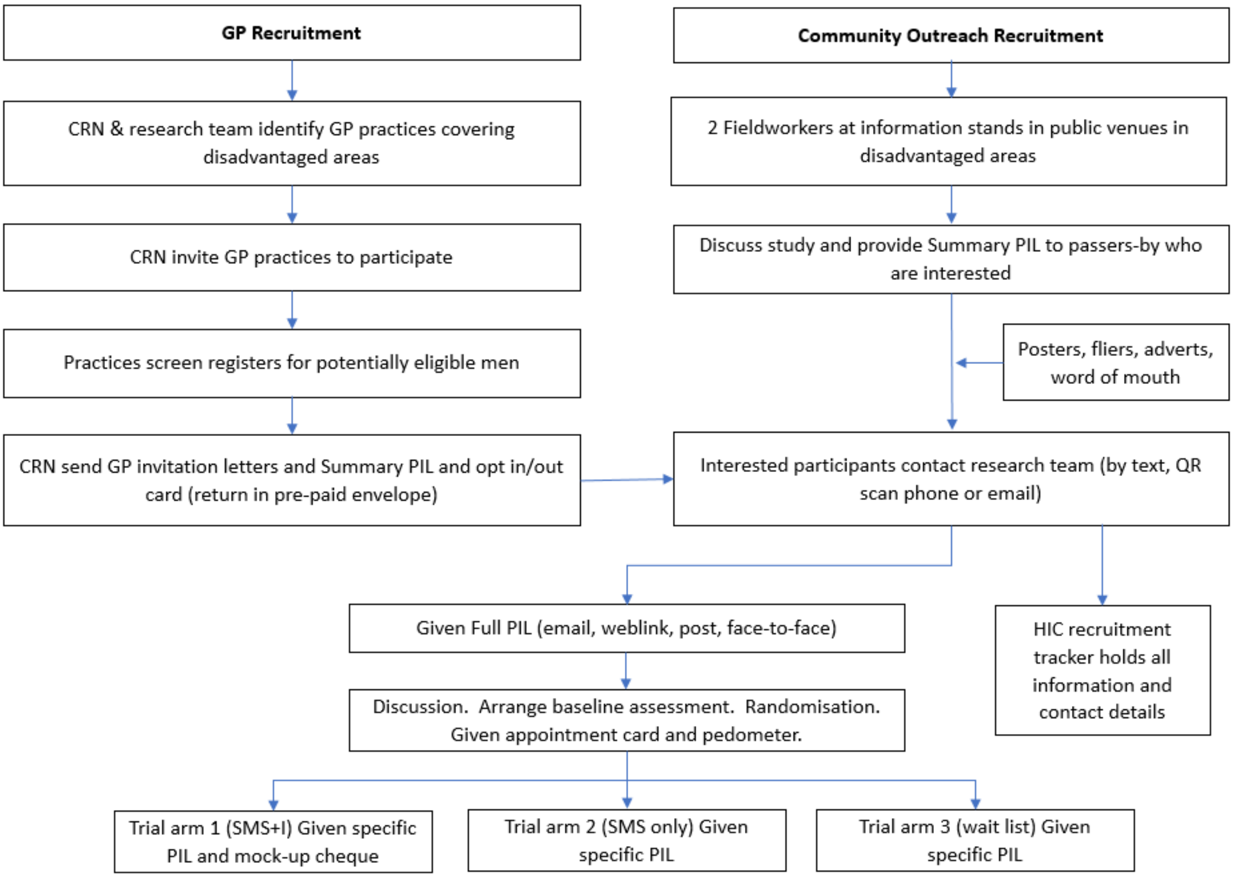

We will recruit 585 men with obesity (see Section 9.1 for sample size justification) through GP obesity registers and community outreach across the three wide geographical areas linked to the trial centres. The acceptability and feasibility of this dual approach to recruitment was demonstrated in our Feasibility Study, which was over-subscribed. In the Feasibility Study, more men were interested than could be accommodated (n=105). Most participants (60%) lived in more disadvantaged areas. Compared to community outreach, men recruited via GP letters were older (mean=57 vs 48 years); more likely to report an obesity-related co-morbidity (87% vs 44%); and less educated (no formal qualifications, 32% vs 10%, degree educated 11% vs 41%). Our recruitment strategy is therefore unchanged from our Feasibility Study and will recruit through both GP letter and community venues. The proposed strategy described below has been successfully used in other NIHR studies to recruit men and obese men for a narrative SMS intervention for alcohol reduction [31-33] which were delivered using the same University of Dundee software.

i) Recruitment through GP practice obesity registers (approximately 50% of sample). Body Mass Index (BMI) is frequently recorded in General Practice records for patients presenting with or undergoing review of long-term conditions e.g. diabetes, heart disease and high blood pressure. Identification and inviting eligible men will be undertaken by the NRS Primary Care Network in Scotland who successfully managed this process in the Feasibility Study, by the National Institute for Health Research (NIHR) Clinical Research Networks in Northern Ireland (NICRN) and West of England (CRNWoE). Research Networks and Game of Stones researchers will identify GP practices covering disadvantaged areas. Research networks will approach practices, discuss the trial and provide a "GP invitation to participate letter". Network staff will work initially with approximately 10 consenting GP practices in each of the three geographical areas (depending on practice size; known obesity prevalence), to identify eligible men from practice data. In the Feasibility Study, GP letters had an opt-in rate of 10.2% (n=90/879). The practices will identify men whose most recent documented BMI was 30 or higher. Clinical staff in the practice will screen the list of potentially eligible men and remove any men who are not eligible (see Section 5.2). The Clinical Research Networks will arrange for eligible lists to be uploaded to the secure recruitment tracker database at the Health Informatics Centre at The University of Dundee. Clinical Research Networks will send a Summary PIL (site headed paper), an invitation letter on GP practice headed paper and a pre-paid envelope with an opt-in card using Docmail or regular mail. The opt-in card will ask participants to indicate whether they are interested in receiving further information about the study or not. Those who indicate an interest will be asked to record their contact details on the card. Practice staff will be asked to remind eligible men about the study and to put up a poster in the waiting room. Where possible, members of the research team will be available in the waiting room at set times to discuss the study with men and practices will be requested to provide a room for enrolling and collecting data from men. If recruitment is slower than expected:

- The 50:50 ratio of GP to community recruits will be flexible, according to which method is recruiting most successful and the COVID situation
- The number of GP practices sending invitation letters will be increased
- Clinical research network/research team visits/phone calls will be made to GP practices to discuss the barriers and facilitators to recruitment
- Follow-up contact will be undertaken with practices to encourage staff to raise awareness of the study and distribute fliers to men who meet eligibility criteria
- Local diabetic clinics and services will be approached to send letters to men in areas where GP practices are not participating. The process will be the same as that described above for GP practice invitation letters. The trial team or Clinical Research Networks will identify and approach the diabetic service; clinical staff will screen lists for eligibility criteria and the Networks will co-ordinate the sending of invitation letters linked to the HIC recruitment tracker.

ii) Community recruitment (approximately 50% of sample) will proceed according to recognised methods and good practice for community recruitment [34] designed to engage underserved and more disadvantaged populations in public health research. Community stakeholders, groups and PPI representatives will help us to identify suitable community venues which men are familiar with for recruitment and assist with information dissemination (e.g. fliers, on-line links to the study website). As in the feasibility study, community workers (public sector e.g. link workers, charities, shop keepers e.g. pharmacies, barbers, betting shops) will be asked to put up posters and have fliers at suitable venues. Fieldworkers will work in pairs to have face to face discussions about the study at information stands in community centres, local authority venues like benefits centres, garages, public spaces like libraries, supermarkets, DIY shops, public areas in health venues e.g. hospital foyers. The Fieldworkers will be GCP trained and will assess the individual capacity of participants to fully understand the purpose of the research, what participating involves, the benefits, risks and burdens.

The same Summary PIL (site headed paper) used for the initial approach in GP practices will be given to men who express interest and options for how to join the study will be discussed according to men's preference, e.g. telephone, text, web and email.

We wish to minimise contamination between the trial groups and minimise disappointment for those who are not allocated to the incentives group. Care will be taken not to recruit more than one participant from pairs or groups of men who Fieldworkers meet who are in e.g. the same workplaces, or community groups.

If recruitment is slower than expected:

- Relevant local and national websites will be approached to include the Game of Stones invitation to men e.g. exercise and weight management services, Diabetes charities.
- Community stakeholders will be asked to assist in distributing the Recruitment Poster/Fliers, for examples through an advert in local newspapers, circulars, through social media e.g. Facebook or on local radio.

iii) Full Participant Information Leaflet (PIL) and discussion with a Fieldworker. If a man is interested in participating (from the GP opt-in card or contact resulting from community outreach) the Fieldworker will provide the Summary and Full PIL (by the participants preferred method of communication) and arrange to further discuss what will be involved and answer any questions. A convenient date, time and convenient place for a meeting will be agreed (see Section 6.2 for Covid-19 procedures), with a reminder text, email or phone call (participant preference) made prior to the appointment. If the man does not attend the appointment, two methods of contact (e.g. phone, text or email) will offer another opportunity to make an appointment and join the study. This is to allow men

who are harder to reach another chance. Examples of initial non-response from our Feasibility Study and PPI included: running out of phone credit; being admitted to hospital; clashing (unpredictable) work commitment; forgot. Providing another opportunity is important to meet our aim of engaging men who are harder to reach and are underserved by weight loss support services. When recruitment commences, a study website link to an audio recording of a researcher reading out (verbatim) both the Summary and the Full PILs for those with low literacy or poor vision will be provided. The link will be available via other social media e.g. WhatsApp, Facebook.

iv) Enrolment. The Feasibility Study identified acceptable local community venues (e.g. GP surgeries, community centres, University premises, pharmacies). GP surgeries were the most popular choice of venue for assessments.

Men will be able to attend with a relative or friend, if they wish. At the enrolment appointment the Fieldworker will ensure that the man has a copy of the Full PIL, will discuss this, answer any questions and gain written informed consent. Men will be given as much time as they require to consider participation. Confidentiality of data will be explained and we will request that men do not discuss which group they are allocated to with other men participating in the study or to talk about the study on social media. This is to reduce any contamination, which could interfere with the study outcomes in this individual RCT. Participants will be informed that they can choose whether to participate or not and they can withdraw from the study at any point if they wish, without giving a reason and without any personal consequences. Completed original informed consent forms will be stored in an Investigator Site File (ISF) at each site and once randomised, the participant's trial identification (ID) number will be included. Electronic versions of all PILs and consent forms will be available, should they be required, for example to meet Covid-19 guidance.

Following informed consent, if a participant loses capacity, the consent given when capable remains legally valid. In such circumstances, a decision will be made, in conjunction with the participant and any family or carers, in relation to ongoing participation in the study.

iv) Baseline data collection: To assess reach, we will follow CONSORT guidance [35], our Feasibility study data collection [10] and systematically document recruitment routes to randomisation: the number of men personally invited by GP practices, indicating initial interest in the community and providing contact details, attending enrolment appointment, consenting to randomisation, self-reported eligibility (BMI of 30 or higher) and BMI verified to confirm whether eligible or not (see Section 5.2). Any reasons given for dropping out at any stage will be documented. The Health Informatics Centre (HIC) at the University of Dundee will host the recruitment tracker software, which adheres to NHS Safe Haven Policy to protect confidentiality. HIC have a data sharing agreement with the Centre for Healthcare Randomised Trials (CHaRT) which is a registered Clinical Trials Unit. CHaRT will provide independent

randomisation services, outcome measure databases and undertake the blinded statistical analysis. Research Fieldworkers will collect baseline data and assessments (Table 1) following the procedures described in Section 6.5. The Fieldworker will request randomisation by CHaRT using a secure remote web-based system (telephone backup).

v) Informing the participant of group allocation: After randomisation, usually at the baseline assessment appointment, the Fieldworker will inform the participant which trial group they are allocated to and ensure that they understand what will happen next. An additional specific post-randomisation PIL will be provided for each of the three intervention groups (Trial arm 1 PIL (SMS+I) together with a mock-up cheque for the incentive (see Section 7.3 for incentive detail), Trial arm 2 PIL (SMS only) and Trial arm 3 PIL (wait list) and discussed in detail with the participant. Once group allocation is known, a Fieldworker will talk through the relevant post-randomisation PIL, then complete documentation of weight target and assessment dates (on a wallet-sized Game of Stones appointment card given to the men). All men will be given a pedometer, shown how it works, and a unique login and password provided by CHaRT which will allow them to access the study website pages, appropriate for their allocated group. The Fieldworker will discuss the information and the personal web page which will show the targets, assessment dates, incentives (for SMS+I group) and the self-monitoring page. A PPI suggestion is for men to be able to upload selfie/photo to their personal password protected self-monitoring web page throughout the study, as image was considered a motivator for some men. This will be incorporated into the website re-design and will be optional, for self-monitoring and motivating purposes only. If the participant does not have access to a camera, the Fieldworker will offer to do this on the participant's behalf, using a secure Game of Stones tablet. They will delete the photo as soon as it is uploaded to the website. If the assessment is taking place remotely via video, a screen shot will be taken for men's personal web pages. The participant will have an opportunity to access the website with the Fieldworker present and ask questions to ensure that there are no operational problems. If there is no internet access, printed screenshots of the website will be available. Information about how the website works will be made available in a range of formats/media and will include Frequently Asked Questions.

vi) Future appointment preferences: The Fieldworker will ask the participant's preferred place, day and time for future appointments and inform the men of the automated appointment reminders. Target dates for future appointments will be provided. For all groups, the Fieldworker will inform the participant that they will be in contact to arrange the appointment approximately one month in advance of these dates. Participants will be asked not to disclose their study group when they attend for 12M assessments, so that a Fieldworker can remain blind and unbiased when they assess the primary outcome weight (See Section 6.4).

6.2. Strategies for addressing recruitment / assessments if Covid-19 lockdown restrictions are in place:

During Covid-19 social restrictions, a researcher verified weight assessment using a standardised method (see Section 6.6 for more details) is essential for participants to enrol in Game of Stones. Obtaining verified weights is crucial for incentive trial validity and would not be possible to undertake via a live video assessment, unless with appropriate Personal Protective Equipment (PPE). Assessments may be acceptable outdoors or in GP practice or community venues e.g. pharmacy shops, charities, local authority services who are in agreement. All other trial assessments could feasibly be undertaken using video assessments. In the event of a full Covid-19 lockdown, where face-to face verified weight assessments cannot take place, recruitment would continue, with men placed on a waiting list for enrolment once Covid-19 restrictions allow weight verification.

Recruitment: approach by letter via GP register, social media, community stakeholders (e.g. link workers, community workers), posters and newspaper adverts (the flier/poster) can continue remotely. Community recruitment approaches will be modified to ensure full compliance with current local Covid-19 restrictions which are likely to involve: situating stands outside venues, maintaining physical distancing from potential participants, use of antibacterial hand sanitiser gel/wipes and both participant (if appropriate) and Fieldworkers wearing PPE e.g. face coverings. Those interested in participating will be asked to leave their contact details either by scanning a QR code to enter details on their smartphone or by the Fieldworker noting contact details/preferred method of contact. Interested men will also be directed to the study website for further information with the web page and team contact details available in a format that is easy for them to photograph with a mobile phone. Due to potential involvement of GP practices in a Covid-19 vaccination programme, it may be necessary to increase recruitment via the community approach to avoid putting additional strain on GP practices. If vaccination is being undertaken in community venues (halls, sports centres), then researchers would request permission to have information stands. If recruitment numbers are affected by Covid-19, the research team will approach The Scottish Health Research Register (SHARE) to request access to their database of potentially eligible adults who are willing to participate in research.

Enrolment: first contact made by the Fieldworker will be made remotely using the potential participant's preferred method of contact e.g. mobile phone, landline, email, Facetime, Zoom, Microsoft Teams or Skype during which the trial will be explained further. Should individuals wish to proceed in the trial a postal address will be taken to which a participant consent form, Full PIL and baseline questionnaires will be sent electronically (paper back-up). A second appointment (preferred contact) with the Fieldworker will be made to complete the consent and baseline paperwork. The participant will be asked to initial, sign and date the consent form during the discussion. This is called "verbal confirmation of

written consent" and this will be documented by the Fieldworker. The participant will then be asked to either return the consent form by post or to bring the consent form with them to their baseline weigh-in appointment. When the consent form is returned, the member of the local site research team with delegated responsibility for consent and who conducted the consent discussion over the phone will countersign and date the consent form. The date of countersignature should not be earlier than the date of the participant signature but may be later. Randomisation can only be triggered by confirming eligibility at the baseline weigh-in appointment. Fieldworkers should not proceed with obtaining baseline weight measurement if a hard copy of the consent form has not been received in the post or given to the Fieldworker on the day of the appointment. Where verbal confirmation of written consent is not followed by receipt of a hard copy of the written consent form, the participant will not be randomised or progress further in the trial.

Baseline weigh-in: Face-to-Face baseline weigh-in (this must be conducted face-to-face, no remote option permissible) will be modified to ensure full compliance with current local Covid-19 restrictions. On the day prior to the appointment, participant and Fieldworkers will confirm that neither has any symptoms or have been in contact with Covid-19, in accordance with Government guidance (see Section 6). The weigh-in appointment is likely to involve: meeting participants outside where possible (whilst ensuring sufficient privacy), maintaining physical distancing, use of antibacterial hand sanitiser gel/wipes and both participant (if appropriate) and Fieldworker wearing PPE e.g. face coverings. The fieldworker will supply all equipment needed to comply with current guidance for both themselves and the participant. Participants will be asked to meet the Fieldworker somewhere that they feel comfortable attending, which may include their own home. If attending a participant's home, Fieldworkers should seek to conduct the weigh-in appointment in a private space outdoors where possible or in the entrance to a person's home in order to avoid contact with surfaces. Post-randomisation a wallet-sized Game of Stones appointment card and post-randomisation leaflets will be given to the men (post/email options). All equipment with hard surfaces will be wiped down before and after contact with participants with sanitising wipes.

Follow-up appointments: will be offered as above, in accordance with changing government guidance on Covid-19 restrictions. 3, 6 and 24M weigh-in appointments have a target time of around 10 minutes. The weighing will take place first and then questions about adverse events (see Section 8). At 3 and 6M appointments the SWAT protocol will guide how fieldworkers deliver the weigh-in. If face to face assessment is inadvisable or unacceptable, remote video assessment will be arranged. Short term provision of weighing scales for the assessment may be required. The 12M appointment would normally last 30 minutes (video option if in person assessment not possible). However, the face to face component could be reduced to a 10-minute weigh-in following the same protocol as the 3, 6, and 24M assessments or conducted by video. If face to face assessment is not possible, all assessments can be

done by video, apart from weight at baseline. Participant self-report questionnaires could be completed on-line, by post or by telephone with a researcher (if visual or literacy concerns). All options would be available for the 12M assessment if circumstances warranted this. Methods of data collection will be clearly documented for each assessment.

6.3. The randomisation scheme

After receiving written consent, the Fieldworker will randomise men using a secure remote web-based system provided by the CHaRT CTU, with telephone randomisation available as a backup in case of internet problems. The CHaRT randomisation service is independent of the data management and statistical team at CHaRT who will be undertaking the outcome data analysis. Randomisation will be stratified by trial centre using random permuted blocks. Participants and the Fieldworker are informed automatically by SMS about group allocation. At the same appointment, the Fieldworker then provides additional verbal and written information appropriate to the allocated group, and a unique website login and password that allows access to the web pages for that trial group. Where this takes place will be negotiated according to the local Covid-19 restrictions and participant preference. HIC recruitment tracker is linked to the group allocation to facilitate the delivery of the automated SMS and Incentive interventions. Separating the institutions involved in intervention delivery (HIC) from the randomisation service and outcome data analysis facilitates blinding of the statistical team.

Due to the low-risk and self-management nature of this public health trial, the participant's GP will not be informed of their participation in this trial.

6.4. Blinding

Primary outcome assessment will be blinded to group allocation and undertaken by a Fieldworker who has not previously met the man and/or is not aware of study group. This was tested in our Feasibility Study and found to be acceptable, understandable to participants and feasible logistically for Fieldworkers to implement. When assessments are arranged, participants will be asked not to inform the Fieldworker conducting follow-up appointments of their group allocation. Recruiting GP practices or other stakeholders who facilitate recruitment (e.g. community centre staff) will not be informed of group allocation. The statistical team analysing data will be blinded to group allocation and will not have access to data collected at 3M and 6M outcome assessments in the intervention groups during the primary analysis.

6.5. Baseline data

Demographics and measures collected at baseline are summarised in Table 1 and are based on the FFIT trial [16] and the recently published STAR-LITE core outcome set for behavioural weight management interventions [17].

6.6. Trial assessments

The methods of data collection adhere to the same protocol that was shown to be acceptable and feasible in our Feasibility Study [1, 2]. Potential modifications for various Covid-19 restrictions and scenarios are described in Section 6.2 and the schedule of assessments is summarised in Table 1. Participants will be sent a reminder of their appointment by text (or preferred contact method) the day before. Non-attenders will receive two reminders to contact the team and given the opportunity to arrange another appointment. Reminders will use different methods e.g. SMS, email, phone, post because mobile phone numbers may change, harder to reach men may run out of phone credit and some men unpredictably work away from home (zero hours contracts). Flexibility of approach is both valued by participants and required in any study aiming to address health inequalities. Relevant information about preferred methods of contact is documented by fieldworkers on recruitment tracker. In the feasibility study, some men wished to attend assessments and stay in the study but decided to stop receiving the texts. Fieldworkers will use a portable stadiometer (height) and calibrated scales (weight). Weight will be entered manually onto the Case Report Form and participants will be asked to sign to agree the weight entered . Fieldworkers will measure weight using standardised procedures and following device instruction manuals within a 3-week window of their personal weight target date in order to qualify for the linked incentive at 3, 6 and 12M. Men are requested to take off shoes, remove bulky clothing and empty pockets. Hard copies of all participant anthropometric measurements will also be noted to prevent any missing data caused by technology failure.

In the event of a face-to-face verified assessment not being possible for the primary outcome assessment at 12M (e.g. participant out of the country), every effort will be made to gain a satisfactory verified weight via video assessment, or via an independently confirmed weight by third party e.g. a pharmacist, community or health worker. Methods will be documented, to enable a sensitivity analysis to be undertaken (see Section 9.9). Besides Covid-19 restrictions, two additional scenarios where this would apply were encountered in the Feasibility Study: unexpectedly working abroad; admitted to hospital.

Self-report questionnaires (participant choice, online, paper, telephone if visual impairment or literacy issues - method documented), completed when attending weight assessments, will be uploaded to CTU databases. The questionnaires are completed by the men with the researcher present in case they require help e.g. visual or literacy issues. When completed, the researcher will check to make sure there

is no accidental missing data (e.g. two pages turned over). If missing data is noticed after the assessment appointment, the participant will be phoned as soon as possible and given an opportunity to provide the missing data. Any missing data completed after the assessment will be logged. Assessments use validated tools where available and follow quality standards for measurement procedures.

To maximise completeness of data, participants who do not attend assessments within the 3-week time window permitted, but who have not withdrawn from the trial, may be contacted (by their preferred method) and requested to complete a questionnaire (preferred method) and provide a self-report weight. This will not qualify for an incentive payment. A letter will be sent to those who don't attend the 12M assessment reminding them that they will be invited to attend an appointment at 24M.

All participants receive a £20 voucher (Amazon or High Street) reimbursement of expenses for attending the 12 and 24M assessments, consistent with evidence that financial reimbursement can improve retention [36, 37]. In the Feasibility Study data quality was high, with low levels of missing data. The completion rates for self-reported secondary outcomes were 88-99%. The Delegation of Responsibilities Log will identify all trial personnel responsible for data collection, entry, handling and managing the database.

6.7. Long-term follow-up assessments

At baseline, we will request consent for linkage to routinely collected primary and secondary care health outcomes, which was acceptable in the Feasibility Study. Linkage will not occur during the lifetime of this study. The linkage will be to obesity related health conditions documented in GP and hospital records, hospital admissions, GP practice consultations and outpatient attendances, prescribing data.

6.8. Withdrawal criteria

Participants will have a right to withdraw from the study at any time, without providing a reason. They may wish to withdraw from one or more aspects of the study (for example receiving the text messages, attending for weigh-ins, completing questionnaires, all contact from the study team, collection of routine data etc).

Participants will be informed that they can withdraw from any aspect of the study. Participants who stop the text messages can still remain in the study for follow-up. Any reason offered for their withdrawal will be documented on the CRF and a change of status form completed. Participants who withdraw will not be replaced as a 25% loss to follow-up has been accounted for in the sample size calculation. Participants who wish to withdraw from study follow-up should be asked to confirm whether they would

still be willing to consent to allow routine follow-up data to be used for trial purposes (hospital/GP medical records).

6.9. End of trial

The end of follow-up for each participant is defined as the final data capture on that individual. The end of follow-up within the trial is defined as when the final data capture on the last participant occurs. The end of the trial is defined as the end of funding. The end of the trial will be reported to the Sponsor and REC within 90 days, or 15 days if the trial is terminated prematurely. If terminated prematurely, the Investigators will inform participants and ensure that the appropriate follow up is arranged for all involved, if appropriate. A summary report of the trial will be provided to the Sponsor and REC within one year of the end of the trial. An end of trial report is also issued to the funders at the end of funding.

7. TRIAL TREATMENTS

7.1. All groups - information resource and pedometers

At baseline all men (intervention and control groups) will be provided with a website containing a menu of evidence-based information resources including standard NHS information on weight loss (printed information will be provided to those without internet access), other support websites for men (MHF,) and a low cost pedometer. Pedometers encourage self-monitoring and increase physical activity, however alone they do not result in weight loss [11]. In our Feasibility Study, a low-cost pedometer was acceptable, including with men with reduced mobility and substantial co-morbidities. It was given to counter group allocation disappointment and address inequalities: in DE compared to AB socio-economic group households 6% v 24% of adults have access to wearable technology [28].

7.2. Control group

Waiting list for 12 months followed by 3 months of SMS (months 13-15 inclusive). At baseline, all groups will receive information (web page; written option) about weight loss and be given a pedometer. There are no interim measurements (at 3 and 6M). Men can choose whether to try to lose weight or not during the 12 months. The control arm is as close as possible to “doing nothing”, whilst remaining ethical by providing information, free choice to lose weight and later assistance via SMS to reduce disappointment bias. This control group design was decided through PPI consultation and qualitative research in the Feasibility Study and was acceptable to most men (only one man withdrew due to allocation disappointment). Men are assessed for the same outcome measures as the other groups at 12M and 24M only. No weight loss targets are set and men do not have access to a self-monitoring page.

7.3. Interventions

The interventions are described following the Template for Intervention Description and Replication (TIDieR) guidance [38]. They apply the principles of self-management, are designed to be efficient, and minimise burden on NHS weight management service staffing resources.

7.3.1 SMS

Why-theory: The SMS were designed in collaboration with nine men from the University of Stirling Public Engagement Group and Men’s Health Forum and informed by: evidence based Behavioural Change Techniques (BCTs); systematic review of effective SMS studies [12] and analysis of qualitative data on acceptability and men’s preferences in the Feasibility Study. They were written with input from the editorial and creative consultant of Men’s Health Forum GB who has provided award-winning health guides and online material for men on weight and other health issues [26, 39]. SMS for 12M are

embedded with: evidence and theory-based BCTs based on the Health Action Process Approach [40], Self-Determination Theory [41] and the Behaviour Change Maintenance Model [42]. Daily SMS target automatic processes by acting as a consistent prompt and cue reminding participants of their intention to lose weight, thus aiming to reduce impulsive behaviours [43]. The following feedback from men in the target population was incorporated: include concrete tips, facts and links to information websites; include a range of perspectives and approaches as no one approach will suit everyone all the time; introduce engagement elements such as questions and prompting reflections. Men liked texts to seem as if they were coming from other men taking part in Game of Stones (*“like getting texts from your mates”)*

Why-intervention components: The SMS include BCTs which target motivation, self-regulation and maintenance processes, underpinned by a person centred-approach [44]. Some texts are derived from qualitative interview data from the Feasibility Study where men have told us what they find helpful. Engagement is encouraged by asking direct and rhetorical questions on weight management and employing general communication techniques such as agenda setting or use of humour. This is supported by qualitative data from our Feasibility Study and a qualitative evidence synthesis on obesity in men [45]. Men like factual information and banter, and dislike strict diets and being told what to do. Texts contain facts about losing weight: eating less, reducing portion size, healthy snacks, sugar and alcohol, sticking to a plan and increasing physical activity and exercise. Texts embed links to relevant websites for more information and self-monitoring sites (e.g. weight, step count). Information resources include: e.g. NHS choices, the Eatwell Guide; Man v Fat; Men’s Health Forum. How participants act will depend on personal food preferences; gradual and achievable changes to their normal diet and activity will be suggested in line with recommendations [2]. The sequencing of SMS covers weight loss (until 6M), followed by more weight loss maintenance topics (months 6-12). Individual SMS are non-consecutive and stand-alone: they do not require paying attention to, or engaging with previous content. After each weight assessment at 3, 6 and 12M, men receive a personalised SMS including the participant’s name and weight change.

What-materials: In addition to the SMS, participants are provided with a web-page (written information if no internet access) offering a choice of evidence-based weight management and physical activity approaches plus a pedometer to operationalise the self-monitoring BCTs in SMS.

What-procedures: Single SMS are sent at a default rate of 1 message per day between 9:00AM and 8:30PM, with options to reduce the number, based on Feasibility Study findings.

Who provides: The SMS intervention is delivered by the Health Informatics Centre (HIC) at the University of Dundee via tried and tested technology used in the Feasibility Study and other RCTs [31-33]. The intervention package is designed so that the SMS library can be readily delivered by other SMS providers to facilitate future roll out by the NHS or public services. To address our objective: to refine

the digital programming for future scalability and implementation, CHaRT will investigate further integration planning for Phase 3 of Game of Stones. Collaborators at the Digital Health Institute (DHI) who are funded by Scottish Government to progress digital innovation within the NHS will provide access to their sandbox early 2021. A sandbox is a piece of software that can access only certain resources, programs, and files within a computer system. CHaRT will explore the digital capabilities and infrastructure that is currently available to ensure that the delivery systems are future proofed as far as they can be for future implementation.

***How (mode of delivery)*:** Remote delivery. SMS are sent by the HIC automated system.

Where: SMS can be received anywhere depending on access and read at any time. Men require a mobile phone (any type, not just a smart phone) to maximise reach.

When and how much: Daily texts arrive at varying times between 9am and 8.30pm (preference for am or pm can be accommodated e.g. for men who work night shifts). Men can reduce the SMS frequency from 1 per day to 3 or 5 per week or increase it again to 7 per week (maximum). SMS can be stopped and restarted by a simple instruction e.g. "STOP TEXTS" and "RESTART". Men can stop the SMS but remain in the trial for assessments and follow up if they wish.

7.3.2 Intervention 2: endowment incentive adjunct to SMS

Why-theory: The novel endowment incentive for verified weight loss in addition to the SMS is based on theory [46, 47] and evidence [13-15, 48-50]. In the Feasibility Study, the incentive structure was decided by considering evidence from multiple sources: a Discrete Choice Experiment (DCE) completed by 1045 men in the target population; extensive PPI; systematic review evidence; psychological and economic theory and analysis of qualitative interview data [2]. The incentive draws on behavioural economics [46, 47] which shows that people ascribe more value to something because it belongs to them (endowment effect) and are more motivated to avoid losses than they are to achieve similarly sized gains (loss aversion).

Why-intervention components: The incentives are offered alongside the SMS because systematic reviews consistently report that financial incentives alone are unlikely to be effective for sustained complex behaviour change, without additional behaviour change strategies [14, 48, 51].

***What-materials*:** All participants are 'endowed' with the financial incentive at the start of the trial. Money can only be accessed at 12M (no withdrawals) if weight loss targets are met. It is placed into a hypothetical personal account (held by the incentive provider) and a mock-up cheque, printed on high quality paper, is given to men at the start to encourage participants to feel ownership of the money.

What-procedures: Men receive the full incentive at 12M if they achieve all 3 weight loss targets, but they 'lose' money if targets are not met. Detail is described elsewhere [2]. The top level weight loss

target suggested by NICE is 10% [9] and this was endorsed by PPI. This is more ambitious than the 3% mean weight loss in weight management programmes quoted in NICE, current systematic review evidence for men with obesity [11] and the 5% weight loss at 12M in the FFIT trial [16]. The full incentive can be secured by meeting all verified weight loss targets: 5% of body weight lost (from baseline) at 3M, 10% lost at 6M and 10% lost at 12M. At 6 and 12M, some money for each % weight loss not attained between 5-10% is lost. Weight must be objectively measured by a Fieldworker (see Section 6.2 for Covid-19 procedures) within 3 weeks of the target date. The incentive is calculated automatically when verified weight is entered into the centralised database. Men then receive a personalised SMS with the amount of incentive secured/lost, are encouraged to keep trying as they can still secure money and this is also presented on their personal private SMS+I webpage.

All men are given a wallet-sized Game of Stones appointment card with their personalised target weights for each appointment date and Fieldworkers fill in objectively measured weight at each assessment.

Who provides**:** Incentives are provided as part of this NIHR research grant and are triggered by achieving target weight loss, verified by the Fieldworker. Feedback on weight achievement and incentive status will be through SMS and a study webpage.

How (mode of delivery): Men receive an automatic bank transfer of money to their bank account (BACS) once the automated database confirms the amount at the 12-month assessment. In the Feasibility Study, all participants had a bank account, however secure postal payment methods will be available if required.

Where*:* Weight goal verification is undertaken by Fieldworkers at places convenient for men, usually a health or community centre (See Section 6.2 for Covid-19 procedures). Entering participant weight onto the database triggers an automatic SMS notification.

When: At 12M men receive the money depending on weight loss achievements [2]. If weight at 12M exceeds baseline weight, no payment is given, regardless of whether interim weight loss targets were met, as recommended by participants in the Feasibility Study and PPI.

7.4. Compliance with interventions

Both interventions are automated and centrally delivered linked to the HIC recruitment tracker, so fidelity of delivery is not an issue. Engagement with the interventions (SMS received from participants; Game of Stones website access data, self-report items in questionnaire at 12M), retention and any reasons offered for drop-out will be documented.

8. ADVERSE EVENTS

8.1. Standard definitions

Table 2: Definitions of adverse events

Term	Definition
**Adverse Event (AE)**	Any untoward occurrence in a participant to whom the intervention has been administered, including occurrences which are not necessarily caused by or related to the event.
**Serious Adverse Event (SAE)**	A serious adverse event is any untoward occurrence that:<br>• results in death<br>• is life-threatening<br>• requires inpatient hospitalisation or prolongation of existing hospitalisation<br>• results in persistent or significant disability/incapacity<br>• is otherwise considered medically significant by the investigator<br>NOTE: The term "life-threatening" in the definition of "serious" refers to an event in which the participant was at risk of death at the time of the event; it does not refer to an event which hypothetically might have caused death if it were more severe.

8.2. Operational definitions for this study

In this trial:

- An adverse event is defined as any injury or newly diagnosed health condition (e.g. diabetes mellitus, cancer, etc) that occurred while a man was participating in Game of Stones, in any of the groups, whether or not this was related to his participation in Game of Stones.
- AEs will be recorded by Fieldworkers within the CRF from the time a participant consents to join the study until the end of the 24-month follow-up period.
- Hospitalisations for treatment planned prior to randomisation and routine hospitalisation for elective treatment will **not** be considered or recorded or reported as an AE or an SAE. Complications occurring during such hospitalisation will also not be considered, recorded or reported as an AE or an SAE.
- Any death that occurs during the trial is very unlikely to be related, but all deaths will be documented as AE and relatedness determined.

8.3. Detecting AEs and SAEs

At each contact with a participant (3, 6, 12 and 24M), the Fieldworker will ask about the occurrence of AEs/SAEs. Open-ended and non-leading verbal questioning of the participant will be used to enquire about AE/SAE occurrence. If any AE is likely to meet the criteria for an SAE, as much detail as possible about the event will be sought from the participant. This a community-based study, and the Fieldworker and PI will not have access to hospital or GP medical records, so the only source of information about the event is likely to be from the participant themselves. AEs/SAEs will be recorded, evaluated, reviewed and reported as described below.

In addition, the research team may be alerted to a possible AE/SAE via screening of text message responses (see Section 8.8 on Safeguarding), or by direct contact with the trial office. If the AE/SAE is confirmed, this will be recorded, evaluated, reviewed and reported as described below.

8.4. Recording and assessing AEs and SAEs

AEs will be recorded within the case report form and entered onto the study website. All relevant information should be captured on the case report form. The information should be entered onto the study website within 72 hours (3 days) of the event being identified.

The Principal Investigator (or delegate) for each site will review the AEs and make an evaluation of seriousness, causality and expectedness and record this within the case report form. The evaluation of AEs should be done and entered onto the study website within 7 days of the event being identified by the field worker.

Assessment of Seriousness

An assessment of seriousness, as defined in Table 2 above will be made.

Assessment of Causality (relatedness)

- Related: resulted from administration of any of the research procedures or to the man's effort to lose weight
- Unrelated: where an event is not considered to be related to any of the research procedures or to the man's effort to lose weight.

Alternative causes such as natural history of the underlying disease (obesity; co-morbidities), concomitant therapy, other risk factors and the temporal relationship of the event to the treatment will be considered.

Assessment of Expectedness

If the event has been assessed as being related to the administration of any of the research procedures or to the man's effort to lose weight, an assessment of expectedness is required. The following events are expected:

- Musculoskeletal injuries or musculoskeletal pain
- Cardiovascular events (such as angina, myocardial infarction, palpitations, etc)
- Nutritional deficiencies
- Gall stones, newly manifest or worsening of known symptoms
- Changes in bowel habits
- Negative impacts on mental health (such as depression, fatigue, suicidal thoughts, etc)
- Low blood sugar, changes in diabetic medication, diabetic complications

All other events are considered unexpected.

8.5. Review of AEs and SAEs

The Chief Investigator or medically qualified delegate will review the following categories of AE within 72 hours (3 days) of the PI assessment:

- Serious adverse events that have been assessed by PI as related and not expected. {*These SAEs require to be reported to the REC and should have medical oversight and input as the report is prepared*)
- Serious adverse events that have been assessed by PI as not related. (*We recommend medical oversight on these events to confirm that they are not related*)

Serious adverse events that have been assessed by the PI as related and expected will be collated and reviewed by the PMG and TSC at their meetings – they do not need real-time review by the CI or medically qualified delegate. Likewise, non-serious adverse events will be collated and reviewed by the PMG and TSC at their meetings. The PMG and TSC will be blind to treatment allocation when reviewing SAEs and AEs. The independent TSC will also have the role of a Data Monitoring Committee to provide oversight of safety within the trial.

8.6. Reporting AEs and SAEs to REC

If, in the opinion of both the PIs and the CI, the event is confirmed as being serious and related and unexpected, the CI or Trial Manager will notify the Sponsor within 24 hours of the CI (or medically qualified delegate) confirmation. The Sponsor will provide an assessment of the SAE. A Sponsor cannot downgrade an assessment from the PI or CI. Any disparity will be resolved by further discussion between these parties. The CI or delegate will report any related and unexpected SAEs to the REC within 15 days of the event being identified.

If all the required information is not available at the time of reporting, the Fieldworker must ensure that any missing information is provided as soon as this becomes available. It should be indicated on the report that this information is follow-up information of a previously reported event.

All related SAEs will be summarised and reported to the Research Ethics Committee, the Funder and the Trial Steering Committee in their regular progress reports.

8.7. Responsibilities

Table 3. Responsibilities for collecting data on adverse events

Fieldworkers	• Ask about the occurrence of AEs/SAEs<br>• Record information about AEs on the CRF and enter this information onto the study website within 72 hours (3 days) of the event being identified*
PI or delegate	• Review and assess AEs, and ensure this information is recorded on the study website within 7 days of the event being identified
Trial managers	• Ensure that relevant SAEs are reviewed by the CI (or medically qualified delegate)<br>• Report SAEs that are related and unexpected to REC<br>• Collate summaries of AE and SAEs for PMG and TSC meetings
CI (or medically qualified delegate)	• Review relevant SAEs within 72 hours (3 days) of the PI assessment

* Any member of the research team have this responsibility if they are the first to receive information about an AE.

8.8. Safeguarding

In the text message intervention groups, men may reply to the text messages being delivered. The Health Informatics Centre in Dundee have experience of automatically screening incoming text messages from participants in similar studies[31-33] for words like ‘suicide’, ‘die’, ‘death’, ‘help’. The trial will use this screening process.

In the intervention groups, if a text message from a participant contains a keyword (e.g. help, suicide, death, die) the message is given high importance. Monitoring will run for the duration of the trial assessment period (24M). The HIC monitoring service will send the message in an email to appropriate researchers (the site Fieldworkers and the Trial Managers), providing the participant ID number and content of the text message.

If the research team is concerned regarding the content of a message, they will discuss this with a senior colleague (CI or medically qualified delegate). Similarly, if any issues are raised during any contact with a participant that cause concern (for example if a participant tells a Fieldworker that he feels suicidal), this will be discussed with the PI, and then CI or medically qualified delegate. In these circumstances, the allocation can be unblinded if necessary, and a course of action will be agreed. If the safety of the participant or of another person was thought to be at risk, a member of the research team will alert appropriate services: informed consent is sought for this when men join the study.

Patient information leaflets and information available on the GoS website will provide local and national contacts where men can access support.

9. STATISTICS AND DATA ANALYSIS

A Statistical Analysis Plan (SAP) will be produced separately by the trial statisticians. A separate SAP will be prepared for the 24M outcome data after TSC approval to proceed to 24M data collection.

9.1. Sample size calculation

We will require to follow up 146 men in each group to detect differences in weight loss between groups of at least 3% at 12M, with 90% power and two-sided alpha equal to 2.5% (to maintain a nominal significance level of 5% with two tests being used). With an expected 25% loss to follow-up as observed in the Feasibility Study at one year, a total of 585 men will need to be randomised: 195 per trial group and 195 (65 per trial group) at each of the three centres.

The sample size calculation is based on detecting a mean difference in weight between intervention groups and control of at least 3.3kg, assuming a pooled standard deviation of 8kg. A minimum clinically important weight loss of 3% is recommended by NICE [9], and the mean difference of 3.3kg is derived from 3% of 109kg (the mean baseline weight in the Feasibility Study). Several trials of SMS-delivered weight management interventions [12] reported an effect size >3.3kg, including the largest study which was the only trial to include predominantly men, suggesting 3.3kg is an achievable mean weight loss. The standard deviation for absolute weight loss ranged from 4.9kg to 6.3kg in the Feasibility Study (at 12M) and from 2.5kg to 7.3kg in the systematic review. We therefore conservatively assume a standard deviation of 8kg.

9.2. Planned recruitment rate

The estimated randomisation rate for men recruited is 25/centre/month over 8 months. This is based on Feasibility Study data: Community recruitment took on average took 2.3 recruiter hours per participant randomised and GP letters had an opt-in rate of 10.2% (n=90/879). Recruitment will be flexible, including at weekends and in the evenings, enabling men who work to join the study. There are three recruiting centres covering very wide urban, suburban and more rural areas which will provide diversity and many potential venues for recruitment. GP Practices will range in the size/percentage of the population meeting eligibility criteria, and the expected consent rate.

In our Feasibility Study we proposed to use adverts via local newspapers, circulars and social media in collaboration with local community stakeholders, if recruitment rates were lower than anticipated. They were not required as more men expressed interest in joining than could be accommodated. These remain in the current protocol, as they are more Covid-19 resilient recruitment methods than manned stands in community venues.

9.3. Statistical analysis plan

A single analysis will be conducted once primary outcome data collection is complete, following a pre-specified Statistical Analysis Plan developed according to recently published guidelines [52]. The

analysis will be undertaken blind to group allocation by a statistician at CHaRT who will not have access to the HIC verified weight data at 3 and 6M which are part of the intervention. The HIC dataset for subsequent analyses, will be provided after the primary analysis has been completed. This solves the blinding issue in our Feasibility Study [2] due to different group assessment schedules (3 and 6M assessments for the intervention groups only).

All outcome measures will be summarised with the appropriate descriptive statistics where relevant: mean and within-participant change in weight from baseline to 12 months expressed as a percentage of baseline weight, mean and standard deviation for continuous outcomes (or medians and interquartile range for skewed data) and counts and percentages for dichotomous and categorical outcomes.

The primary analysis will follow CONSORT reporting guidance and will be by intention to treat using multiple imputation of missing outcome data. Analysis to account for non-compliance is not necessary, as automated interventions can only be accessed via randomisation, therefore cross-over cannot occur and contamination was minimal in the Feasibility Study. This is a self-management intervention, therefore stopping the text messages but continuing to attend weight assessments is compliant.

Exploratory sub-group analyses are hypothesis generating to inform future research and implementation and will be reported separately after the primary outcome analysis is complete.

9.3.1 Summary of baseline data and flow of patients

The variables used to describe our sample are consistent with those reported in the Feasibility Study [1]: age, weight, height, BMI, deprivation category, partnership status, co-morbidities, ethnic group, education, working status, household size, weight loss history and strategies used, physical activity and sedentary behaviour, alcohol frequency, smoking status, confidence in ability to lose weight and to maintain weight loss, EQ-5D-5L and the Warwick-Edinburgh mental well-being scale (WEMWBS). Definitions and rules for derivation of these measures were set out in the statistical analysis plan for the Feasibility Study and will be incorporated into the SAP for the full trial. New variables include disability and access to self-monitoring equipment (scales, activity tracker). “Perceived wealth”[23] and “Financial Strain”[24, 25] are included to capture additional health inequalities data. Baseline data for the additional exploratory research for men living with obesity and mental health conditions and/or MLTC are: MLTC with or without diabetes.

A CONSORT flow diagram will be produced similar to the report of the Feasibility Study [1].

9.3.2 Primary outcome analysis

The analysis of the primary outcome will estimate the mean difference in change from baseline weight expressed as a percentage at 12M between groups (SMS+I vs control; and SMS only vs control),

using a linear regression model adjusting for minimisation variables, with recruitment centre as a fixed effect.

9.3.3 Secondary outcome analysis

Secondary and exploratory outcome measures will be analysed similarly, using an appropriate generalised linear model, including binary logit regression for dichotomous outcomes (e.g. smoking status) and ordered logit for ordinal outcomes (e.g. alcohol intake). Statistical significance will be at the 2.5% level, consistent with the assumptions made in the sample size calculation. Descriptive summaries of process outcomes will be reported but no formal analysis of these data will be undertaken.

9.4. Subgroup analyses

Compliant with CHAMP reporting guidelines, pre-specified exploratory subgroup analyses for moderators will examine effects of socio-economic status (IMD, perceived wealth; financial strain; education; work status; social weight loss; household and relationship status); mental health and wellbeing (EQ-5D-5L; anxiety and depression dimension of EQ-5D-5L; WEMWBS, disability, a self-report mental health condition, PHQ-4, WSSQ, lives alone, alcohol frequency, possible latent mental health condition and obesity related comorbidity [53], MLTC, presence or absence of diabetes as one of the MLTC); other effects (satisfaction; confidence). See section 4.4.3 and the statistical analysis plan.

The NIHR Funding Board asked for subgroup analysis of outcomes by recruitment centre, strategy (community vs GP) and deprivation category (based on the postcode address where participants live – UK adjusted IMD – e.g. most deprived two quintiles v least three deprived quintiles). Data from the separate IMDs in England, Scotland and Northern Ireland will be combined to account for differences in their methodologies [54]. The context and places where men are recruited will be reported descriptively, removing any identifiable data to maximise protection of participant anonymity, as we did in the Feasibility Study. In the Feasibility Study, participants recruited via GP obesity registers were more likely to live in deprived communities, were older (M=57.1 vs. 48.3 years), more likely to report having a co-morbidity (87% vs. 44%) and had lower BMI (35.0 kg/m^2 vs. 36.2 kg/m^2) compared to community recruits. In addition, there were different 12-month retention rates: (80% GP vs. 71% community), hence we will investigate these differences. Subgroup by treatment group interactions will be tested and all exploratory sub-group and mixed methods analyses will be reported separately from the primary outcome analysis.

9.5. Interim analysis and criteria for the premature termination of the trial

This is an automated self-care intervention and there are unlikely to be any safety concerns (there were none in the Feasibility Study) and neither is cross-over a concern as the intervention delivery is automated. Given the low-risk nature of the intervention, it has been agreed with the Sponsor that an independent data monitoring committee will not be required and therefore no interim analyses will be conducted.

9.6. Participant population

The participant population for statistical analysis is all participants randomised (intention to treat).

9.7. Procedure(s) to account for missing or spurious data

For all baseline characteristics and outcome measures, we will report the level of missing data. When estimating treatment effects, if a relevant baseline score is missing, we will implement best practice by imputing the score (if continuous) or adding an extra category for missing (if categorical). The primary analysis will use multiple imputation of missing outcome data, applying predictive mean matching. Sensitivity analyses of the primary outcome will examine the data under various assumptions around missingness, including an analysis of observed cases only, in addition to missing weight data being treated as Baseline Observation Carried Forward (BOCF) (which in our 12-month assessment is the same as a Last Observation Carried Forward (LOCF)), as recommended in the STAR-LITE core outcome set [17], for comparability with previous weight loss studies [55].

Data quality assurance and source data verification will be carried out in accordance with the CTU's standard operating procedures in order to minimise spurious data. Further data quality checks will be carried out by the trial statistician prior to the analysis and potentially implausible data will be queried with trial office and/or site staff.

If a data item includes the option "Prefer not to say", any such responses will be treated as a separate category and not classed as missing data.

9.8. Other statistical considerations.

The final approved version of the Statistical Analysis Plan will be publicly available at the end of the trial. This will be accompanied by a summary of changes made from the original version and the final approved version. We will also report any aspects of the analysis which have deviated from the SAP along with a justification for the deviation.

One other consideration is that the TSC in discussion with NIHR may consider other core outcome criteria in addition to the primary outcome when deciding about progression to Phase 3. This will include the following secondary outcome measures: absolute weight change (kg); % of participants achieving ≥ 5% weight loss; % of participants achieving ≥ 10% weight at 12M from baseline.

9.9. Economic evaluation

A full economic evaluation will be conducted. This will include a trial-based analysis and a decision model to assess economic value over an extrapolated lifetime horizon. The primary economic outcome

will be incremental cost per Quality Adjusted Life Year (QALY). A Cost Effectiveness analysis (incremental cost per % weight loss) will also be performed. Firstly, these analyses will be performed using the 12M follow-up data, then updated if the study progresses to Phase 3 (24M follow-up).

***Estimation of costs**:* The resources associated with the interventions will be measured using study-specific forms. Other NHS resource-use (GP, nurse, emergency department, outpatient appointments and inpatient stays) will be collected via participant reported questionnaires at baseline, 12 and 24M. The costs of NHS/Public Health funded weight loss services will also be included. Resource use estimates will be combined with unit costs obtained from standard sources or study specific estimates. Incremental costs (NHS perspective) will be estimated using generalised linear models with appropriate distributions for cost data and adjustment for baseline covariates.

Estimation of benefits: We will measure benefits in terms of QALYs gained, based on participant responses to the generic EQ-5D-5l health related quality of life measure at baseline, 12 and 24M if the study progresses to Phase 3. Incremental QALYs will be estimated using generalised linear models with adjustment for baseline covariates.

Trial based economic evaluation: Costs and benefits will be combined to estimate cost per QALY gained over the 12M follow up period. A full incremental analysis will be performed. A cost per % weight loss will also be estimated. Deterministic sensitivity analyses will be undertaken to test the impact of assumptions and analysis methods on results. Results will be plotted on cost-effectiveness planes to illustrate the impact of sampling uncertainty on results. The analysis will be updated if the study progresses to Phase 3.

Longer term cost-effectiveness: The trial results at 12M will be extrapolated over a life-time horizon using the PRIMEtime-CE-obesity model [56]. This is a proportional, multistate life table population model which links BMI to mortality and a range of diseases including diabetes, coronary heart disease, stroke and cancer. Different assumptions of weight regain will be explored. Differences in Costs and QALYs will be presented for different time horizons (this will include estimates of any cost savings in NHS health care utilisation in the short, medium and long term) although we note that lifetime horizon is the most appropriate for decision making. Sensitivity analyses will explore the impact of other key assumptions on cost-effectiveness. A probabilistic sensitivity analysis and various threshold and scenario analyses will be conducted. Results will be presented on the cost-effectiveness plane and cost-effectiveness acceptability curves used to illustrate the probability that SMS+I and SMS groups are associated with positive or negative incremental net benefit. The model will be updated if the study progresses to phase three.

9.10. Mixed method process evaluation:

A theory-driven process evaluation will be informed by MRC Guidance [57] and the CICI Framework for implementation [58] and adds value to the extensive mixed method process evaluation undertaken in the feasibility study. It will seek to contextualise and understand trial participant experiences and behaviours and explore issues to inform future implementation. The desirability of social weight loss (trying to lose weight with others) was expressed by some participants in the Feasibility Study, whereas other participants preferred to try by themselves. This will be explored together with perspectives on living with obesity, mental health conditions and/or multiple long-term conditions to inform future research, possible intervention tailoring and implementation.

Recruitment & Informed Consent:

As in the Feasibility Study, participants at trial enrolment will be asked whether they agree to a researcher contacting them at 12 and/or 24M for a telephone interview (remote meeting e.g. Teams may be preferred). This facilitates maximum diversity sampling informed by baseline socio-demographic characteristics and deviant cases (e.g. stop receiving the interventions but stay in the trial). 8-12 men will be interviewed with diverse experiences of living with obesity and mental health conditions. 8-12 men will be interviewed who self-report multiple long-term conditions at baseline. We aim to interview 10-18 men per trial centre in total; at 12M (<54 men), and/ or at 24M (estimate 21 men retained; see Table 1). At first contact prior to the interview (participant preference - SMS, Phone, Email), the researcher will provide information about the interview, give the participant as much time as they require to ask questions and consider participating, and then arrange a mutually convenient time for an interview. In order to ensure that prospective interviewees have at least 48 hours to consider the information, consent will be taken at the beginning of the interview and will be audio recorded. Anonymity and confidentiality for reporting will be ensured for trial participants.

Stakeholders (n=12-18) will be identified through our TSC, Co-Investigator and Collaborator networks. Since stakeholder interviewees may potentially be identifiable by the nature of their roles, care will be taken to ensure that any quotes drawn from these interviews will not be attributable. Processes of informed consent will include: email contact with information sheets and a request to take part in an interview; to be followed up by a telephone/remote call to arrange a suitable time/date for interview and provide an opportunity to answer any questions about the study. Participants will be asked to read the Stakeholder Information Leaflet and at the beginning of the interview consent will be verified and audio-recorded so that all interview consent processes are recorded in the same format.

Data Collection

The qualitative Research Fellow based at Stirling will interview men she has not met (i.e. participants in Bristol and Belfast), to reduce the risk of socially desirable responses. Researchers at Bristol and Belfast will interview men participating in Scotland. The qualitative research aims to conduct semi-structured telephone/remote interviews with: i) participants across the three study sites at two time points (12 and 24M) to understand experiences and behaviours of trial participants and barriers/facilitators to longer term sustained weight loss; and ii) commissioner and stakeholders in Phases 1 and 3 to explore contexts to inform scalability and implementation. Topic guides will inform discussions. All qualitative processes from sampling through to data analysis will follow an iterative process throughout the study, with emerging themes informing subsequent data collection and analysis.

Telephone/remote interviews are highly cost-effective and have been shown to be acceptable to men in other studies [59]; some find them more convenient and less anxiety provoking when discussing potentially sensitive issues around weight. Participant preferences will be sought, and face to face may be appropriate e.g. for men with audio-visual or technology access concerns. Interviews will be audio-recorded, transcribed in house or by an agency that is GDPR compliant and has signed a confidentiality agreement, and downloaded for analysis into QSR NVivo (v12) software. Interviews with men will last between 30-60 mins, depending on what they wish to talk about, and stakeholder interviews will last around 30 minutes. If Covid-19 restrictions allow and according to participant wishes, some of these interviews may occur face-to-face,

Contexts to inform scalability and implementation will be informed by three approaches. Firstly, a purposive sample of weight assessment appointments at 3m (n=40) and 6m (n=40) will be audio-recorded to check fidelity to trial processes. Secondly, the research team will keep fieldnotes about trial processes, features of weight management services, and context relevant to future implementation and scalability. Finally, the qualitative RF will interview 6-8 stakeholders by telephone/remote e.g. Primary Care and Public Health Commissioners in Phase 1, and 6-8 different stakeholders in Phase 3 (sampling informed by Phase 1 data analysis) to explore future implementation issues.

Qualitative data analysis: interviews will be transcribed and uploaded into QSR NVivo (v12) along with fieldnotes and audio recordings of researcher-participant interaction. Two researchers will independently develop a coding frame (agreed through team discussion) and identify key themes after reading a diverse sample of interviews. Analysis will explore and refine a hypothesised theory of change, guided by the five stages of the Framework approach [60]: familiarisation; identifying a thematic framework; indexing; charting; mapping and interpretation. Attributes (for trial participants) will be assigned for socio-demographic data (e.g. postcode IMD; ethnicity; age; occupation; comorbidities; MLTC; mental health conditions, possible latent mental health conditions) and these will added to interview transcripts. After data-lock, outcome data (e.g. weight loss; wellbeing) will also be assigned

attributes and variables added to the transcripts. Matrices will be constructed using the matrix coding query feature in NVivo to set further attributes (e.g. trial arm, weight loss trajectory, IMD, sub-group analysis variables - see Section 4.4.3 and section 9.4) to identify patterns with the aim of contextualising/interpreting participant outcomes. Analysis of scalability data will generate themes to inform future implementation and sustainability. The full multidisciplinary team with PPI will contribute their expertise to data interpretation.

Descriptive summaries of process outcomes will be reported but no formal analysis of these data will be undertaken. When reporting qualitative data findings, any quotations or data will be anonymised and/or non-attributable so that participants cannot be recognised.

9.11. Nested Study Within A Trial (SWAT)

A nested SWAT (Study Within A Trial) RCT is included, which trial funders and the sponsor have approved as a PhD studentship. The SWAT summary was registered on the SWAT website in August 2021:
https://www.qub.ac.uk/sites/TheNorthernIrelandNetworkforTrialsMethodologyResearch/FileStore/Filetoupload,1165876,en.pdf

The SWAT will explore the effect of researcher-participant contact, between recruitment and primary outcome data collection, on retention within the main trial. Two protocol-driven weight assessments reflective of varying practice within primary care will be delivered to all intervention participants: a "minimal contact" weight assessment (i.e. a 5 minute interaction to mimic digital self-care only) compared with an "active listening" weight assessment (i.e. a 10 minute interaction reflective of consultations in primary care) informed by the core conditions of a person-centred approach (empathy, openness, unconditional positive regard) and the recent literature on behaviour change in weight management [11, 45, 61].

The SWAT draws on data from the intervention participants and therefore will adhere to trial and process evaluation conduct (including consent, drawing on the same information leaflets and consent forms) reported in full above. Only key additions/differences are reported below.

SWAT Randomisation and Consent:

Randomisation for the nested SWAT will follow the same process as the main trial i.e. the fieldworker will use a secure remote web-based system provided by the CHaRT CTU. Participants randomised to the intervention groups (SMS+I and SMS) of the main trial will immediately be randomised again to receive one of two protocolised weight assessment approaches ("minimal contact" group or "active listening" group) using random permuted blocks to ensure balance between the two groups . Randomisation will be stratified by the main trial intervention group and trial centre.

Participants will be informed within the Full PIL that they will be allocated by chance to receive one of two weight assessment approaches, lasting either around 5 minutes or 10 minutes. They will be reminded of this process when providing informed consent.

Fieldworkers will also be required to provide informed consent (SWAT fieldworker consent form) prior to baseline data collection for audio-recording a sample of interactions with participants and for interviews with the SWAT researcher as per the qualitative processes above.

Data Collection

Drawing on the main trial and qualitative process evaluation, the SWAT will also gather additional data in order to investigate the researcher-participant relationship and retention.

Participant data:

Participants will complete the self-report Weight Self-Stigma Questionnaire (WSSQ) [62] at baseline and 12 months and the Consultation and Relational Empathy (CARE) measure at 12 months [63].

Research Fieldworker data:

Fieldworkers will complete two self-report measures, weight stigma (e.g. the Fat Phobia Scale-Short Form [64] and empathy (e.g. Questionnaire of Cognitive and Affective Empathy [65]) at baseline and 12 months. Fieldworkers will also complete a relationship measure such as the WAIS-SR (helper version) [66] directly after weigh-in assessments with trial participants.

Qualitative Analysis

Qualitative data collection and analysis is as reported in the process evaluation section above. It will not involve separate interviews, but rather will conduct additional analysis on discrete questions posed in the qualitative topic guide. A sub-group analysis of participants in the trial intervention arms (n=292) will explore the experience and acceptability of the weight assessment approaches at 3 and 6M and their relationship with the researcher. Separate matrices will be constructed to consider weight assessment group and retention to identify patterns. Once known the secondary outcomes: weight bias, empathy and relationship will be incorporated into the interpretation to understand participant, fieldworker and weight assessment group characteristics and their impact on retention. The data from this part of the interview will therefore be analysed and reported separately.

Interviews will also be undertaken with fieldworkers at each of the sites to understand their experience of delivering the protocolised weight assessments at 3 and 6M. These interviews will last around 30 minutes and will be guided by a separate topic guide.

In addition to the qualitative data analysis 4-5 audio-recordings from each of the fieldworkers at the three sites will be analysed in NVivo and used to assess fieldworker fidelity to the 2 protocolised weight assessments at 3 and 6M.

Quantitative analysis:

The SWAT will draw on the main trial and process data outlined above. The analysis for the SWAT will be undertaken separately from the main trial, overseen by the trial statisticians, and will include a CONSORT flow diagram. The dataset will be provided by the Health Informatics Centre, University of Dundee and CHaRT for analyses.

The primary outcome will be retention rate at 12 months. The secondary outcomes will be participant-researcher relationship and weight stigma.

The primary analysis will be a comparison of randomised groups using generalised linear models.

The variables used to describe the sample will include: age, weight, height, BMI, deprivation, partnership status, comorbidities, ethnic group, education, working status, household size, empathy, relationship and weight stigma. Variables and outcomes measures will be summarised with the appropriate descriptive statistics where relevant: mean and SD for continuous outcomes and counts and percentages for dichotomous and categorical outcomes.

An analysis plan, separate from the main trial statistical analysis plan, will be developed to describe in detail both the quantitative and qualitative analyses for the SWAT.

10. DATA MANAGEMENT

10.1. Data collection tools and source document identification

Case report forms

The primary outcome is assessed using calibrated scales (weight) all participant anthropometric measurements will be noted on electronic and hard versions of the CRF to prevent any missing data caused by technology failure. The hard copy is the source data and weight documentation will be agreed by participant signature.

Participants will complete questionnaires at baseline and at 6 and 12M follow-up. If these are completed electronically, the electronic record will be considered to be source data. If a hard copy of these questionnaires is completed, these will be considered the source document.

For all other data collected, if these are completed electronically (without hard copy CRF), the electronic record is considered the source data. If the data are collected on a hard copy CRF, this will be considered the source.

The measures are detailed in Section 6.6, with references provided to standardised and non-standard tools.

10.2. Data handling and record keeping

The electronic data capture system (eCRF) is validated, maintains a full audit trail of data changes, is secure (requiring unique user-names and passwords), and has regular back-up. The system safeguards the blinding of trial data. Participants have a unique participant identification number that allows identification of all data reported for each participant.

Clinical data are entered into the database by the designated team members working at each centre. Questionnaires may be completed by participants directly into the study website. If they are completed as a hard copy, data are entered into the database by the designated team members working at each centre. Staff in the trial office work closely with local PIs and Fieldworkers to ensure that the data are as complete and accurate as possible. Extensive range and consistency checks will be performed to further enhance the quality of the data.

10.3. Access to Data

Direct access will be granted to authorised representatives from the Sponsor, host institution and the regulatory authorities to permit trial-related monitoring, audits and inspections- in line with participant consent. Any hard copy data will be stored at Stirling University and requests to access data are administered through the University's data archive DataSTORRE. The investigator site files will be archived at each centre. Following publication of the results, an anonymised participant level dataset and statistical code for generating the results will be available.

10.4. Archiving

Archiving will be authorised by the Sponsor following submission of the end of trial report. All study documentation will be kept for at least 10 years after publication of the study data. Copies of consent forms will be forwarded to the trial office on a regular basis. At the end of each participant's follow-up, case report forms and questionnaires will be returned for archiving in Stirling. The centre files will be archived at each centre.

10.5. Monitoring, Audit & Inspection

The trial will be monitored to ensure that it is being conducted as per protocol, adhering to the UK Policy Framework for Health and Social Care Research, the principles of GCP, and all other appropriate regulations. The approach to, and extent of, monitoring (specifying both central and on-site monitoring) will be specified in a trial monitoring plan which is usually initially determined by a risk assessment, undertaken prior to start of trial. Investigators and their host Institutions will be required to permit trial

related monitoring and audits to take place by the Sponsor and/ or regulatory representatives providing direct access to source data and documents as requested.

11. ETHICAL AND REGULATORY CONSIDERATIONS

11.1. Research Ethics Committee (REC) review & reports

Before the start of the trial, approval will be sought from a REC for the trial protocol, informed consent forms and other relevant documents e.g. advertisements and GP information letters. Amendments to documentation should not be implemented until appropriate approvals are in place.
All correspondence with the REC will be retained in the Trial Master File.

The Chief Investigator and trial managers will be responsible for producing the annual reports for NIHR and to the REC and will notify the REC of the end of the trial, or if the trial ends prematurely.

11.2. Summary of ethical issues

This is a low risk study, where participants could gain benefit to their own health through losing weight and gain financial benefit if they are randomised to the incentive group. The main ethical issues are:

1. Ensuring that the incentives are benefiting the health of the individual and the wider public, in terms of the new knowledge that will be generated about how to engage men in weight loss activities, initiate and maintain weight loss. We aim to recruit men in difficult life situations, who do not have many opportunities and who often do not engage in health promoting activities. In particular ensuring that the information leaflet promotes the equipoise essential for a randomised controlled trial and minimises disappointment bias. For this reason, the amounts of the incentives will not be disclosed in the information leaflet and will only be revealed after randomisation to the incentive arm. Our PPI and third sector stakeholders have helped us design this approach. Every effort will be made to ensure that the incentives are not unduly persuasive or coercive.

2. Safeguarding for research participants is important. This is a low risk study, however there is a possibility that men who are unsuccessful in their weight loss attempts may become upset and this could impact on their emotional well-being. The researchers will be sensitive to this. In very rare circumstances, if the safety of the participant or of another person was thought to be at risk, the researcher would breach confidentiality (for example, if a man tells a researcher that he feels suicidal). Under these circumstances, the researcher would speak to the study leads or a senior member of staff in the host institution as soon as possible and in discussion with the participant, the next steps would be agreed. The Chief Investigator, Prof Pat Hoddinott, is a GP and is therefore trained to manage such situations and Co-Investigator Alison Avenell is an NHS Clinician specialising in Obesity management. Participant information leaflets (web or print) and SMS information which will provide local and national contacts where men can access additional support can be provided (see Section 8.8).

3. Safeguarding the safety of researchers is also important (see Section 8.8). The Lone Worker Policy of the employing university will be followed which includes informing a member of staff where and when

research tasks are being carried out in the community, contact details and agreed report back times. Current government guidelines with regard to Covid-19 (e.g. wearing of face masks) will be adhered to by Fieldworkers and participants.

4. Informed consent. We will adhere to the principles of the Declaration of Helsinki, the Health Research Authority (HRA) guidance (http://www.hra.nhs.uk/). Research Governance procedures according to the University of Stirling will be adhered to: http://www.stir.ac.uk/research/integritygovernanceethics/ and NHS R&D approvals will be in place before any NHS patients are recruited. The team have conducted research on financial incentives previously and are aware of the importance of avoiding perceived coercion, bribery and offering an ethical control arm which allows freedom of choice regarding weight loss. Men's Health Forum and our PPI group have chosen the study short name, helped us draft participant information leaflets, text messages and the website.

11.3. Peer review

This NIHR funded study has been extensively peer reviewed by independent experts, and by the NIHR PHR Board on two occasions – at outline and full application stages. The Game of Stones Feasibility Study was similarly funded by NIHR and extensively independently peer reviewed, with the final report published in the NIHR Journals Library [2]. University of Stirling, the Sponsor, also required internal peer review by two experts prior to grant application.

All reports of work arising from the GoS trial including conference abstracts will be peer reviewed by the Project Management Group prior to submission, with approval to proceed decided by consensus where possible, in line with the funding contract.

11.4. Public and Patient Involvement (PPI)

11.4.1 PPI contributing to the development of this protocol

Continuous and responsive Public and Patient Involvement (PPI) with Men's Health Forum (MHF) Charities commenced in 2011 for our ROMEO systematic reviews on obesity in men [11], continued for the Game of Stones Feasibility Study and continues in this proposed trial. Co-Investigators Martin Tod (England) and Colin Fowler with collaborator Paula Carroll (Ireland) will continue to provide invaluable contributions. To date they have attended Game of Stones Feasibility Study management, grant application protocol writing meetings, commented on study documents, advised on dissemination and co-authored outputs. They have reached out to their networks of men, to engage wider involvement when required. In addition, the University of Stirling PPI Group have commented on the lay summary.

For the completed Feasibility Study, two independent men (one with weight loss experience; one with experience of involving underserved populations) contributed PPI oversight and advice via the Trial Steering Committee (TSC) and therefore were involved in assessing that the progression criteria for this

full trial were met. For detail of the co-production approach to the design of Game of Stones see the final report to NIHR [2] and publications [1]. In summary, a total of 121 PPI contributors helped in a range of activities leading to this protocol including: selecting the study name; refining the language of SMS, information leaflets and web materials; commenting on the trial design particularly an acceptable control group; advising on venues for recruitment and assessment; a Workshop where Feasibility Study design decisions were finalised with PPI and other stakeholders; and two dissemination events to inform this protocol. This diversity of PPI has ensured trial materials and processes are optimal for acceptability to men with obesity, maximise engagement and follow up, particularly those from underserved populations.

Men have requested wellbeing to be included as a secondary outcome, as men describe how feeling “unwell” was a motivator for change and feeling better after losing weight and being more active was important to them. Another PPI suggestion is for men to be able to upload selfie photos to their personal self-monitoring web page throughout the study.

11.4.2 How PPI will be actively involved in this trial

Two PPI representatives are members of the TSC. MHF Co-Investigators will continue to be involved in the same way as they were in the Feasibility Study. They will: contribute to decision making at grant holder meetings; comment on trial documents; provide expertise on communicating with men; access wider networks of men for specific feedback on study materials; assist with interpretation and reporting of findings; co-author outputs and assist with dissemination.

In particular, MHF/MHFI have language experts who will help us finalise the SMS and revise the website. The MHF have written Man Manuals (https://shop.menshealthforum.org.uk/collections/man-manuals/) on weight loss, diabetes, “Man MOT”, serious drinking and beating stress that are relevant to participants in our study. A website link to the MHF/MHFI home page will be on the study website and relevant links will be embedded within SMS. MHF will advise on digital health developments relevant to men’s health particularly around self-monitoring.

Several PPI volunteers in our Feasibility Study have expressed interest in continuing involvement. Claire Torrens, Research Fellow will work with staff across trial centres to co-ordinate PPI contributions. The team will benefit from a Research Partnership Group at The Nursing Midwifery and Allied Health Professionals Research Unit (NMAHP-RU). The Research Partnership Group has a remit to advise on all aspects of PPI for our studies and includes people providing PPI on major UK funding panels. This includes advice on training in line with NIHR Involve resources https://www.invo.org.uk/resource-centre/training-resource/, mentoring and reimbursing people providing PPI.

Two men have agreed to contribute as independent lay members on the TSC: David Gardiner, who is Chairman of Scottish Men's Sheds and Chair of N. Ayrshire Patient Participation Group, and Graham Jameson, who was a lay member on the TSC for the Football Fans in Training Trial (FFIT).

At the dissemination stage, MHF will assist with developing and executing a dissemination strategy for the trial findings. They have strong links with Public Health in the devolved Governments and are well connected to diverse communities of men.

11.5. Protocol compliance and GCP

Researchers will adhere to Good Clinical Practice Guidance and receive appropriate training.

Participants will not be able to enrol if they do not meet the eligibility criteria or if they meet one or more of the exclusion criteria. As the interventions are delivered through an automated computer programme, crossover is very unlikely to occur.

A "serious breach" is a breach of protocol which is likely to effect to a significant degree –

(a) the safety or physical or mental integrity of the participants of the trial; or
(b) the scientific value of the trial

The Sponsor will be notified immediately of any case where the above definition applies.

11.6. Data protection and patient confidentiality

Patient confidentiality will be maintained for all collected data. All investigators and trial site staff will comply with the requirements of the General Data Protection Regulation (GDPR) (or subsequent legislation), with regards to the collection, storage, processing and disclosure of personal information and will uphold the Act's core principles.

Computers used to collate the data, and the trial website will have limited access measures via usernames and passwords. Staff at sites only have access to participant data for participants at their site. Within the study website, identifiable data is stored with a strong encryption algorithm (currently the key used is AES_256). Participants will be identified using a unique participant ID number.

Participant data, contact information and responses to text messages will be managed electronically in the Participant Tracker Software at the Health Informatics Centre, University of Dundee which is an approved NHS safe haven for data. Data handling and storage will comply with GDPR legislation. Researchers employed by the three trial centres (Universities of Stirling, Bristol and Queens Belfast) will have secure passwords to access the Participant Tracker system at the University of Dundee.

Data from qualitative interviews will be anonymised by removing any information which could potentially identify the participant. Only interview transcribers who are approved by the University of Stirling and

meet confidential data handling requirements will be used. Each participant will have a unique participant ID number. The interview recordings, transcriptions and NVivo database will be password protected and encrypted and stored securely at the University of Stirling. The recorder will be wiped clean as soon as the recording has been stored. Personal data and audio recordings will be destroyed as soon as it is certain that they are no longer required (i.e. at the end of the study/follow-up period).

11.7. Financial and other competing interests

The Chief Investigators, Principal Investigators, grant holders, trial staff and TSC members declare no conflicts of interest at the time of writing the protocol. Conflicts of interest will be reviewed during the trial and will be collected for new staff and PIs and reported to the TSC, the Sponsor and funder.

11.8. Indemnity

The University of Stirling, as Sponsor, has a specialist insurance policy to cover any participant suffering harm as a result of participating in this study. This is a NIHR PHR funded self-care trial and no clinical care is provided to participants.

11.9. Amendments

Substantial and non-substantial amendments will be discussed with the Project Management Team, and when appropriate with the independent TSC. HRA guidance will be referred to http://www.hra.nhs.uk/resources/after-you-apply/amendments/

Amendments submitted to the approving REC will be communicated to the participating centres (R&D office and local research team) to assess whether the amendment affects the NHS permission for that site and to the Funder.

The Chief Investigator will be responsible for the decision to amend the protocol and ensure that substantive changes are communicated to relevant stakeholders (e.g., REC, trial registries, R&D, regulatory agencies, funder).

The amendment history will be documented in the Protocol (see Section ii) to enable the most recent protocol version to be identified. A current, up to date version of the Protocol will be provided to all sites, relevant members of the trial team, members of the PMG and TSC.

11.10. Access to the final trial dataset

At the end of the trial, the trial statisticians and health economist will have access to the full dataset to permit analysis. Requests for other access to the full dataset will be considered by the Trial Steering Committee and the Sponsor. An anonymised participant level dataset and statistical code for generating the results will be available.

12. DISSEMINATION POLICY

12.1. Dissemination policy

The Consort Guidelines and checklist will guide the reporting in any publications for the trial to ensure they meet the standards required for submission to high quality peer reviewed journals. http://www.consort-statement.org/

On completion of the trial, the data will be analysed and tabulated in a full trial report. This will be published open access in the NIHR Journals Library. The funders, NIHR will be acknowledged within the publications using their advised wording.

The analysis of the primary outcome effectiveness and cost-effectiveness data will be reported separately and before the results of exploratory hypothesis generating sub-group and mixed methods analyses for: recruitment strategy, health inequalities, mental health, multiple long-term conditions and co-morbidities. These will be reported in separate chapters in the NIHR Journals Library publication.

Publications of the findings will be available on the study website and participants who express a wish to be informed of the outcome of the trial, will be notified by their chosen method (SMS, email, post).

The trial protocol and full trial report will be made publicly available via the NIHR Journals Library.

Our dissemination and engagement strategy will be guided by Men's Health Forum, Men's Sheds and the NIHR Dissemination guidance. Active engagement via face to face meetings, various media and networks will aim for our findings to reach men, the general public, policy makers, NHS commissioners, practitioners and health care staff. Publications will be promoted on social media via Twitter.

We will produce outputs: for a range of audiences including study website updates; dissemination events for stakeholders; peer reviewed publications, international (e.g. European and International Congress on Obesity) and UK (e.g. NHS Confederation, RCGP, Public Health, UK Society for Behavioural Medicine) conference presentations. We propose four high quality peer review publications: i) the main findings paper (e.g. *Lancet*); ii) health economic outcomes (e.g. *Plos Medicine*); ii) mechanisms of action informed by men's experiences during and after the intervention (e.g. *Social Science and Medicine*); iii) Lessons for Implementation (e.g. *Implementation Science*). Game of Stones will be a new open source digital intervention freely available to the NHS (primary or secondary care; local, regional or national); local authorities and not for profit public services e.g. charities. The trial dataset, with the potential addition of long-term data linkage to health outcomes, could inform government policy.

We will engage patients, the NHS and wider public via a lay summary, You-Tube video; social media (Twitter; Facebook) with the help of Men's Health Forum; Men's Sheds; third sector e.g. Diabetes UK, British Heart Foundation; RCGP and Directors of Public Health.

Our outputs will enter the health care system as a whole: by writing briefing papers for Government (e.g. Department of Health and Social Care; NHS Health Scotland); Public Health Umbrella Organisations for the devolved countries (e.g. Public Health England). NICE and SIGN will receive our findings so that they can contribute to relevant obesity guidance. Depending on the results, a strategy 'how to' guide to build capacity within health care system to implement GoS at scale may be produced.

Further funding required if an intervention is effective. By collaborating with digital health programming experts and commissioners our interventions will be ready for widespread implementation.

The CHaRT team will look produce a short summary report based on questions from the PMG and/or TSC on the future roll out of the intervention.

Further NIHR funding will be required if the TSC recommend long term data linkage to health outcomes and further economic evaluation, or if an implementation evaluation of effectiveness and cost-effectiveness in women is indicated to provide equity of opportunity.

The barriers for further research, adoption and implementation will be dependent on Public Health and NHS funding for obesity interventions. In England, Local Authorities are responsible for Tier 1 and 2 weight management services; Primary Care Commissioning for Tier 3 services; Secondary Care for Tier 4 services. In Scotland and N Ireland, NHS Directors of Public Health are responsible for all tiers of weight management service. Our self-care intervention is unusual as it puts men at the centre regardless of current weight management service providers.

Digital technology, smart phones, smart scales, wearable devices for self-monitoring and data linkage together with the required governance, ethics and regulation are evolving rapidly. By working closely with expert digital collaborators, we hope to minimise any future barriers for integrating SMS with or without incentives into the NHS and not for profit public services.

Intellectual property (IP): the Game of Stones IP resides with the University of Stirling; the background IP for the SMS delivery software resides with University of Dundee. A collaboration agreement is in place.

The impact of our research potentially will be: an effective and cost-effective self-care intervention that reduces obesity prevalence in men, obesity-related morbidities, and which will benefit men's health and wellbeing, with benefits for families, social networks and the NHS.

12.2. Authorship eligibility guidelines and any intended use of professional writers

Ownership of the data arising from this study resides with the study team and in accordance with the signed Study Collaboration Agreement. On completion of the study, the study data will be analysed and

tabulated. A detailed report for the funder will be prepared. A detailed publication policy will be produced and approved by the PMG and TSC.

The ICJME has recommended the following criteria for authorship; these criteria are appropriate for journals that distinguish authors from other contributors:

- Authorship credit will be based on 1) substantial contributions to conception and design, acquisition of data, or analysis and interpretation of data; 2) drafting the article or revising it critically for important intellectual content; and 3) final approval of the version to be published. Authors should meet conditions 1, 2, and 3.
- As a large, multicentre group will conduct the work, the group will identify the individuals who accept direct responsibility for the manuscript. These individuals should fully meet the criteria for authorship/contributors defined above, and editors will ask these individuals to complete journal-specific author and conflict-of-interest disclosure forms. When submitting a manuscript authored by a group, the corresponding author will clearly indicate the preferred citation and identify all individual authors as well as the group name. Journals generally list other members of the group in the Acknowledgments. The National Library of Medicine indexes the group name and the names of individuals the group has identified as being directly responsible for the manuscript; it also lists the names of collaborators if they are listed in Acknowledgments.
- Acquisition of funding, collection of data, or general supervision of the research group alone does not constitute authorship.
- All persons designated as authors should qualify for authorship, and all those who qualify should be listed.
- Each author should have participated sufficiently in the work to take public responsibility for appropriate portions of the content.

Contractual Obligations with NIHR for project outputs

A 'project output' is any item arising from NIHR-funded research that enters the public domain. Project outputs can be written, audio/visual, electronic or verbally presented. The NIHR takes a broad definition of project outputs, these might include, but are not limited to:

- written outputs such as press releases, research reports, journal articles, abstracts, presentation slides, posters, websites, books or book chapters, blogs and other forms of social media, newsletters
- presentations, speaking at events or in the media including media interviews
- software/algorithms

- training materials such as manuals or DVDs
- checklists, scales, protocols, questionnaires, toolkits
- service guidelines or similar
- service innovations or new service delivery models
- research tools such as data analysis techniques, assays, cell lines, antibodies, biomarkers
- patentable inventions such as new therapeutic products, equipment, diagnostic test, devices
- participant materials.

NIHR are required to track project outputs throughout your project's progress, and for 5 years after publication of the final report. As well as allowing the Department of Health to prepare an informed response in case of media or political interest, it keeps us informed of the reach of your findings and their impact and helps our own communications work.

All published material must contain an acknowledgement of funding, and when mentioning research findings or opinions, an appropriate disclaimer. The current disclaimers can be found on the NIHR website.

Notification of outputs

The Chief Investigator or Trial Manger will submit an output notification of any research output at the time of submission or at least 28 days before the publication date, whichever is earlier. The full final version of the output should either be submitted as soon as it becomes available.

13. REFERENCES

1. Dombrowski, S.U., et al., *Game of Stones: feasibility randomised controlled trial of how to engage men with obesity in text message and incentive interventions for weight loss.* BMJ Open, 2020. **10**.
2. Dombrowski, S.U., McDonald M, van der Pol M, Grindle M, Avenell A, Carroll P, et al., *Text messaging and financial incentives to encourage weight loss in men with obesity: the Game of Stones feasibility RCT.* Public Health Research, 2020. **8**(11).
3. Wang, Y., et al., *Do men consult less than women? An analysis of routinely collected UK general practice data.* BMJ Open, 2013. **3**(8): p. e003320.
4. Steel, N., et al., *Changes in health in the countries of the UK and 150 English Local Authority areas 1990–2016: a systematic analysis for the Global Burden of Disease Study 2016.* The Lancet, 2018. **392**(10158): p. 1647-1661.
5. ONS, *Health state life expectancies by national deprivation deciles, England: 2016 to 2018*. 2020.
6. NHS Digital, *Statistics on Obesity, Physical Activity and Diet England: 2018*. 2018, Health and Social Care Information Centre.
7. NHS Digital, *Health Survey for England 2017 Summary of key findings*. 2018, Health and Social Care Information Centre.
8. Michie, S., et al., *Developing and Evaluating Digital Interventions to Promote Behavior Change in Health and Health Care: Recommendations Resulting From an International Workshop.* Journal of Medical Internet Research, 2017. **19**(6).
9. NICE, *Obesity: identification, assessment and management*. 2014, National Institute for Health and Care Excellence.
10. McDonald, M.D., et al., *Recruiting men from across the socioeconomic spectrum via GP registers and community outreach to a weight management feasibility randomised controlled trial.* BMC Medical Research Methodology, 2020. **20**(249).
11. Robertson, C., et al., *Systematic reviews of and integrated report on the quantitative, qualitative and economic evidence base for the management of obesity in men.* HTA, 2014. **18**(35): p. 1-424.
12. Skinner R, G.V., Currie S, Hoddinott P, Dombrowski SU., *A systematic review with meta-analyses of text messagedelivered behaviour change interventions for weight loss and weight loss maintenance.* Obesity Reviews, 2020. **21**.
13. Mantzari, E., et al., *Personal financial incentives for changing habitual health-related behaviors: A systematic review and meta-analysis.* Preventive Medicine, 2015. **75**: p. 75-85.
14. Ananthapavan, J., A. Peterson, and G. Sacks, *Paying people to lose weight: the effectiveness of financial incentives provided by health insurers for the prevention and management of overweight and obesity – a systematic review.* Obesity Reviews, 2018. **19**(5): p. 605-613.
15. Sykes-Muskett, B.J., et al., *The utility of monetary contingency contracts for weight loss: A systematic review and meta-analysis.* Health Psychol Rev, 2015: p. 1-21.
16. Hunt, K., et al., *A gender-sensitised weight loss and healthy living programme for overweight and obese men delivered by Scottish Premier League football clubs (FFIT): A pragmatic randomised controlled trial.* The Lancet, 2014. **383**(9924): p. 1211-1221.

17. Mackenzie, R.M., *Core outcome set for behavioural weight management interventions for adults with overweight and obesity: Standardised reporting of lifestyle weight management interventions to aid evaluation (STAR-LITE).* Obesity Reviews, 2020. **21**.

18. Hagstromer, M., P. Oja, and M. Sjostrom, *The International Physical Activity Questionnaire (IPAQ): a study of concurrent and construct validity.* Public Health Nutr, 2006. **9**(6): p. 755-62.

19. National Institute on Alcohol Abuse and Alcoholism, *Recommended Alcohol Questions 2000*. 2000.

20. Health and Social Care Information Centre, *Health Survey for England 2016*. 2016: London.

21. Tennant, R., et al., *The Warwick-Edinburgh Mental Well-being Scale (WEMWBS): development and UK validation.* Health Qual Life Out, 2007. **5**(1): p. 63.

22. Hartmann-Boyce, J., et al., *Cognitive and behavioural strategies for weight management in overweight adults: Results from the Oxford Food and Activity Behaviours (OxFAB) cohort study.* PLoS ONE, 2018. **13**(8).

23. Best, M. and E.K. Papies, *Lower socioeconomic status is associated with higher intended consumption from oversized portions of unhealthy food.* Apetite, 2019. **140**: p. 255–268.

24. Bridges, S. and R. Disney, *Working Paper 06/02 Debt and Depression*. 2005.

25. French, D., *Financial strain in the United Kingdom.* Oxford Economic Papers, 2018. **70**(1): p. 163-182.

26. Men's Health Forum, *How to make weight-loss services work for men*. 2014, London: Haynes Publishing.

27. Newton, S., D. Braithwaite, and T.F. Akinyemiju, *Socio-economic status over the life course and obesity: Systematic review and meta-analysis.* PLoS One, 2017. **12**(5): p. e0177151.

28. Ofcom, *Adults' Media Use and Attitudes Report*, Ofcom, Editor. 2018: London.

29. Ahern, A.L., et al., *Inequalities in the uptake of weight management interventions in a pragmatic trial: an observational study in primary care.* Br J Gen Pract, 2016. **66**(645): p. e258-63.

30. The Food Foundation, *THE BROKEN PLATE 2020 The State of the Nation's Food System*. 2020, The Food Foundation.

31. Crombie, I.K., et al., *Alcohol and disadvantaged men: A feasibility trial of an intervention delivered by mobile phone.* Drug and Alcohol Review, 2017. **36**(4): p. 468-476.

32. Crombie, I.K., et al., *Texting to Reduce Alcohol Misuse (TRAM): main findings from a randomized controlled trial of a text message intervention to reduce binge drinking among disadvantaged men.* Addiction, 2018. **13**(9): p. 1609–1618.

33. Irvine, L., et al., *Modifying alcohol consumption to reduce obesity: A randomized controlled feasibility study of a complex community-based intervention for men.* Alcohol and Alcoholism, 2017. **52**(6): p. 677-684.

34. Semaan, S., *Time-space sampling and respondent-driven sampling with hard-to-reach populations.* Methodological Innov Online, 2010. **5**: p. 60 - 75.

35. Schulz, K.F., D.G. Altman, and D. Moher, *CONSORT 2010 Statement Updated Guidelines for Reporting Parallel Group Randomized Trials.* Obstetrics and Gynaecology, 2010. **115**(5): p. 1063-1070.

36. Parkinson, B., et al., *Designing and using incentives to support recruitment and retention in clinical trials: a scoping review and a checklist for design.* Trials, 2019. **20**(1): p. 624.

37. Elfeky, A., et al., *Non-randomised evaluations of strategies to increase participant retention in randomised controlled trials: a systematic review.* Syst Rev, 2020. **9**(1): p. 224.

38. Hoffmann, T.C., et al., *Better reporting of interventions: Template for intervention description and replication (TIDieR) checklist and guide.* BMJ, 2014. **348**.

39. Men's Health Forum, *Mind your language: How men talk about mental health*. 2018, London.

40. Schwarzer, R., *Modeling health behavior change: How to predict and modify the adoption and maintenance of health behaviors.* Appl Psychol, 2008. **57**(1): p. 1-29.

41. Deci, E.L., R.M. Ryan, and R. Koestner, *A meta-analytic review of experiments examining the effects of extrinsic rewards on intrinsic motivation.* Psychological Bulletin, 1999. **125**(6): p. 627-668.

42. Kwasnicka, D., et al., *Theoretical explanations for maintenance of behaviour change: a systematic review of behaviour theories.* Health Psychology Review, 2016. **10**(3): p. 277-296.

43. van Beurden, S.B., et al., *Techniques for modifying impulsive processes associated with unhealthy eating: A systematic review.* Health Psychol, 2016. **35**(8): p. 793-806.

44. Yardley, L., et al., *The person-based approach to intervention development: application to digital health-related behavior change interventions.* J Med Internet Res, 2015. **17**(1): p. e30.

45. Archibald, D., et al., *A qualitative evidence synthesis on the management of male obesity.* BMJ Open, 2015. **5**(10): p. e008372.

46. Camerer, C.F., G. Loewenstein, and M. Rabin, *Advances in Behavioral Economics*. 2003: Princeton University Press.

47. Cawley, J., J.S. Downs, and G. Loewenstein, *Behavioral Economics and Obesity*. 2012: Oxford University Press.

48. Giles, E.L., et al., *The effectiveness of financial incentives for health behaviour change: Systematic review and meta-analysis.* PLoS ONE, 2014. **9**(3): p. e90347.

49. Purnell, J.Q., et al., *A systematic review of financial incentives for dietary behavior change.* Journal of the Academy of Nutrition and Dietetics, 2014. **114**(7): p. 1023-1035.

50. Paloyo, A.R., et al., *The causal link between financial incentives and weight loss: an evidence-based survey of the literature.* Journal of Economic Surveys, 2014. **28**(3): p. 401-420.

51. Jochelson, K., *Paying the patient.* Improving health using financial incentives. King's Fund, 2007.

52. Gamble, C., et al., *Health Services and Delivery Research*, in *An evidence base to optimise methods for involving patient and public contributors in clinical trials: a mixed-methods study*. 2015, NIHR Journals Library: Southampton (UK).

53. van Hoorn, R., et al., *The development of CHAMP: a checklist for the appraisal of moderators and predictors.* BMC Med Res Methodol, 2017. **17**(1): p. 173.

54. Abel, G.A., M.E. Barclay, and R.A. Payne, *Adjusted indices of multiple deprivation to enable comparisons within and between constituent countries of the UK including an illustration using mortality rates.* BMJ Open, 2016. **6**(11): p. e012750.

55. Avenell, A., et al., *Bariatric surgery, lifestyle interventions and orlistat for severe obesity: the REBALANCE mixed-methods systematic review and economic evaluation.* Health Technol Assess, 2018. **22**(68): p. 1-246.

56. Kent, S., et al., *Is Doctor Referral to a Low-Energy Total Diet Replacement Program Cost-Effective for the Routine Treatment of Obesity?* Obesity (Silver Spring), 2019. **27**(3): p. 391-398.

57. Moore, G.F., et al., *Process evaluation of complex interventions: Medical Research Council guidance.* BMJ, 2015. **350**: p. h1258.

58. Pfadenhauer, L.M., et al., *Making sense of complexity in context and implementation: the Context and Implementation of Complex Interventions (CICI) framework.* Implement Sci, 2017. **12**(1): p. 21.

59. Hunt, K., et al., *"You've got to walk before you run": positive evaluations of a walking program as part of a gender-sensitized, weight-management program delivered to men through professional football clubs.* Health Psychol, 2013. **32**(1): p. 57-65.

60. Ritchie, J. and J. Lewis, *Qualitative Research Practice: A Guide for Social Science Students and Researchers*. 2003, London: SAGE.

61. Samdal, G.B., et al., *Effective behaviour change techniques for physical activity and healthy eating in overweight and obese adults; systematic review and meta-regression analyses.* Int J Behav Nutr Phys Act, 2017. **14**(1): p. 42.

62. Lillis, J., et al., *Measuring Weight Self-stigma: The Weight Self-stigma Questionnaire.* Obesity, 2010. **18**(5): p. 971-976.

63. Mercer, S.W., et al., *The consultation and relational empathy (CARE) measure: development and preliminary validation and reliability of an empathy-based consultation process measure.* Family Practice, 2004. **21**(6): p. 699-705.

64. Bacon, J., K. Scheltema, and B. Robinson, *Fat phobia scale revisited: the short form.* International Journal of Obesity, 2001. **25**: p. 252-257.

65. Reniers, R.L.E.P., et al., *The QCAE: a Questionnaire of Cognitive and Affective Empathy.* Journal of Personality Assessment, 2011. **93**(1): p. 84-95.

66. Hatcher, R.L. and J.A. Gillaspy, *Development and validation of a revised short version of the working alliance inventory.* Psychotherapy Research, 2006. **16**(1): p. 12-25.

67. Emile Pereira-Miranda, Priscila R. F. Costa, Valterlinda A. O. Queiroz, Marcos Pereira-Santos & Mônica L. P. Santana (2017) *Overweight and Obesity Associated with Higher Depression Prevalence in Adults: A Systematic Review and Meta-Analysis, Journal of the American College of Nutrition*, **36**:3, 223-233, DOI: https://doi.org/10.1080/07315724.2016.1261053 (accessed April 2022)

68. Gariepy, G., Nitka, D. & Schmitz, N. *The association between obesity and anxiety disorders in the population: a systematic review and meta-analysis*. Int J Obes **34**, 407–419 (2010). https://doi.org/10.1038/ijo.2009.252 (accessed April 2022)

69. Marmot, M., Allen, J., Goldblatt, P., Herd, E., and Morrison, J. *Build Back Fairer: The COVID-19 Marmot Review*, The Health Foundation, December 2020, DOI: https://www.health.org.uk/publications/build-back-fairer-the-covid-19-marmot-review (accessed April 2022)

70. Firth, J. et al, *The Lancet Psychiatry Commission: a blueprint for protecting physical health in people with mental illness*,The Lancet Psychiatry, Volume **6**, Issue 8, 2019, Pages 675-712, ISSN 2215-0366, https://doi.org/10.1016/S2215-0366(19)30132-4 (accessed April 2022)

71. Dombrowski S, U, Avenell A, Sniehott F, F: *Behavioural Interventions for Obese Adults with Additional Risk Factors for Morbidity: Systematic Review of Effects on Behaviour, Weight and Disease Risk Factors*. Obes Facts 2010;**3**:377-396. doi: https://doi.org/10.1159/000323076 (accessed April 2022)

72. Kraef C, van der Meirschen M, *Free CDigital telemedicine interventions for patients with multimorbidity: a systematic review and meta-analysis,*BMJ Open 2020;**10**:e036904. doi: http://dx.doi.org/10.1136/bmjopen-2020-036904 (accessed April 2022)

73. Nowakowska, M., Zghebi, S.S., Ashcroft, D.M. et al. *The comorbidity burden of type 2 diabetes mellitus: patterns, clusters and predictions from a large English primary care cohort*. BMC Med **17**, 145 (2019). https://doi.org/10.1186/s12916-019-1373-y (accessed April 2022)

74. UK Parliament, House of Commons Library, Briefing Paper, *Mental health statistics: prevalence, services and funding in England*, Published 13 December, 2021, DOI: https://commonslibrary.parliament.uk/research-briefings/sn06988/(accessed April 2022)
