# Supplementary material for: Effectiveness and cost-effectiveness of text messages with or without endowment incentives for weight management in men with obesity (Game of Stones): study protocol for a randomised controlled trial
Source: Trials. 2022 Jul 22;23:582. doi: 10.1186/s13063-022-06504-5 (PMC9306253; doi:10.1186/s13063-022-06504-5)
Supplement: Supplementary file 4 — Additional file 4. GoS Full Participant Information Leafletv2.0_29.09.21 (pdf): The full participant information leaflet for Game ofStones. [file 13063_2022_6504_MOESM4_ESM.pdf]

Game of Stones:
Helping Men to Lose Weight

Are you a man who wants to lose weight?

This research may interest you…

About Game of Stones
Some men want to lose some weight, and this in turn can make you feel better and help to reduce the risk of many health problems. This text messaging and incentive research has been designed with men and for men. It aims to help men to lose weight and keep it off for at least a year.

Who can take part?
- Men aged 18 and above
- Men whose weight could affect their future health. This is when a Body Mass Index (a measure of weight by height) is 30 or more and our researchers can calculate this for you
- Men who have access to a mobile phone and can receive text messages
- Men who can read English.

What will happen if I take part?
You would:
- Get a step-counter (pedometer)
- Get a password for a research website with information and links about how to lose weight
- Be allocated by chance by a computer to one of three research groups
- Get text messages to help you to lose weight. You will get these messages this year or next and be weighed 3 or 5 times depending on which group you are allocated to
- Attend appointments with a researcher to be weighed and measured at the start of the research and after 3, 6, 12 and 24 months. You will also be asked to answer a few questions about your experience of taking part in the research
- Receive a £20 voucher after attending the 12 and 24 month appointment
- Have a one in three chance to be given a money reward if you achieve personal weight loss targets after 12 months
- Attend appointments at a place that suits you and local Covid-19 guidance will be followed. Appointments will last about 30 to 60 minutes at the start, 12 and 24 months
- Depending on which group you are allocated to, the discussion at 3 and 6 month 'weigh-in' appointments will vary in the questions asked and last 5 to 10 minutes
- You may be invited to share your experiences of taking part in the trial in more detail in an interview with a researcher. You will be given a separate information leaflet about this nearer the time.

Do I have to take part in the research?
No. If you are interested in taking part in Game of Stones, researchers will meet/call you to tell you about the research and to answer any questions you may have. You can then decide whether or not

This trial is funded by the National Institute for Health Research's
PHR Programme (Ref: NIHR129703). IRAS: 290955

to take part. If you decide to take part, you can change your mind at any time. Whatever your decision, your health care will not be affected.

What are the benefits of taking part and are there any disadvantages?
If you take part, you will get help to lose weight, however you may be on a 12 month waiting list for the text messages. You can choose how you lose weight and which information you follow. The main disadvantage of taking part is that losing weight is hard work and it can be upsetting if you don't lose weight. Please come back for all appointments regardless of weight loss results, the research is interested in everybody. You will get a £20 gift voucher at the 12 month and the 24 month appointments to thank you for your time taking part and helping with our research.

Will my participation be confidential?
Yes. Any information that you give us will be kept confidential and will be stored securely on password protected computers and in locked filing cabinets at the participating Universities (Aberdeen, Belfast, Bristol, Glasgow Caledonian, Stirling and Dundee). If a researcher believes that you have told them something that places you or others at serious risk of harm, then the researcher is obliged to pass this information on to someone who could help, for example your GP.

Who is organising and funding the research?

This research is happening in and around Greater Glasgow, Bristol, and Belfast. Members of the public have helped us to design Game of Stones and will continue to advise us. The research team is led by Professor Pat Hoddinott who is based at the University of Stirling. Research experts from the Universities in Aberdeen, Belfast, Bristol, Dundee, and Glasgow, are also involved. The Men's Health Forum and Men's Sheds are supporting our research. Game of Stones is funded by the National Institute of Health Research.

Who has reviewed Game of Stones?
Game of Stones has been reviewed by the North of Scotland (2) Research Ethics Committee.

How will we use information about you?
We will need to use information provided by you for this research. This information will include your name, date of birth and contact details, and may include your bank details if you are in the group receiving the money reward. People will use this information to do the research or to check your records to make sure that the research is being done properly.

People who do not need to know who you are will not be able to see your name or contact details. Your information will have a code number instead. We will keep all information about you safe and secure. Once we have finished the research, we will keep some of the information so we can check the results. We will write reports of the research in a way that no-one can work out that you took part.

What are your choices about how your information is used?

You can stop being part of the research at any time, without giving a reason, but we will keep information about you that we already have. If you choose to stop taking part in Game of Stones, we would like to continue collecting information about your health from central NHS records/ your hospital/ your GP. If you do not want this to happen, tell us and we will stop.
We need to manage your information in specific ways for the research to be reliable. This means that we won't be able to let you see or change the data we hold about you.

Where can you find out more about how your information is used?
You can find out more about how we use your information by asking a member of the research team or contacting Stirling University's Data Protection Officer, Joanna Morrow: data.protection@stir.ac.uk. More information on how information is used in research can be obtained from www.hra.nhs.uk/patientdataandresearch. The researcher can give you a paper copy of this information if you prefer.

How do I contact you?

For more information you can phone a member of the research team on 01786 467491, email at gameofstones@stir.ac.uk or visit the Game of Stones website www.gameofstonesresearch.com.

If you would like to speak to a University member of staff independent to the research please contact Jayne Donaldson by phone 07904635881, email fhss.dean.pa@stir.ac.uk **or by post: 4B157, University of Stirling, Stirling FK9 44A.**

If you need additional help – we suggest the following:
The NHS choices website:
http://www.nhs.uk/LiveWell/Loseweight/Pages/Loseweighthome.aspx
The Men's Health Forum: https://www.menshealthforum.org.uk
If you feel unwell contact the NHS by dialing 111 (free). Or if you need a listening ear for your problems, call The Samaritans on 116 123 or email them on jo@samaritians.org. They are available 24 hours a day, 365 days a year.

This trial is funded by the National Institute for Health Research's PHR Programme (Ref: NIHR129703). IRAS: 290955
